# Functionalisation of ethereal-based saturated heterocycles with concomitant aerobic C–H activation and C–C bond formation†

**DOI:** 10.1039/d2sc01626e

**Published:** 2022-06-27

**Authors:** Nehaal Ahmed, Richard J. Spears, Tom D. Sheppard, Vijay Chudasama

**Affiliations:** Department of Chemistry, University College London 20 Gordon Street London WC1H 0AJ UK v.chudasama@ucl.ac.uk

## Abstract

With an ever-growing emphasis on sustainable synthesis, aerobic C–H activation (the use of oxygen in air to activate C–H bonds) represents a highly attractive conduit for the development of novel synthetic methodologies. Herein, we report the air mediated functionalisation of various saturated heterocycles and ethers *via* aerobically generated radical intermediates to form new C–C bonds using acetylenic and vinyl triflones as radical acceptors. This enables access to a variety of acetylenic and vinyl substituted saturated heterocycles that are rich in synthetic value. Mechanistic studies and control reactions support an aerobic radical-based C–H activation mechanism.

## Abstract 2

Herein we disclose a novel method for the aerobic C–H activation of ethereal-based heterocycles to generate various α-functionalised building blocks.

## Graphical abstract

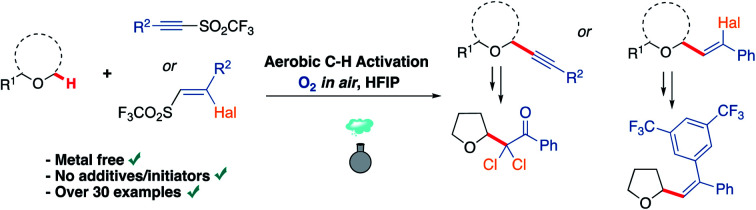

## Introduction

Saturated heterocycles represent important motifs that are present in numerous pharmaceuticals and natural products.^1–5^ In particular, there is a growing interest in the use of sp^3^-rich heterocyclic scaffolds for pharmaceutical applications where they are considered likely to reduce attrition in the drug discovery pipeline.^6–8^ Their widespread use is still held back by difficulties in the synthesis of these molecules from readily available precursors. The direct sp^3^ C–H functionalisation of saturated heterocycles is a potentially valuable strategy that could enable a plethora of compounds to be accessed effectively from simple precursors, with C–C bond formation arguably being the most valuable transformation. However, currently available methods for direct C–H activation of saturated heterocycles require: (i) complicated directing group scaffolds/protecting groups, which necessitate additional installation and removal steps;^9,10^ (ii) the presence of precious and/or toxic transition metal catalysts;^11,12^ (iii) external additives or initiators, occasionally in stoichiometric quantities, that must then be separated from the product;^13,14^ or (iv) high energy materials such as diazo compounds, which can be difficult to handle.^15,16^ To overcome these limitations and improve sustainability, new methodologies that enable simple and sustainable C–H functionalisation of sp^3^-rich heterocycles are required.^17^

**Scheme 1:**
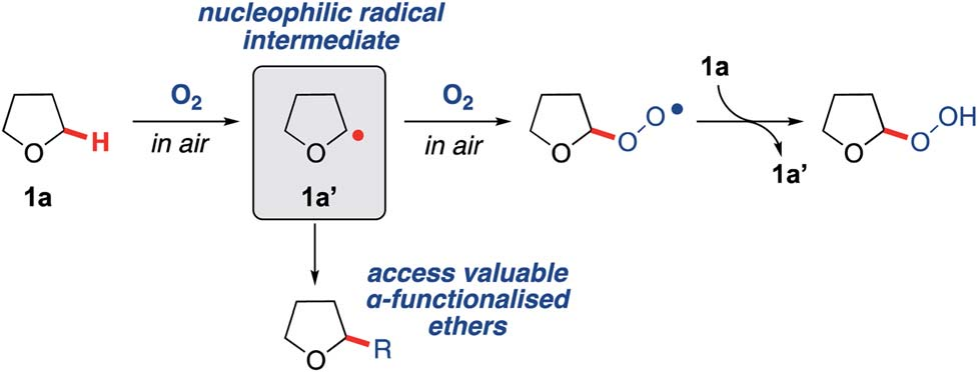
Aerobic oxidation of THF 1a to form a peroxide species *via* reactive nucleophilic radical intermediate 1a′.

**Scheme 2:**
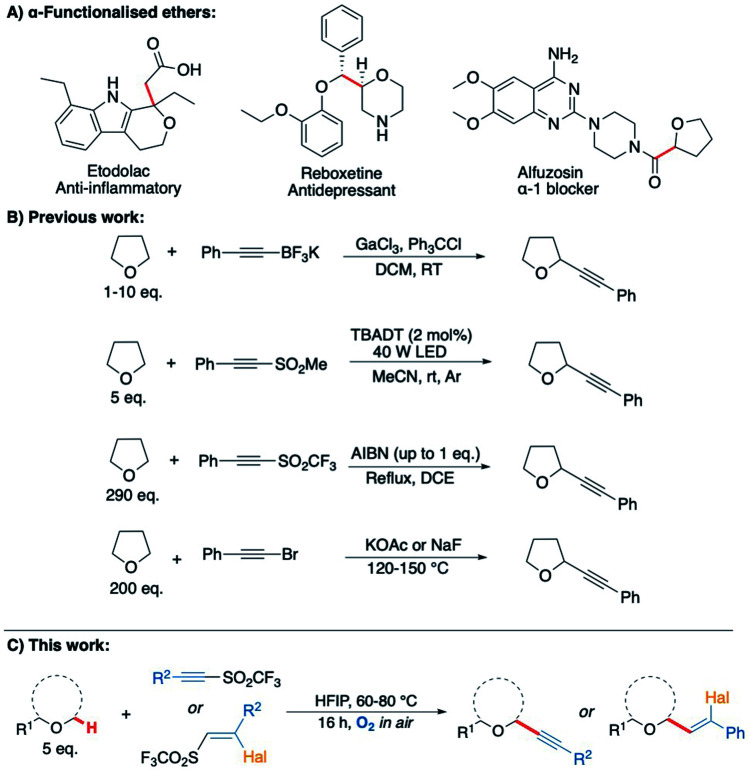
(A) Bioactive α-functionalised ether scaffolds present in pharmaceuticals;^50–52^ (B) previous methods for the alkynylation of THF;^41–47^ (C) this work: C–H alkynylation of ethers *via* aerobic C–H activation.

Radical-based C–H bond activation is a potentially powerful and versatile strategy for the functionalisation of saturated heterocycles.^18–21^ However, existing approaches, whilst effective, typically employ specialised initiators and/or careful management of reaction conditions to achieve this in a controlled fashion.^22–26^ Aerobic C–H activation, utilising oxygen in air to promote radical-based C–H bond cleavage, represents a potentially ideal approach,^26–30^ but to date has not been exploited for the formation of C–C bonds on saturated heterocycles. To explore this idea, we initially examined tetrahydrofuran (THF, 1a) as a candidate for aerobic C–H activation as THF derivatives are highly sought after.^31^ Moreover, the interaction of molecular oxygen with the α-C(sp^3^)–H in THF (as well as many other ethers) is known to result in the generation of a nucleophilic radical intermediate 1a′ (Scheme 1), which then reacts with a second equivalent of oxygen to form a peroxy radical and completes the chain reaction by C–H activation of THF to from a peroxide species, re-generating the radical intermediate 1a′ (Scheme 1).^32–35^ With this as a starting point, we wanted to consider if intermediate 1a′ could instead be trapped with suitable reaction partners in a manner that would establish an alternative chain reaction to afford THF derivatives with a new C–C bond. This could potentially form a general strategy for the preparation of α-functionalised ethers, which are very important moieties in drug discovery, representing more than 20% of the top 200 small molecule drugs in the pharmaceutical industry (selected examples provided in Scheme 2A).^36^

As electron poor alkynes were expected to be highly reactive towards nucleophilic radical 1a′, a range of alkyne reaction partners bearing leavings groups that could propagate a chain reaction cycle were tested.^37,38^ The expected alkynyl-THF products would also provide a synthetically versatile scaffold, which could be readily manipulated to achieve further molecular complexity *via* transformations of the triple bond.^39–42^ We envisaged that the aerobically induced formation of α-functionalised propargylic ethers would proceed *via* a radical addition/elimination reaction exploiting the nucleophilic intermediate 1a′ (Scheme 1). Previous examples of this type of radical reaction between ethers and acetylenic radical acceptors (Scheme 2) have involved the use of stoichiometric amounts of peroxides/initiators, transition metals, specialised photocatalysts or high temperatures (up to 150 °C).^43–47^ By utilising the aerobic oxidation of the ether scaffolds, these additives would be unnecessary. Importantly, we also aimed to avoid the need for a vast excess of the ether traditionally required in this type of reaction,^48,49^ treating ethers more as substrates as opposed to the reaction solvent.

**Scheme 3:**
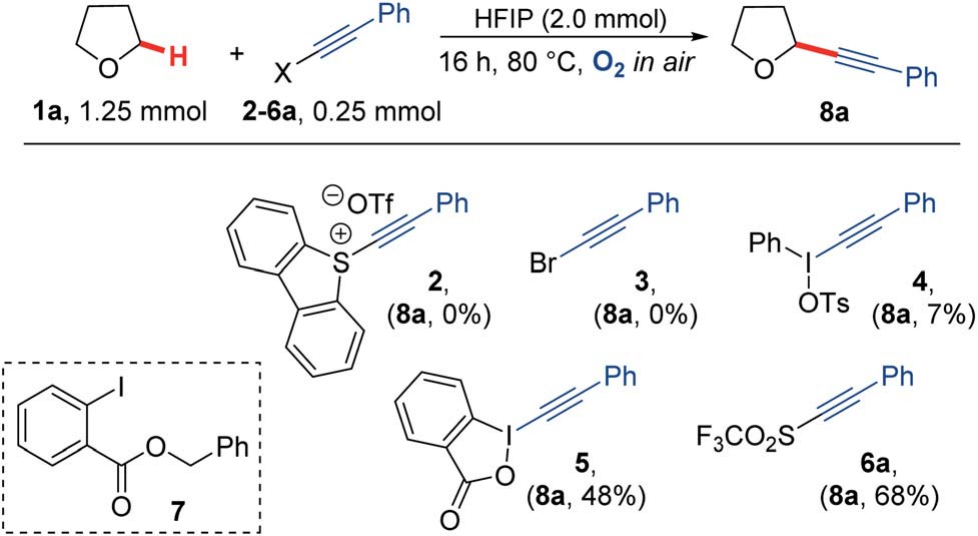
A variety of electron deficient alkenes and alkynes were trialled as radical acceptors for reaction with THF under aerobic conditions.

Our study began by screening a range of acetylenic acceptors (Scheme 3) for trapping the aerobically generated THF radical 1a′ using hexafluoroisopropanol (HFIP) as a co-solvent/additive. These acceptors were selected as they have previously been reported to react with nucleophilic radicals,^53–56^ and the reaction conditions selected were based on our previous work.^29^ Dibenzothiophenium salt 2 had poor solubility in the THF : HFIP mixture, which led to no formation of the desired THF acetylene product under the reaction conditions; this was also the case for bromophenylacetylene 3. Iodonium tosylate 4 was more soluble in the reaction conditions and underwent some reaction, forming the acetylenic THF adduct 8a as the only product, but in only 7% yield due to the low conversion of alkyne. Using Ph-EBX (ethynylbenziodoxolone) 5 as the radical acceptor resulted in an improved yield of the α-C–H functionalised THF product (48%), but a major side product 7, formed *via* the rearrangement of 5, was also isolated.^57^ The most promising results were obtained using acetylenic triflone 6a, which gave a 68% yield of the desired THF adduct 8a. Full conversion of the triflone 6a was observed *via* TLC and crude NMR, and the side-products did not appear to be derived from THF, suggesting some degradation of the triflone under the reaction conditions.

As the reaction of 6a and 1a to form the acetylenic THF product 8a gave the most promising results, this reaction was optimised further (Table 1).^58^ Initially, as we suspected degradation of alkyne 6a under the reaction conditions, triflone 6a was incubated in HFIP (2.0 mmol) over 16 h at 80 °C. This study revealed approximately ∼20% degradation of triflone 6a by NMR (measured using an internal standard). When employing less HFIP (1.0 or 0.4 mmol), degradation was reduced to <10% and <5% (respectively). In view of these results, a smaller amount of HFIP (1.0 and 0.40 mmol) was trialled in the C–H functionalisation reaction (entries 2 and 3), and this afforded increasing amounts of the desired product with a 93% yield of THF-alkyne 8a being obtained with 0.4 mmol of HFIP. In the absence of HFIP, 8a was obtained in only 41% yield (entry 4), demonstrating that the presence of HFIP was beneficial for the formation of THF-alkyne species 8a. We believe that the unique H-bonding abilities of HFIP, which allows THF to form a higher boiling point azeotrope, enables auto-oxidation to take place at a faster rate.^59–62^ Other fluorinated alcohols such as 2,2,2-trifluoroethanol and perfluoro-*tert*-butanol as substitutes for HFIP were also trialled but were found to be less effective (entries 5 and 6). Notably, the mixture of THF and HFIP used under the optimal conditions boiled at a higher temperature (*ca.* 80 °C) than THF alone and, as mentioned, this may be important in increasing the rate of radical formation. Repeating the optimised reaction conditions for the reaction of THF 1a and acetylenic triflone 6a in HFIP (entry 3) under an argon atmosphere whilst bubbling a constant stream of argon through the solution led to no formation of 8a (entry 7) with most of the starting materials being recovered (only 5% consumption of triflone), confirming that the presence of molecular oxygen in air is vital for the formation of the desired product. Attempting the reaction at lower temperatures led to a reduction in the yield of 8a (see ESI Table S1† for details). This was to be expected as the rate of formation of THF radicals is thought to be a function of temperature.^63^ It was also hypothesised that the leaving group ˙SO_2_CF_3_ aids the radical chain process, as decomposition of this radical releases SO_2_ and ˙CF_3_.^64^ The trifluoromethyl radical is highly reactive and polarity matched for efficient H-abstraction from THF leading to an efficient radical chain process.^65^

**Table 1:** Selected optimisation experiments for the reaction of THF 1a and phenyl acetylenic triflone 6a^a^

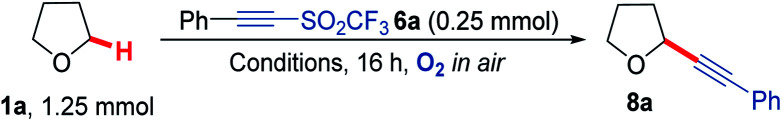				
Entry	*T* (°C)	Solvent/additive	Yield 8a (%)	Conversion of Triflone 6a (%)
1	80	HFIP (2.0 mmol)	68	100
2	80	HFIP (1.0 mmol)	78	100
**3**	**80**	**HFIP (0.40 mmol)**	**93**	**100**
4	66	No HFIP	41	80
5	80	CF_3_CH_2_OH (0.40 mmol)	49	90
6	80	*tert*-C_4_F_9_OH (0.40 mmol)	32	100
7	80	HFIP (0.40 mmol)^b^	0	5

a See ESI for full optimisation table (Table S1).

b Under argon.

**Scheme 4:**
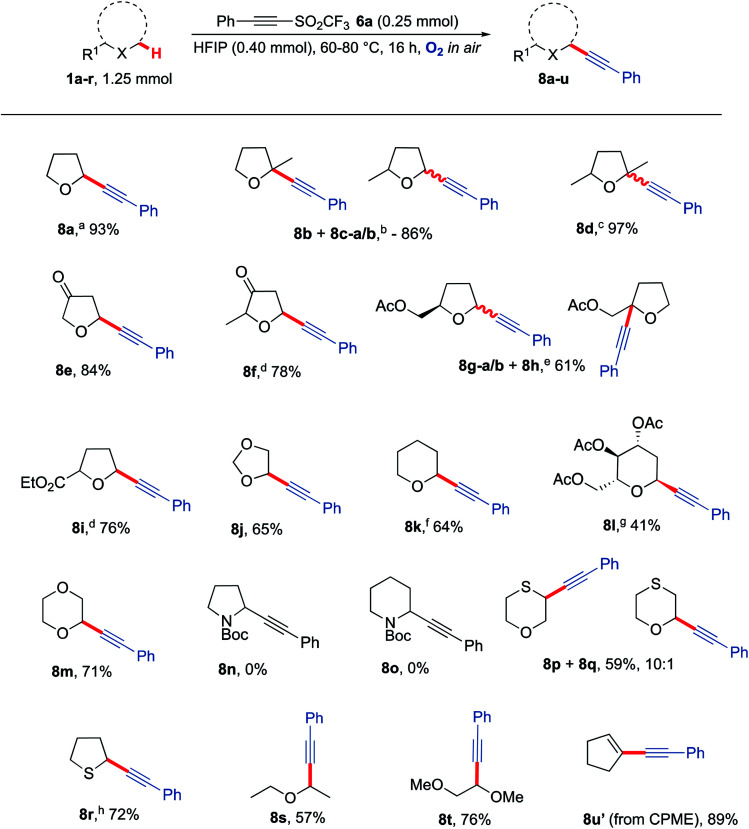
Substrate scope for the reaction of a range of heterocyclic and straight chain ether moieties with alkyne 6a, using the optimised conditions developed in Table 1^a^ 89% yield on a gram scale. ^b^ 1.7 : 1:0.5 8b : 8c–a : 8c–b. ^c^ 3 : 1 mixture of isomers. ^d^ Single stereoisomer obtained. ^e^ 1 : 0.35 : 0.85 8g–a : 8g–b : 8h. ^f^ 30 h, 10 eq. THP. ^g^ Major diastereomer isolated, 99 : 1 dr, 48 h. ^h^ No HFIP, 60 °C. CPME = cyclopentyl methyl ether.

With optimised conditions in hand, the generality of the procedure to α-alkynlate various ethers was then explored (Scheme 4). Ethers were selected based on their likely susceptibility to aerobic activation based on the bond dissociation energy of their α-C–H bond(s).^66–68^ Gratifyingly, many 5-membered THF derivatives provided good to excellent yields of the desired α-alkynlated ethers 8b–8j. Interestingly, no mixture of products was obtained when functionalising 2-methyltetrahydro-3-furanone 1f as compared to 2-methyl THF 1b. The reaction was also compatible with the presence of ketones (8e, 8f) and esters (8g, 8i). Although simple alkyl-substituted ethers do not show much regioselectivity in the reactions, substrates bearing a carbonyl group (either within the THF ring or adjacent to it) typically undergo alkynylation with high regioselectivity with alkynylation not taking place adjacent to the carbonyl group (*i.e.*8e, 8f and 8i). Tetrahydropyran (THP) was readily functionalised to afford 8k in 64% yield and 1,4-dioxane was monofunctionalised in 71% yield to give 8m, thus showcasing compatibility with 6-membered ring systems. A longer reaction time was required for an optimal yield of 8k when using THP as the ether (30 h); this is presumably due to the slower rate of formation of THP α-C(sp^3^)−H radicals.^69^ Applying the methodology to the more challenging 6-membered tri-acylated sugar derivative afforded product 8l as a separable single diastereomer. Unfortunately, no reactivity was observed with *N*-Boc pyrrolidine and *N*-Boc piperidine. Although a similar BDE range is reported for these nitrogen heterocycles (377–385 kJ mol^−1^)^70^ there was no evidence that they are prone to aerobic auto-oxidation under these conditions. Consistent with this observation, *N*-Ph, *N*-Ts and *N*-Bn pyrrolidines and piperidines, did not undergo reaction with triflone 6a under the optimised reaction conditions to form any of the respective alkynylated adducts. Interestingly, the use of thioxane led to a major product where functionalisation had taken place at the sulfur α-C–H position (8p) whilst also giving the corresponding oxygen α-C–H functionalised regioisomer 8q. Tetrahydrothiophene was readily converted to the acetylenic product 8r even in the absence of HFIP; in this particular case the presence of HFIP was in fact undesirable as it was observed to promote an undesired ring-opening reaction.^19^ Straight chain ethers such as diethyl ether and dimethoxyethane also proved to be compatible with the reaction conditions to afford alkynes 8s and 8t (respectively) in good yields. A lower temperature was required for diethyl ether due to its low boiling point; this also demonstrates that lower temperatures can still lead to efficient C–H functionalisation. Finally, using readily available CPME (cyclopentyl methyl ether) led unexpectedly to the formation of enyne 8u′, presumably *via* elimination of methanol from the initial C–H alkynylated product.

**Scheme 5:**
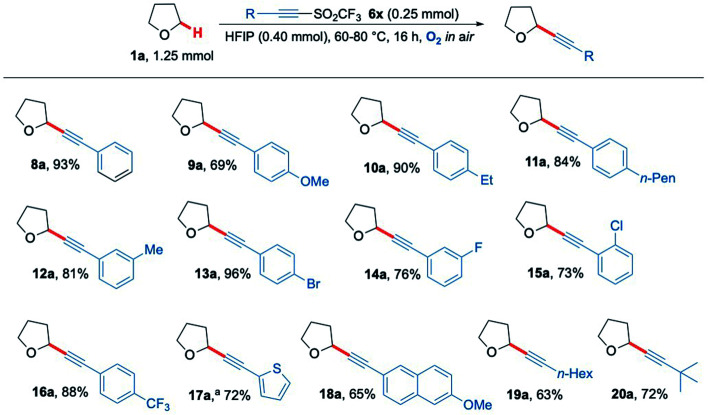
Substrate scope showing a range of acetylenic THF products 8a–20a formed *via* the aerobically generated THF radical from 1a. ^a^ No HFIP and 24 h reaction time.

Given the success in functionalising a range of ethereal-based species using aerobic C–H activation conditions, we next turned our attention to altering the functional groups on the acetylenic triflone. A diverse portfolio of acetylenic triflones 6b–m were synthesised,^71^ and reacted with THF under the optimised C–H functionalisation conditions (Scheme 5). A range of electron-rich aryl alkynes furnished the desired adducts 9a, 10a, 11a and 12a in excellent yields (Scheme 5). Aromatic halogen functionalities, which could serve as useful handles for further reactivity,^72^ were well tolerated, yielding the THF adducts 13a, 14a and 15a in 96%, 76% and 73% yield (respectively). Likewise, the medicinally relevant trifluoromethyl functional group 16a was compatible, providing the alkynyl THF scaffold in 88% yield. Other aromatic rings such as an electron-rich thiophene and a naphthalene ring system were also examined, successfully affording the corresponding acetylenic products 17a and 18a in 72% and 65% yields respectively. Aliphatic substituents on the alkyne also proved to be compatible with the radical chain process, including both straight-chain and branched groups, giving products 19a and 20a in good yield (Scheme 5). Overall, the methodology proved to be wide-ranging, giving access to privileged heterocyclic and acyclic alkyne scaffolds in a simple and efficient manner.

**Scheme 6:**
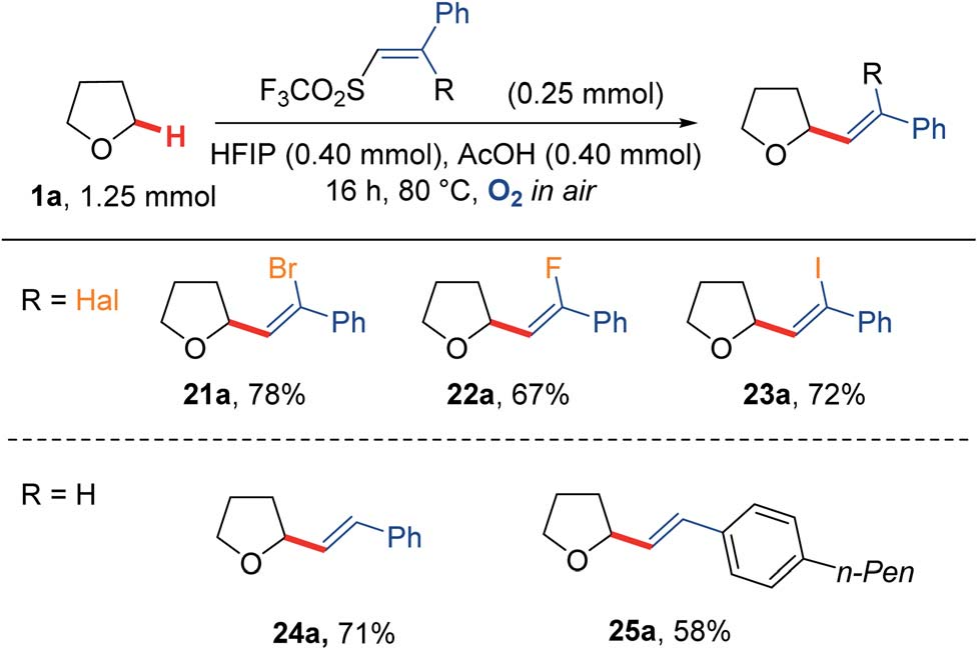
Reactivity of vinyl triflones under aerobic conditions to give the corresponding vinyl THF scaffolds 21a–25a.

We were also able to extend the reaction scope to vinyl triflone radical acceptors (Scheme 6).^73^ Using a mixture of AcOH/HFIP, which was found to improve the conversion for these reactions, good yields were observed for the formation of THF-alkene adducts 21a–25a.^74^ Use of a vinyl bromide led to a 78% isolated yield of adduct 21a. These conditions were applied to an analogous vinyl fluoride to afford THF-alkene 22a in 67% yield and a vinyl iodide analogue to give 23a in 72% yield. These reactions proceeded with retention of alkene geometry. The stereochemistry was assigned based on the three-bond *J*_HCCF_ coupling constants for the fluoro-olefin 22a (37 Hz), with the stereochemistry of the bromo-olefin 21a and iodo-olefin 23a assigned based on NOEs (see ESI† for further details). Application to simple 1,2-disubstituted vinyl triflones also yielded the respective products 24a and 25a, also proceeding *via* retention of vinyl triflone geometry to give the corresponding (*E*) isomers.

**Scheme 7:**
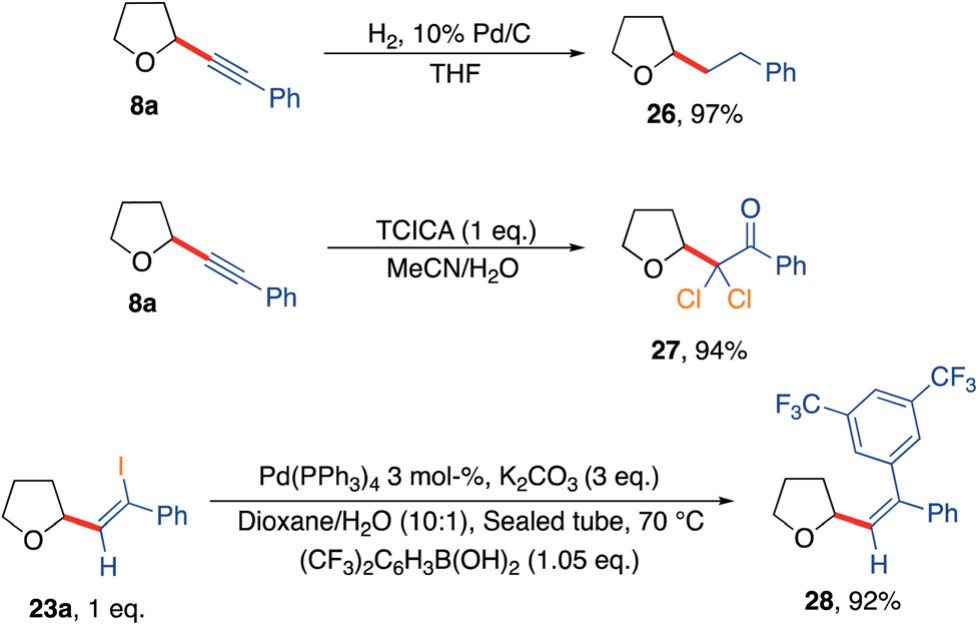
Further diversification of the synthesised products highlighting further utility of the developed aerobic C–H activation protocol.

Further reactions of some of the alkynlated products were also explored to demonstrate their utility as versatile building blocks (Scheme 7). For example, hydrogenation of product 8a afforded the corresponding straight chain alkane 26. Reaction of the acetylenic THF 8a with trichloroisocyanuric acid generated dichlorinated ketone 27 outlining the formation of a novel halogenated building block in a high yielding reaction (94%).^75^ Finally, a Suzuki coupling of THF-iodoalkene derivative 23a using 3,5-bis(trifluoromethyl)phenylboronic acid gave the corresponding coupled vinyl THF product 28 in an excellent yield of 92%.

**Scheme 8:**
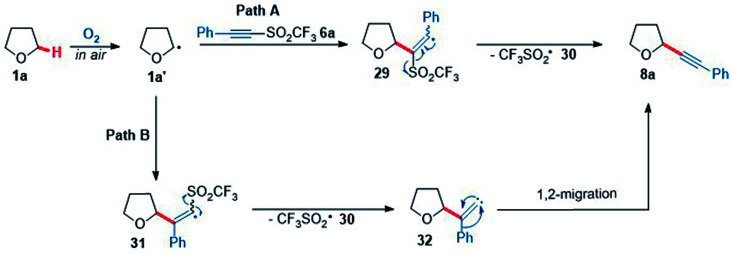
Two proposed mechanistic pathways for the reaction of THF 1a and phenyl acetylenic triflone 6a.

To better understand the reaction of THF with acetylenic triflone 6a, mechanistic studies were conducted. A proposed mechanism showing two possible routes for the formation of the alkynyl THF 8a is shown below (Scheme 8); it was hypothesised there would likely be two routes to form 8a. Pathway A proceeds by α-addition of the THF radical 1a′ to alkyne 6a to afford vinyl radical 29, followed by β-elimination to generate desired product 8a. This also leads to fragmentation of the trifluoromethylsulfonyl radical 30 to sulfur dioxide and the highly reactive trifluoromethyl radical, which can further propagate the chain reaction by abstracting an α-C–H proton of another molecule of THF 1a. Pathway B (Scheme 8) involves initial β-addition of the THF radical 1a′ to form α-trifluoromethylsulfonyl vinyl radical 31, which can then fragment to a vinylidene carbene species 32 and the trifluoromethylsulfonyl radical 30. Vinylidene carbene 32 can then undergo a Fritch-Buttenberg-Weichell type 1,2-rearrangement to give the acetylenic product 8a.^76^

**Scheme 9:**
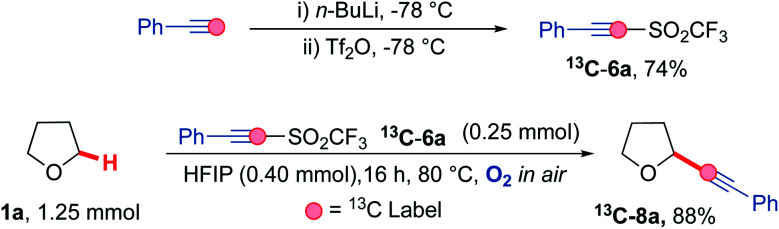
Mechanistic study involving ^13^C labelled phenyl acetylenic triflone ^13^C-6a under the optimised reaction conditions.

Carbon-13 labelling studies were used to distinguish between these two pathways *via* reaction of triflone ^13^C-6a with THF 1a (Scheme 9). Triflone ^13^C-6a was prepared from phenylacetylene-2-^13^C using the standard conditions used to form the triflone analogues. Selectively ^13^C labelled triflone ^13^C-6a was reacted with THF 1a under the reaction conditions to afford ^13^C-8a. The presence of the ^13^C label at the alpha position on the acetylenic product ^13^C-8a (highlighted in red) indicated that the reaction is highly likely to proceed *via* pathway A, where alpha addition of the THF radical 1a′ onto phenyl acetylenic triflone 6a affords the corresponding vinyl triflone radical 29. The presence of the ^13^C label was confirmed *via* an intense ^13^C peak in the acetylenic position (89.2 ppm, see ESI†) and the corresponding ^13^C couplings.

**Scheme 10:**
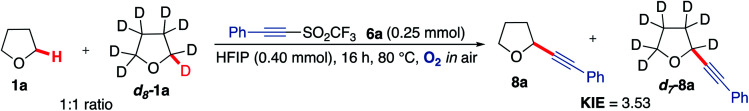
Calculation of a KIE *via* reaction of THF and THF-*d*_8_ with triflone 6a.

To gain further insight into the mechanism, a kinetic isotope effect (KIE) experiment was also performed. Treatment of phenyl acetylenic triflone with a mixture of THF and THF-*d*_8_ provided both products 8a and *d*_7_-8a (Scheme 10). A *K*_H_/*K*_D_ value of 3.53 was determined *via* liquid chromatography-mass spectrometry (LCMS) analysis (see ESI† for further details). This indicates that breakage of the initial α-C–H proton of THF is likely involved in the turnover limiting step, with a much faster reaction possible with the C–H bond than with a C–D bond.

**Scheme 11:**
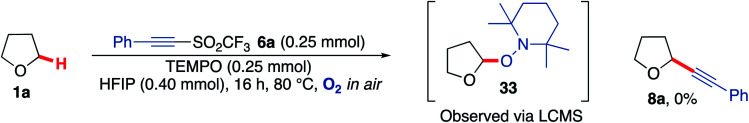
Radical trapping of THF 1a by TEMPO in the presence of triflone 6a, forming 33*in situ*, which was detected *via* LCMS.

The radical nature of the reaction was confirmed with the use of the radical trap TEMPO (2,2,6,6-(tetramethylpiperidin-1-yl)oxyl). THF 1a was reacted with acetylenic triflone 6a in the presence of an equivalent amount of TEMPO (0.25 mmol) at 80 °C. No product 8a was formed under these conditions (Scheme 11) and the presence of THF-TEMPO adduct 33 was detected *via* LCMS analysis (see ESI† for further details).

In conclusion, we have developed a method for direct aerobic C–H activation of a variety of heterocycles and ethers with subsequent C–C bond formation, by using alkenyl and alkynyl triflones as radical acceptors. This methodology represents one of the first examples of controlled metal/initiator free aerobic C–C bond formation from saturated heterocycles, and provides a breakthrough in aerobic C(sp^3^)–H activation methods for the formation of privileged C(sp^3^)–C(sp) and C(sp^3^)–C(sp^2^) bonds. A broad range of ethers, as well as some thioethers, were functionalised with a diverse range of alkynyl and alkenyl triflones with good functional group tolerance being observed. We provide mechanistic evidence for the aerobic radical pathway, involving regioselective addition of the radical to the α-position of the acetylenic triflone. Overall, we believe that the protocols we disclose in the manuscript provide a fundamental advancement in the C–H activation field, where the use of air represents a green, sustainable and freely accessible C–H bond oxidant to be utilised in synthesis.

## Data availability

All experimental and characterisation data in this article are available in the ESI.†

## Author contributions

N. A., T. D. S. and V. C. conceived and designed the project; N. A. performed the synthesis experiments and R. J. S. performed the LCMS experiments. All authors analysed the data; N. A., T. D. S. and V. C. co-wrote the paper.

## Conflicts of interest

There are no conflicts of interest to declare.

## Supplementary Material

SC-013-D2SC01626E-s001
