# Supplementary material for: Functionalisation of ethereal-based saturated heterocycles with concomitant aerobic C–H activation and C–C bond formation
Source: Chem Sci. 2022 Jun 27;13(29):8626–33. doi: 10.1039/d2sc01626e (PMC9337743; doi:10.1039/d2sc01626e)
Supplement: SC-013-D2SC01626E-s001 [file SC-013-D2SC01626E-s001.pdf]

Electronic Supplementary Material (ESI) for Chemical Science.
This journal is © The Royal Society of Chemistry 2022

General Experimental

Chemicals

All reagents were purchased from Merck or AlfaAesar and were used as received without further purification unless otherwise stated. All ethers were freshly distilled and degassed before usage in the reactions described below to remove radical inhibitors such as BHT.

Chromatography

All reactions were monitored by thin-layer chromatography (TLC) on pre-coated silica gel plates. Silica get plates were initially examined under short wave UV light and then developed using aqueous potassium permanganate stain. Flash column chromatography was carried out with pre-loaded GraceResolv™ flash cartridges on a Biotage® Isolera Spektra One flash chromatography system.

Spectroscopy

Quoted yields refer to chromatographically and spectroscopically pure compounds unless otherwise stated. ^{1}H NMR spectra were recorded at 400, 500 600 or 700 MHz and ^{13}C NMR at 126, 151 or 176 MHz on a Bruker Avance III 600 or Bruker Avance Neo 700 spectrometer. The chemical shifts (δ) for ^{1}H and ^{13}C are quoted relative to residual signals of the solvent on the parts per million (ppm) scale. In the case of multiple rotamers, only the major has been assigned. Coupling constants (*J* values) are reported in Hertz (Hz) and are reported as J_{H-H}. Signal multiplicities in ^{13}C NMR were determined using the distortionless enhancement by polarisation transfer (DEPT) spectral editing technique.

LCMS used for kinetic isotope effect experiments

LCMS was performed using a Waters Acquity uPLC connected to Waters Acquity Single Quad Detector (SQD). All samples were run with the following parameters. Column: Hypersil Gold C4, 1.9 µm, 2.1 µm × 50 µm. Wavelength: 254 nm. Mobile Phase: 50:50 Water (0.1% Formic Acid): MeCN (0.1% Formic Acid) Gradient over 4 min (to 5:95 Water (0.1% Formic Acid): MeCN (0.1% Formic Acid). Flow Rate: 0.6 mL/min. MS Mode: ES+. Scan Range: m/z = 150 – 300. Scan time: 0.25 s. Data obtained in continuum mode. The electrospray source of the MS was operated with a capillary voltage of 3.5 kV and a cone voltage of 50 V. Nitrogen was used

as the nebulizer and desolvation gas at a total flow of 600 L/h. Ion series were generated by integration of the total ion chromatogram (TIC) over the appropriate range.

Preparation of Reagents

(((Trifluoromethyl)sulfonyl)ethynyl)benzene – 6a

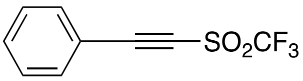

To a stirring solution of phenylacetylene (2.41 mL, 22.0 mmol, 1.1 eq.) in dry Et_2O (100 mL) was added *n*-BuLi (13.8 mL, 1.6 M in hexanes, 1.1 eq.) over 30 min at -78 °C under an argon atmosphere. After being stirred for 0.5 h at -78 °C, the corresponding lithium acetylide was slowly transferred *via* cannula transfer to a solution of triflic anhydride (3.36 mL, 20 mmol, 1.0 eq.) in dry Et_2O (50 mL) at -78 °C. The reaction was stirred at -78 °C for 30 min before being quenched with water. The aqueous layer was extracted with ether, and the combined organic layers were washed with brine, dried over $MgSO_4$, filtered, and concentrated under reduced pressure. The residue was purified by column chromatography (0-10% EtOAc/cyclohexane) to afford **6a** as a pale yellow oil (3.65g, 16 mmol) in 88% yield. ^{1}H NMR (500 MHz, $CDCl_3$) δ 7.70 (d, *J* = 7.5 Hz, 2 H), 7.64 (t, *J* = 7.5, Hz, 1 H), 7.47 (t, *J* = 7.2 Hz, 2 H); ^{13}C NMR (126 MHz, $CDCl_3$) δ 133.9 (CH), 133.5 (CH), 129.2 (CH), 119.1 (C, q, *J* = 323.1 Hz), 115.9 (C), 100.9 (C), 77.4 (C); IR (thin film): 3072, 2852, 2175, 2104, 1596, 1489, 1445 cm^{-1}. Known compound.[1] All other alkynyl triflones were prepared in an analogous manner using this procedure.

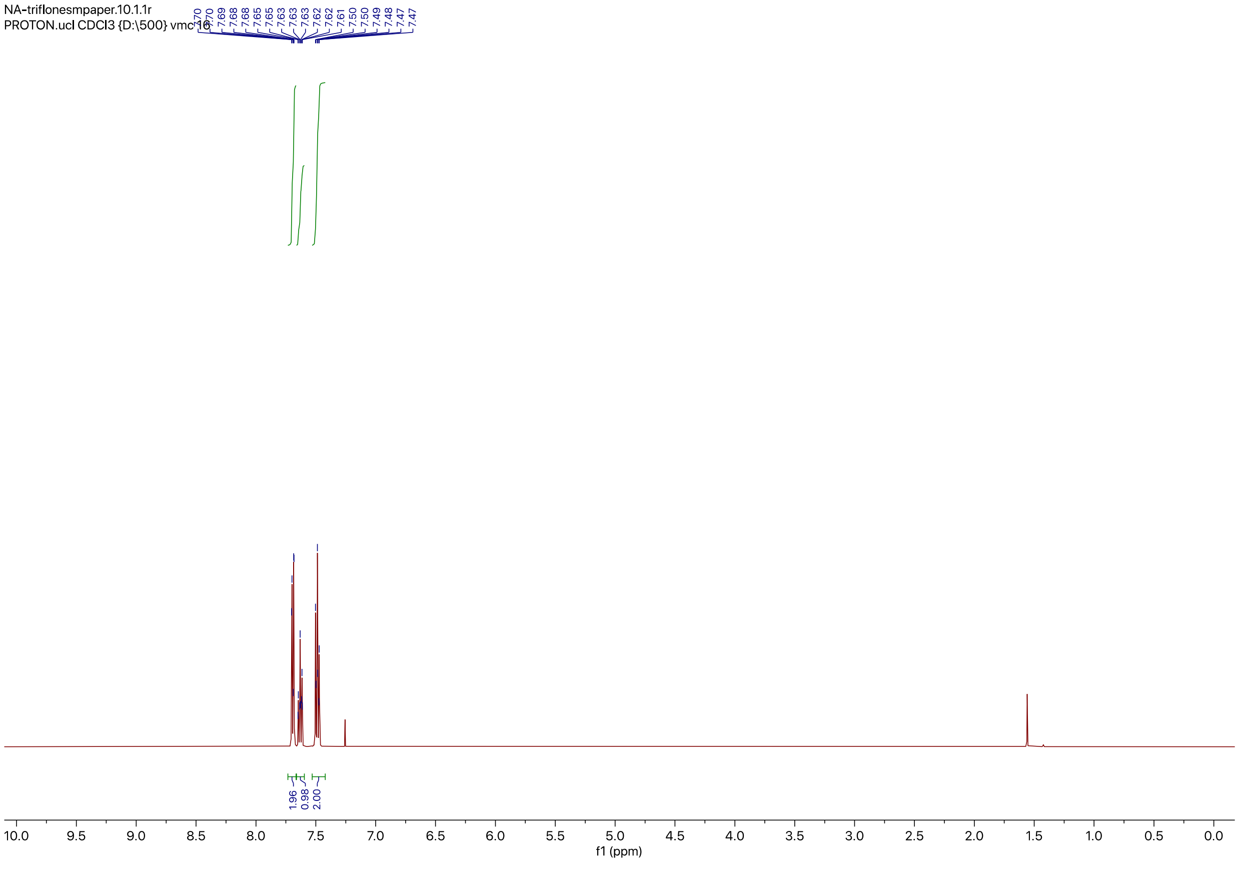

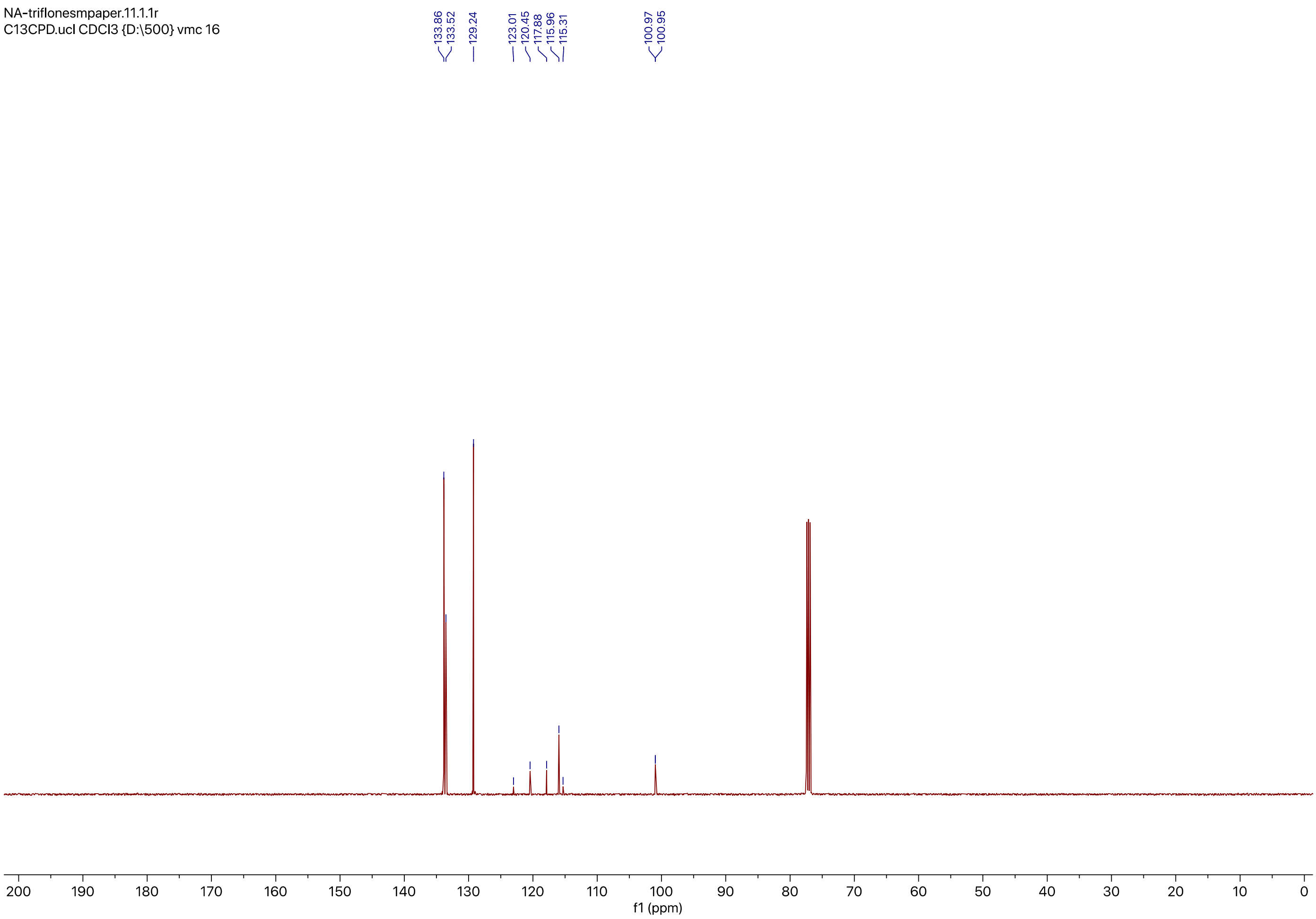
NA-triflonesmpaper.11.1.1r
C13CPD.ucl CDCl3 {D:\500} vmc 16
133.86
133.52
129.24
123.01
120.45
117.88
115.96
115.31
100.97
100.95
200
190
180
170
160
150
140
130
120
110
100
90
80
70
60
50
40
30
20
10
0
f1 (ppm)

2-oxo-2-phenylethyl 2-iodobenzoate – 7

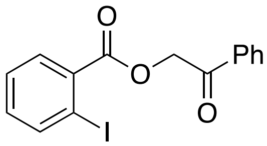

Rearranged product observed as a yellow oil from the reaction of THF **1a** with Ph-EBX **5** (Scheme 3 – Major side-product 50%). ^{1}H NMR (700 MHz, $CDCl_3$) δ 8.06 (dd, *J* = 7.8, 1.7 Hz, 1H), 8.02 (dd, *J* = 8.0, 1.0 Hz, 1H), 7.99 –7.94 (m, 2H), 7.71–7.59 (m, 1H), 7.54–7.48 (m, 2H), 7.45 (td, *J* = 7.6, 1.1 Hz, 1H), 7.19 (td, *J* = 7.7, 1.7 Hz, 1H), 5.60 (s, 2H). ^{13}C NMR (176 MHz, $CDCl_3$) δ 191.9 (C=O), 165.9 (C=O), 141.6 (CH), 134.3 (C), 134.2 (C), 134.1 (CH), 133.2 (CH), 131.8 (CH), 129.1 (CH), 128.2 (CH), 128.0 (CH), 94.6 (C-I), 66.9 (CH_2). IR (thin film): 2983, 2912, 1754, 1688, 1544 cm^{-1}. Known compound.[15]

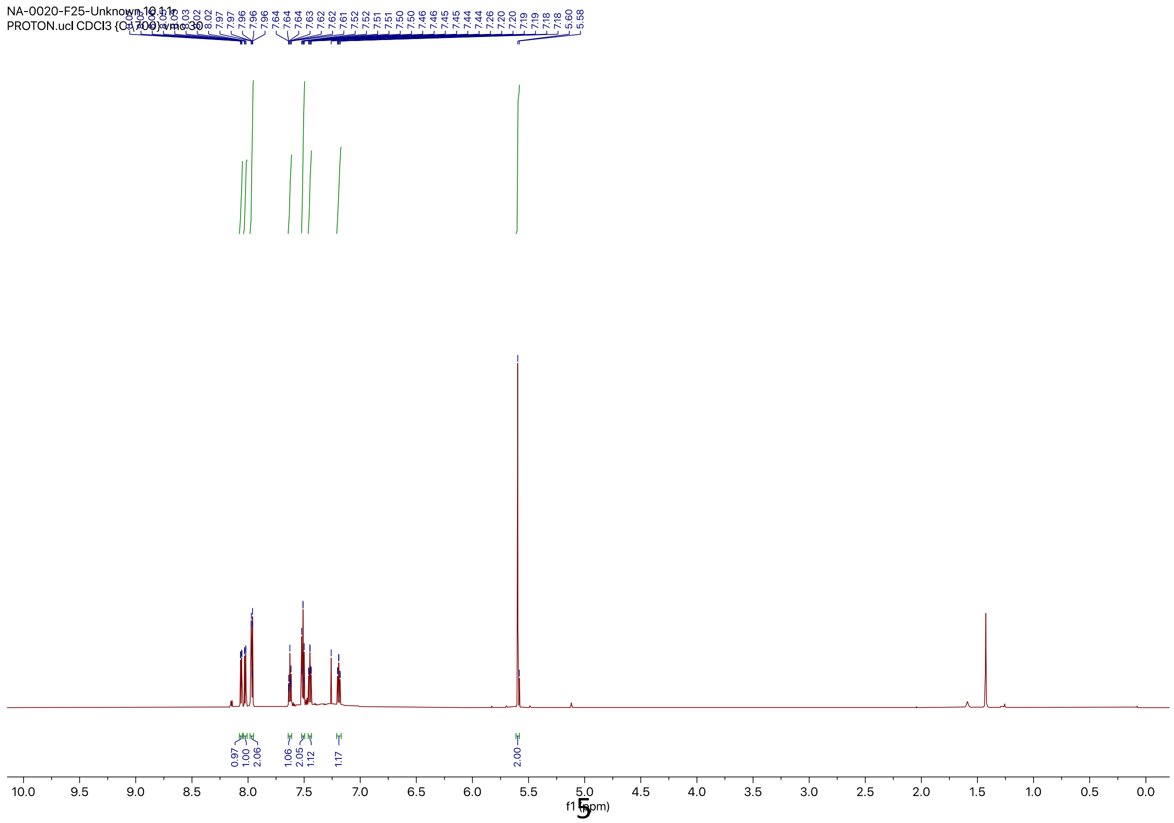

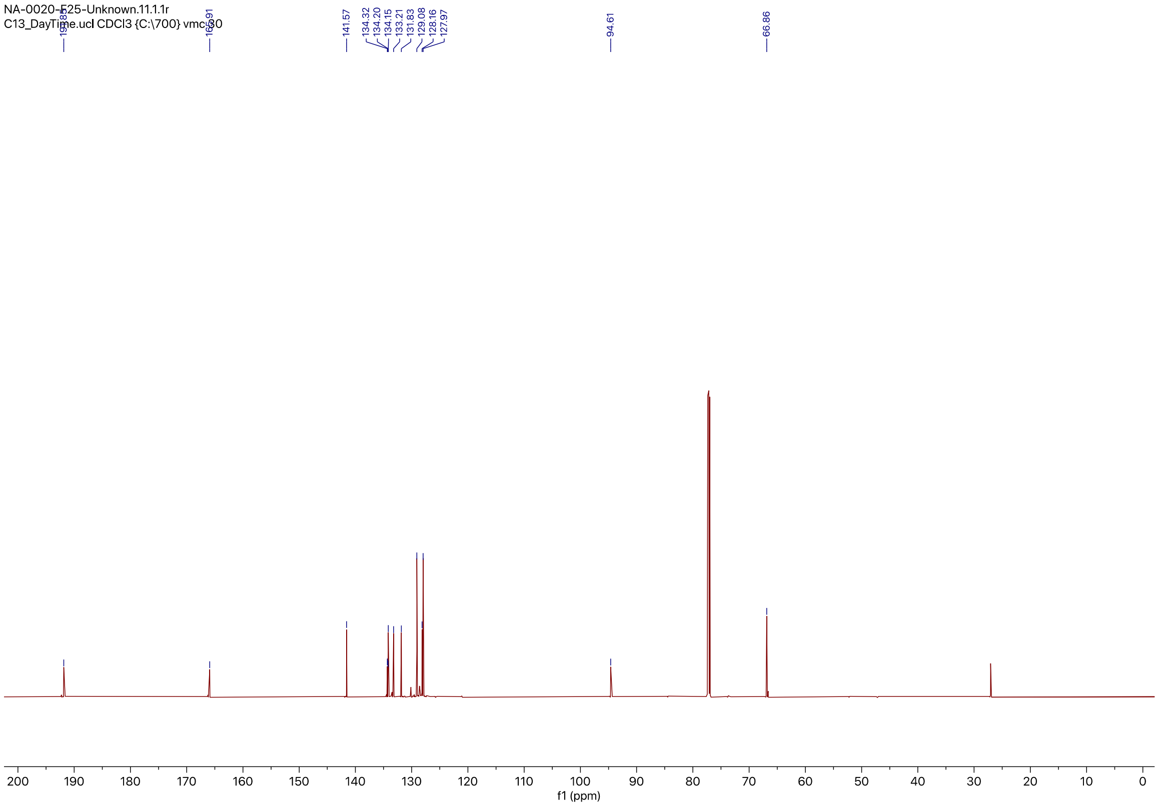
NA-0020-F25-Unknown.11.1.1r
C13_DayTime.ucl CDCl3 {C:\700} vmc-30
141.57
134.32
134.20
134.15
133.21
131.83
129.08
128.16
127.97
94.61
66.86
200 190 180 170 160 150 140 130 120 110 100 90 80 70 60 50 40 30 20 10 0
f1 (ppm)

General Procedure for aerobic C-H functionalisation of heterocycles – Method A (Alkynylation)

To a solution of heterocycle/ether (1.25 mmol, 5.0 eq.) in HFIP (0.40 mmol) was added (((trifluoromethyl)sulfonyl)ethynyl)benzene **6a** (0.25 mmol, 1.0 eq). The reaction mixture was stirred at 80 °C for 16 h and then poured over sat. aq. $NaHCO_3$ (5 mL). The organics were extracted with EtOAc (3 x 10 mL), dried over $MgSO_4$, filtered and the solvent removed under reduced pressure. The resultant crude residue was purified as described below.

2-(Phenylethynyl)tetrahydrofuran – 8a

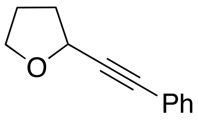

Following application of method A, purification by column chromatography (0-15% EtOAc/cyclohexane) afforded 2-(phenylethynyl)tetrahydrofuran **8a** as a clear oil (0.957g, 5.5 mmol, 89%). ^{1}H NMR ($CDCl_3$, 700 MHz): δ 7.43 (dd, 7.0, 3.0 Hz, 2H), 7.34–7.29 (m, 3H), 4.81 (dd, *J* = 7.2 Hz, 5.0 Hz, 1H), 4.08–4.01 (m, 1H), 3.86 (td, *J* = 8.0, 5.5 Hz, 1H), 2.29–2.20 (m, 1H), 2.17–2.07 (m, 2H), 2.00–1.91 (m, 1H); ^{13}C NMR (176 MHz, $CDCl_3$) δ 131.8 (CH), 128.3 (CH), 128.4 (CH), 123.0 (C), 89.2 (C), 84.6 (C), 68.7 (CH), 68.1 (CH_2), 33.6 (CH_2), 25.6 (CH_2); IR (thin film): 2929, 2209, 1602, 1558, 1521 cm^{-1}. Known compound.[2]

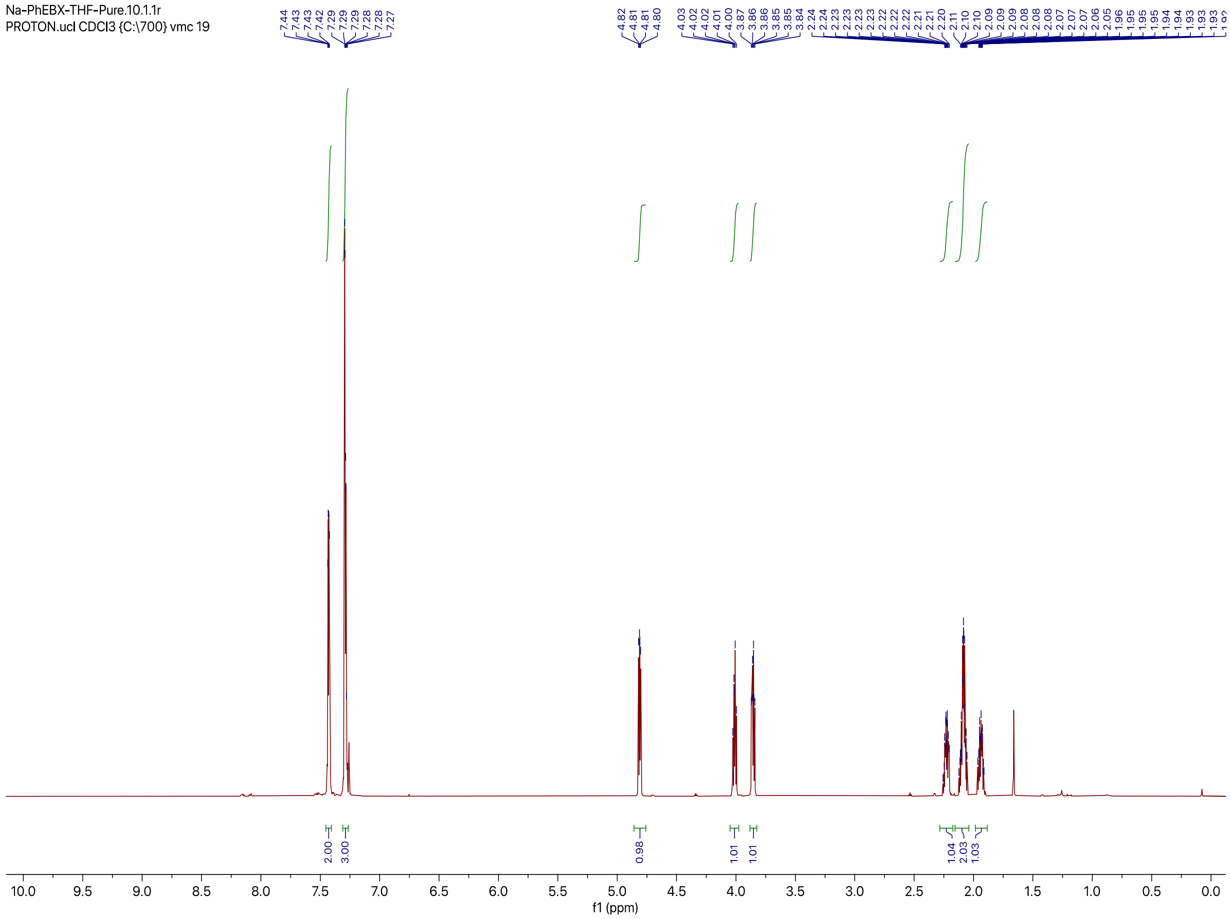

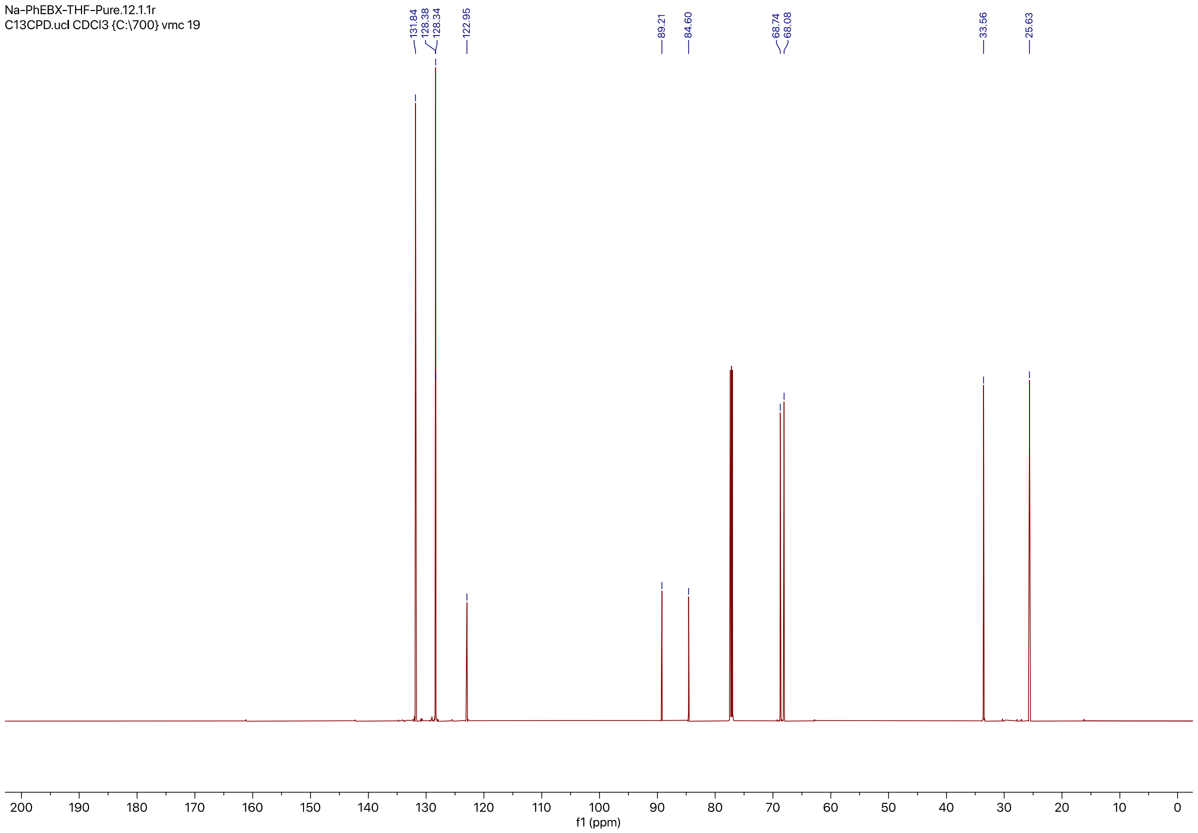
Na-PhEBX-THF-Pure.12.1.1r
C13CPD.ucl CDCl3 {C:\700} vmc 19
131.84
128.38
128.34
122.95
89.21
84.60
68.74
68.08
33.56
25.63
200
190
180
170
160
150
140
130
120
110
100
90
80
70
60
50
40
30
20
10
0
f1 (ppm)

2-Methyl-2-(phenylethynyl)tetrahydrofuran/ 2-methyl-5-(phenylethynyl)tetrahydrofuran – 8b, 8c-a + 8c-b

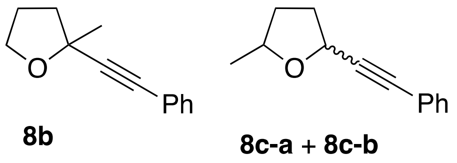

Following application of method A, purification by column chromatography (0-15% EtOAc/cyclohexane) afforded 2-methyl-2-(phenylethynyl)tetrahydrofuran **8b** and 2-methyl-5-(phenylethynyl)tetrahydrofuran **8c-a** and **8c-b** as a inseparable yellow oil (40 mg, 0.22 mmol, 86%). Assignment for each regioisomer/diastereomer is provided below. Product ratio: **1.7:1:0.5 8b**:**8c-a**:**8c-b** respectively. These ratios were calculated using NMR integrations of key protons such as the propargylic C-H.

8b: ^{1}H NMR (500 MHz, $CDCl_3$): δ 7.44–7.39 (m, 2H), 7.30–7.26 (m, 3H), 4.03–3.94 (m, 2H), 2.09–2.18 (m, 3H), 1.86 (dt, *J* = 12.4, 8.4 Hz, 1H), 1.68 (s, 3H). ^{13}C NMR (126 MHz, $CDCl_3$): δ 131.8 (CH), 128.3 (CH), 128.2 (CH), 123.2 (C) 92.5 (C), 82.9 (C), 76.5 (C), 67.8 (CH_2), 40.3 (CH_2), 27.8 (CH_3), 25.8 (CH_2).

8c-a: ^{1}H NMR (500 MHz, $CDCl_3$): δ 7.44–7.39 (m, 2H), 7.30–7.26 (m, 3H), 4.92 (dd, *J* = 7.2, 5.8 Hz, 1H), 4.33–4.24 (m, 1H), 2.36–2.27 (m, 2H), 2.17–2.13 (m, 1H), 1.51 (m, 1H), 1.28 (d, *J* = 8.1 Hz, 3H). ^{13}C NMR (126 MHz, $CDCl_3$): δ 131.8 (CH), 128.3 (CH), 128.2 (CH), 123.2 (C), 89.7 (C), 84.4 (C), 75.2 (CH), 68.5 (CH), 34.0 (CH_2), 33.2 (CH_2), 20.9 (CH_3).

8c-b: ^{1}H NMR (500 MHz, $CDCl_3$): δ 7.44–7.39 (m, 2H), 7.30–7.26 (m, 3H), 4.73 (dd, *J* = 7.2, 6.1 Hz, 1H), 4.08–4.03 (m, 1H), 2.27–2.23 (m, 2H), 2.17–2.12 (m, 1H), 1.76–1.66 (m, 1H), 1.35 (d, *J* = 6.1 Hz, 3H). ^{13}C NMR (126 MHz, $CDCl_3$): δ 131.8 (CH), 128.3 (CH), 128.2 (CH), 123.2 (C), 89.6 (C), 84.5 (C), 76.4 (CH), 68.7 (CH), 33.9 (CH_2), 33.1 (CH_2), 21.6 (CH_3).

IR (thin film): 3002, 2899, 2199, 1567, 1540, 1521 cm^{-1}. HRMS (ESI) calcd for $C_{14}H_{13}O$ $[M+H]^+$ 186.1039; observed 186.1032.

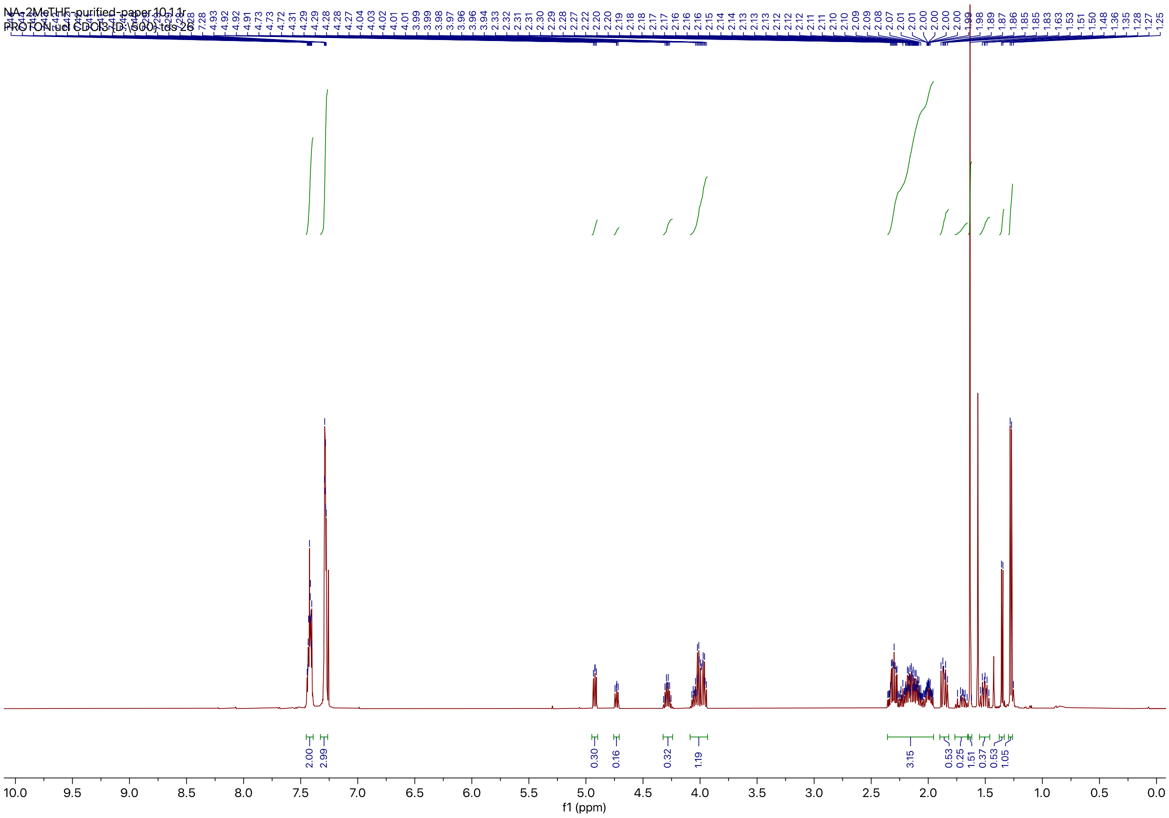
NA-2MeTHF-purified-paper.10.1.1r
PROTON.ucl CDCl3 {D:\500} tds 26
f1 (ppm)

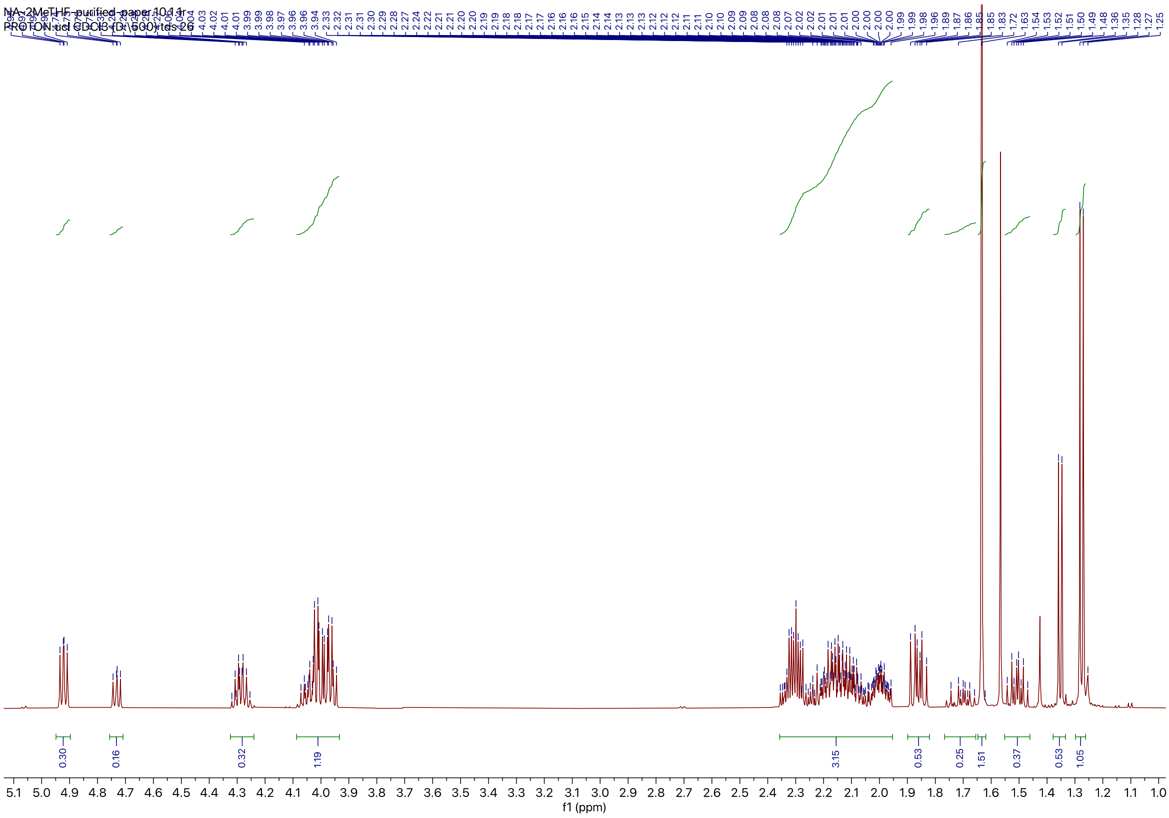
NA-2MeTHF-purified-paper.10.1.1r
PROTON.ucl CDCl3 {D:\500} tds 26
f1 (ppm)

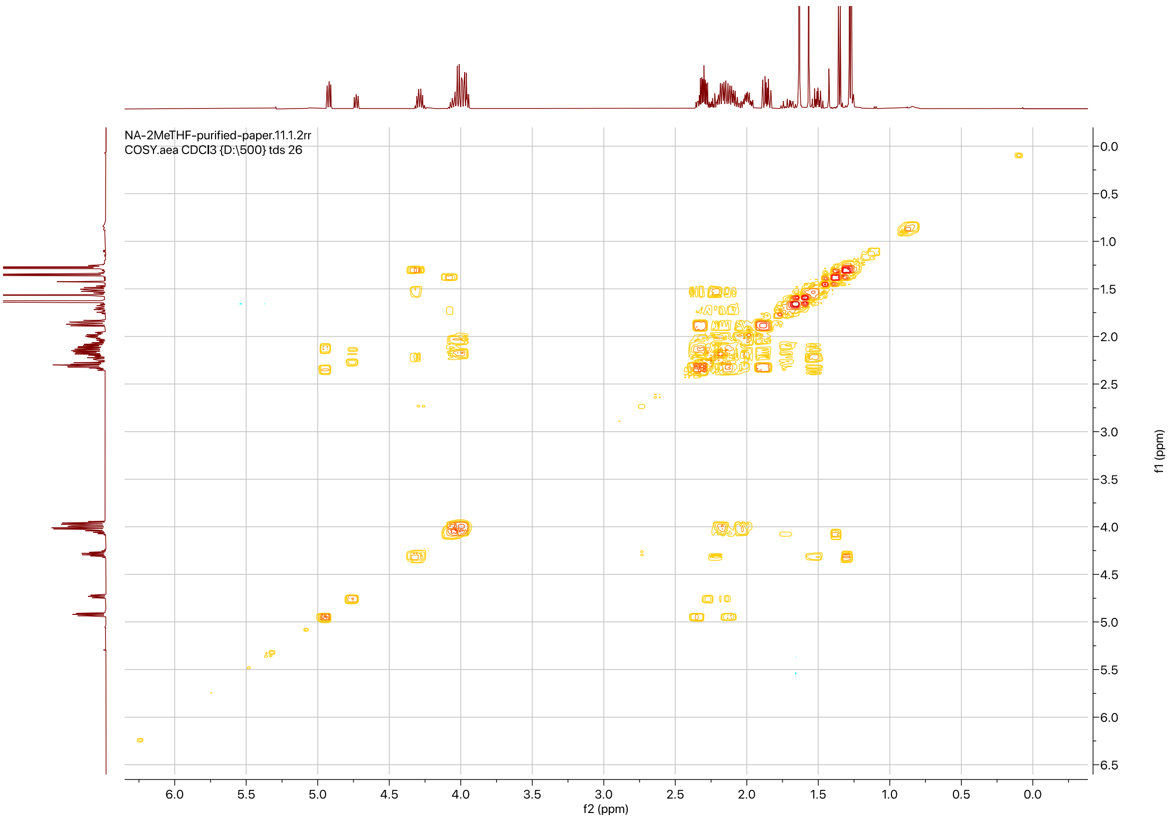
NA-2MeTHF-purified-paper.11.1.2rr
COSY.aea CDCl3 {D:\500} tds 26
f2 (ppm)
f1 (ppm)

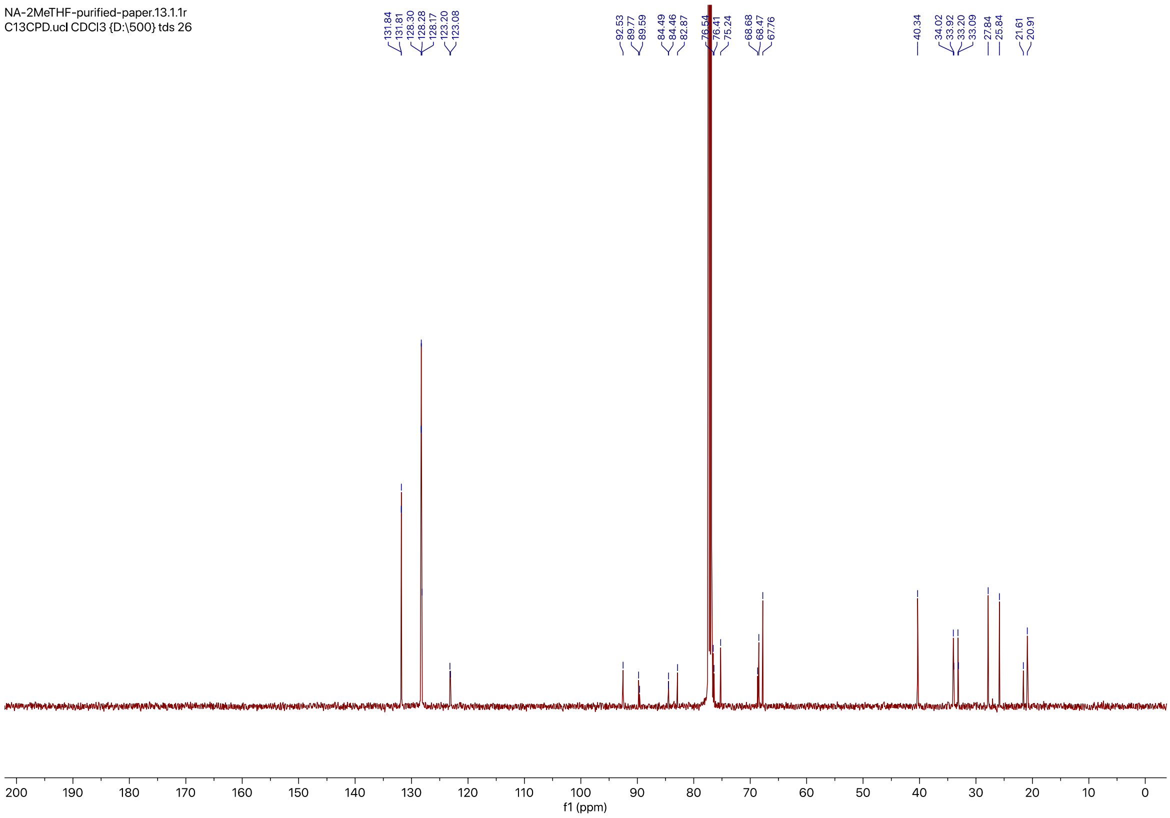
NA-2MeTHF-purified-paper.13.1.1r
C13CPD.ucl CDCl3 {D:\500} tds 26
131.84
131.81
128.30
128.28
128.17
123.20
123.08
92.53
89.77
89.59
84.49
84.46
82.87
76.54
76.41
75.24
68.68
68.47
67.76
40.34
34.02
33.92
33.20
33.09
27.84
25.84
21.61
20.91
f1 (ppm)

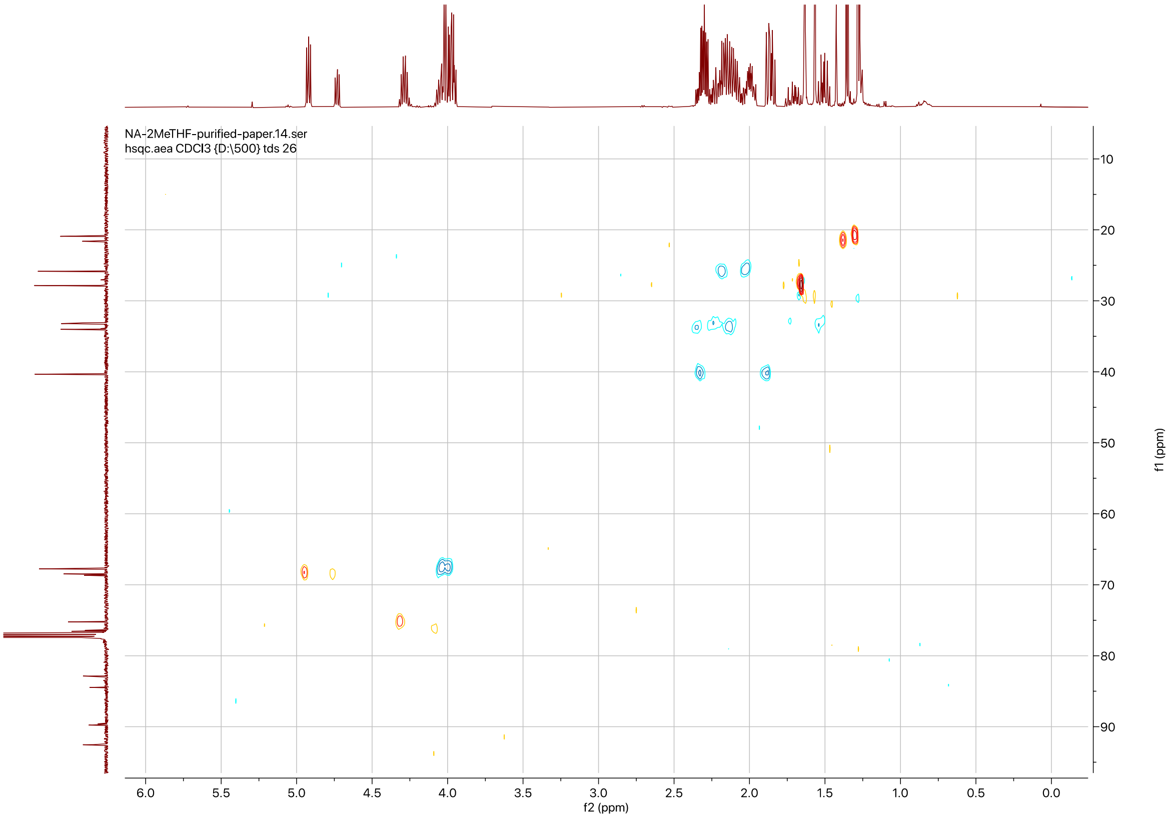
NA-2MeTHF-purified-paper.14.ser
hsqc.aea CDCl3 {D:\500} tds 26
f2 (ppm)
f1 (ppm)

2,5-Dimethyl-2-(phenylethynyl)tetrahydrofuran (unidentified mixture of 3:1 cis/trans isomers) – 8d

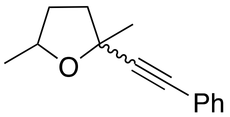

Following application of method A, purification by column chromatography (0-15% EtOAc/cyclohexane) afforded 2,5-dimethyl-2-(phenylethynyl)tetrahydrofuran **8d** (3:1 mixture of cis/trans isomers) as a yellow liquid (48 mg, 0.24 mmol, 97%). ^{1}H NMR (700 MHz, $CDCl_3$): δ 7.45–7.37 (m, 2H), 7.31–7.27 (m, 3H), 4.31 (dq, *J* = 12.7, 6.2 Hz, 0.70H), 4.22 (dq, *J* = 8.2, 6.1 Hz, 0.25H), 2.35 (ddd, *J* = 9.4, 6.4, 4.0 Hz, 0.25H), 2.29 (ddd, *J* = 12.2, 8.5, 6.0 Hz, 0.75H), 2.21 (ddt, *J* = 12.7, 8.5, 6.4 Hz, 0.75H), 2.16–2.11 (m, 0.25H), 1.94 (m, 1H), 1.91–1.82 (m, 0.25H), 1.63 (s, 2.25H), 1.61 (s, 0.75H), 1.57–1.52 (m, 0.75H), 1.36 (d, *J* = 6.1 Hz, 0.75H), 1.28 (d, *J* = 6.1 Hz, 2.25H). ^{13}C NMR (176 MHz, $CDCl_3$) δ 131.8 (CH), 131.7 (CH), 128.3 (CH), 128.1 (CH), 128.1 (CH), 123.4 (C), 123.2 (C), 93.8 (C), 93.1 (C), 82.7 (C), 82.5 (C), 76.6 (C), 76.3 (C), 75.3 (CH), 41.5 (CH_2), 40.4 (CH_2), 34.3 (CH_2), 33.1 (CH_2), 28.7 (CH_3), 28.4 (CH_3), 22.4 (CH_3), 21.4 (CH_3). IR (thin film): 3011, 2921, 2215, 1600, 1540, 1512 cm^{-1}. HRMS (ESI) calcd for $C_{14}H_{16}O$ $[M+H]^+$ 201.1274; observed 201.1268.

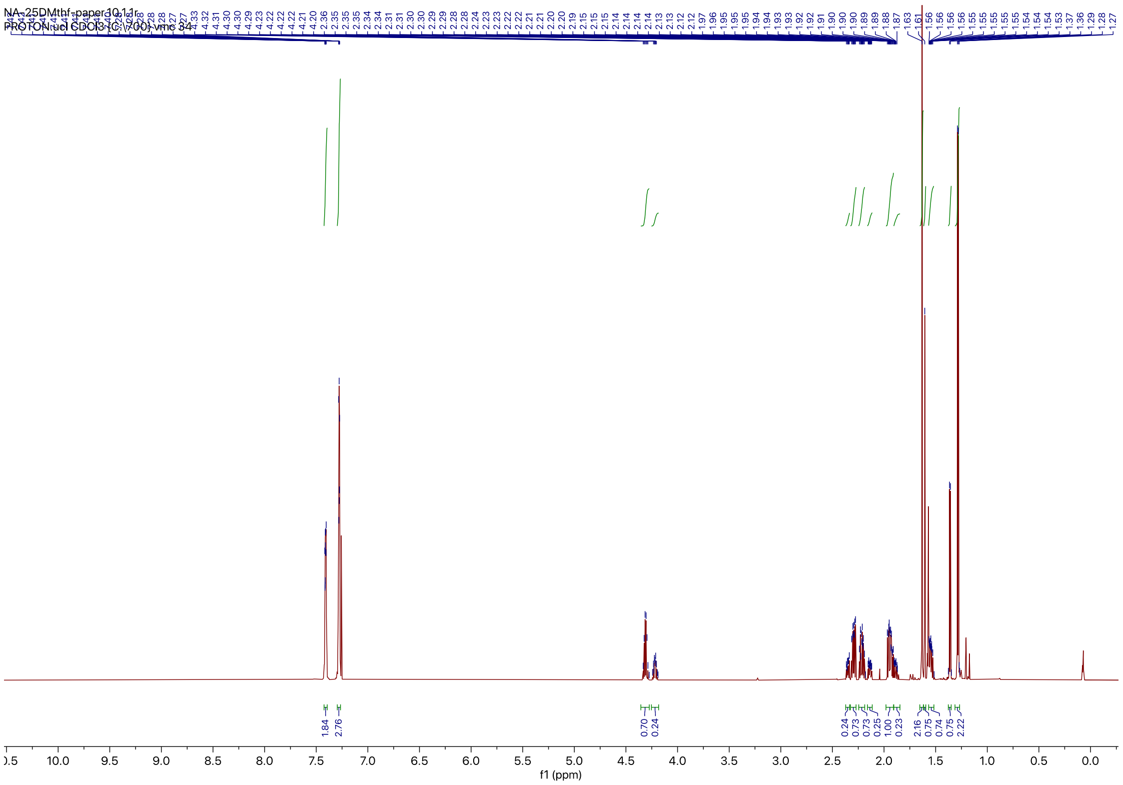

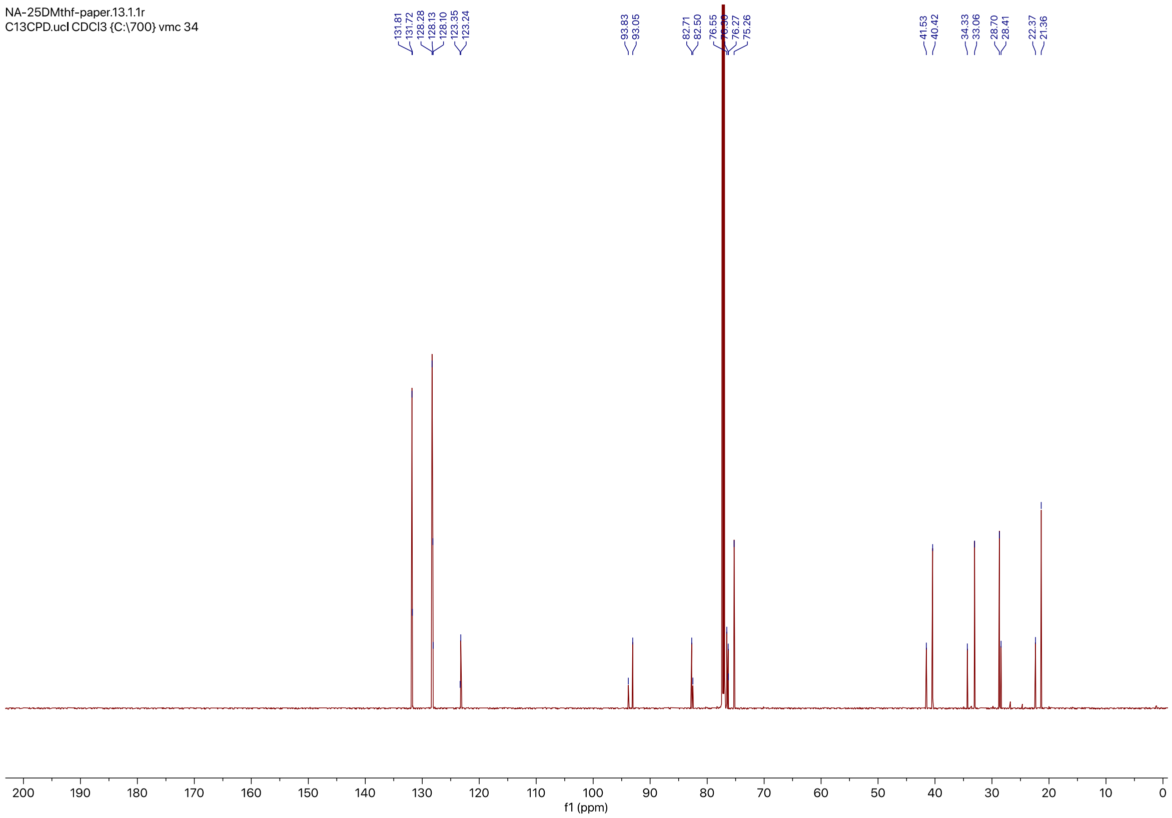
NA-25DMthf-paper.13.1.1r
C13CPD.ucl CDCl3 {C:\700} vmc 34
131.81
131.72
128.28
128.13
128.10
123.35
123.24
93.83
93.05
82.71
82.50
76.55
76.27
75.26
41.53
40.42
34.33
33.06
28.70
28.41
22.37
21.36
f1 (ppm)

5-(Phenylethynyl)dihydrofuran-3(2*H*)-one – 8e

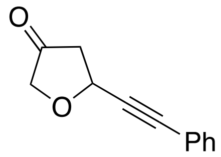

Following application of method A, purification by column chromatography (0-15% EtOAc/cyclohexane) afforded 5-(phenylethynyl)dihydrofuran-3(2*H*)-one **8e** as a clear oil (40 mg, 0.21 mmol, 84%). ^{1}H NMR ($CDCl_3$, 400 MHz): δ ^{1}H NMR (500 MHz, $CDCl_3$): δ 7.44–7.40 (m, 2H), 7.38–7.29 (m, 3H), 5.30 (dd, *J* = 7.6, 5.2 Hz, 1H), 4.16 (d, *J* = 16.8 Hz, 1H), 3.98 (d, *J* = 16.8 Hz, 1H), 2.85 (dd, *J* = 17.8, 7.7 Hz, 1H), 2.68 (dd, *J* = 17.8, 5.2 Hz, 1H). ^{13}C NMR (126 MHz, $CDCl_3$): δ 213.0 (C=O), 132.0 (CH), 129.1 (CH), 128.5 (CH), 121.9 (C), 87.1 (C), 86.2 (C), 69.8 (CH_2), 67.8 (CH), 43.9 (CH_2).); IR (thin film): 2911, 2901, 2206, 1724, 1613, 1558, 1543 cm^{-1}. HRMS (ESI) calcd for $C_{12}H_{10}O_2$ $[M+H]^+$ 187.0675; observed 187.0643.

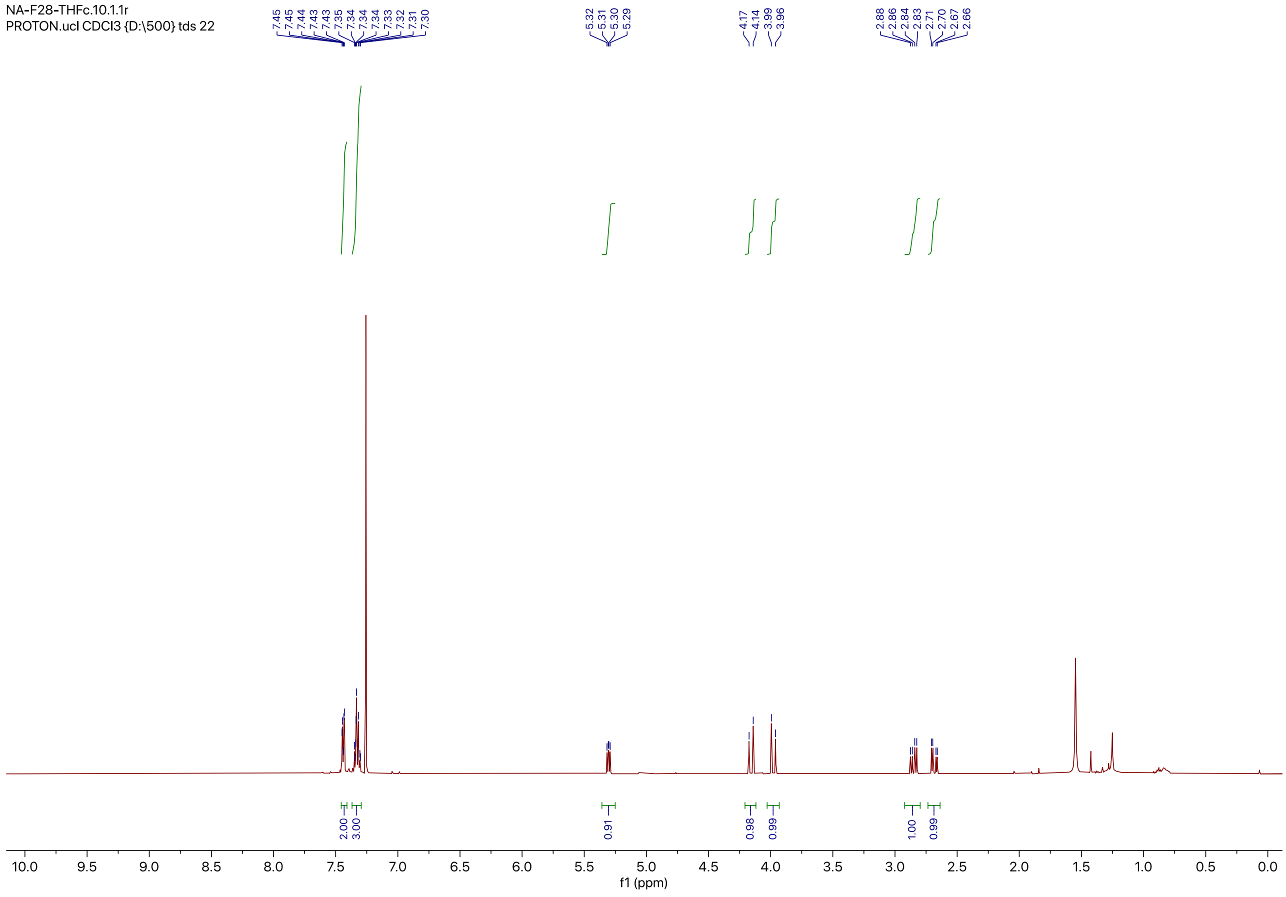

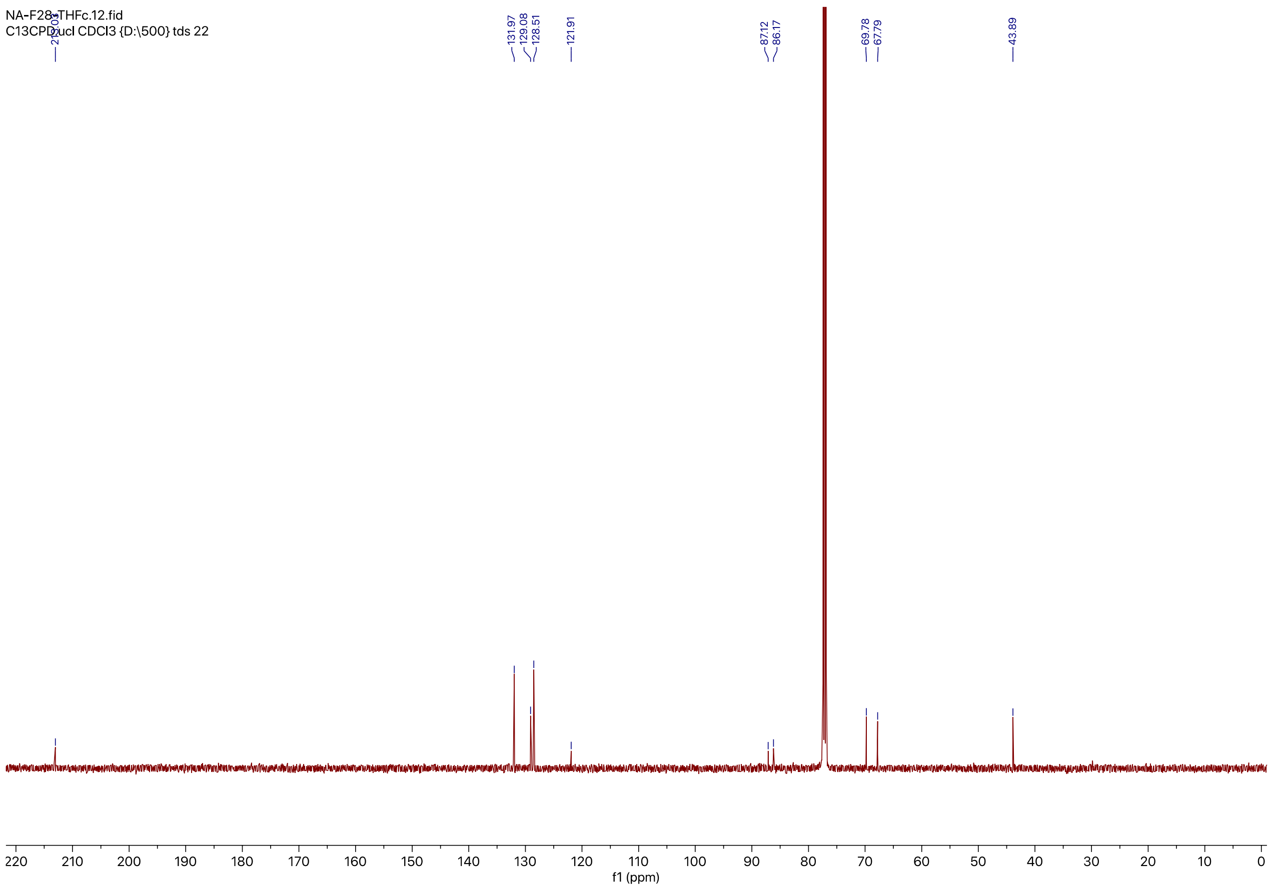
NA-F28-THFc.12.fid
C13CPDzucl CDCl3 {D:\500} tds 22
213.01
131.97
129.08
128.51
121.91
87.12
86.17
69.78
67.79
43.89
220 210 200 190 180 170 160 150 140 130 120 110 100 90 80 70 60 50 40 30 20 10 0
f1 (ppm)

2-Methyl-5-(phenylethynyl)dihydrofuran-3(2*H*)-one – 8f

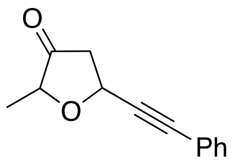

Following application of method A, purification by column chromatography (0-15% EtOAc/cyclohexane) afforded 2-(phenylethynyl)-1,4-dioxane **8f** as a yellow oil (39 mg, 0.20 mmol, 78%). ^{1}H NMR ($CDCl_3$, 400 MHz): δ 7.42 (dd, *J* = 7.5, 2.0 Hz, 2H), 7.34–7.31 (m, 3H), 5.35 (dd, *J* = 8.4, 3.0 Hz, 1H), 4.20 (q, *J* = 6.9 Hz, 1H), 2.87 (dd, *J* = 17.9, 8.4 Hz, 1H), 2.70 (dd, *J* = 17.9, 3.0 Hz, 1H), 1.34 (d, *J* = 7.0 Hz, 3H); ^{13}C NMR (101 MHz, $CDCl_3$) δ 214.7 (C=O), 131.9 (CH), 129.1 (CH), 128.4 (CH), 121.9 (C), 86.9 (C), 86.4 (C), 73.3 (CH), 65.2 (CH), 43.3 (CH_2), 15.6 (CH_3); IR (thin film): 2915, 2899, 2211, 1723, 1613, 1560, 1543 cm^{-1}. HRMS (ESI) calcd for $C_{13}H_{12}O_2$ $[M+H]^+$ 201.0910; observed 201.0903.

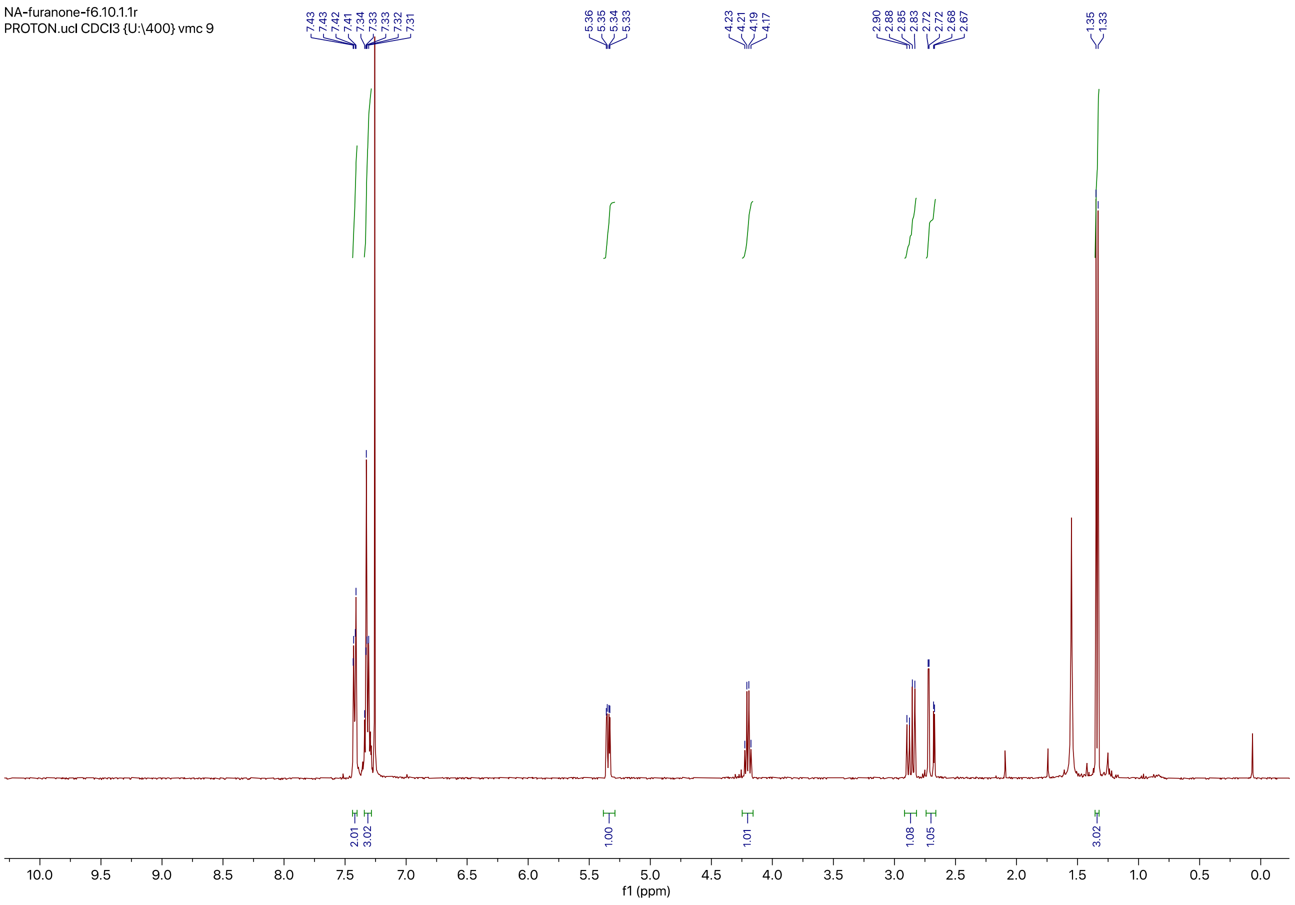

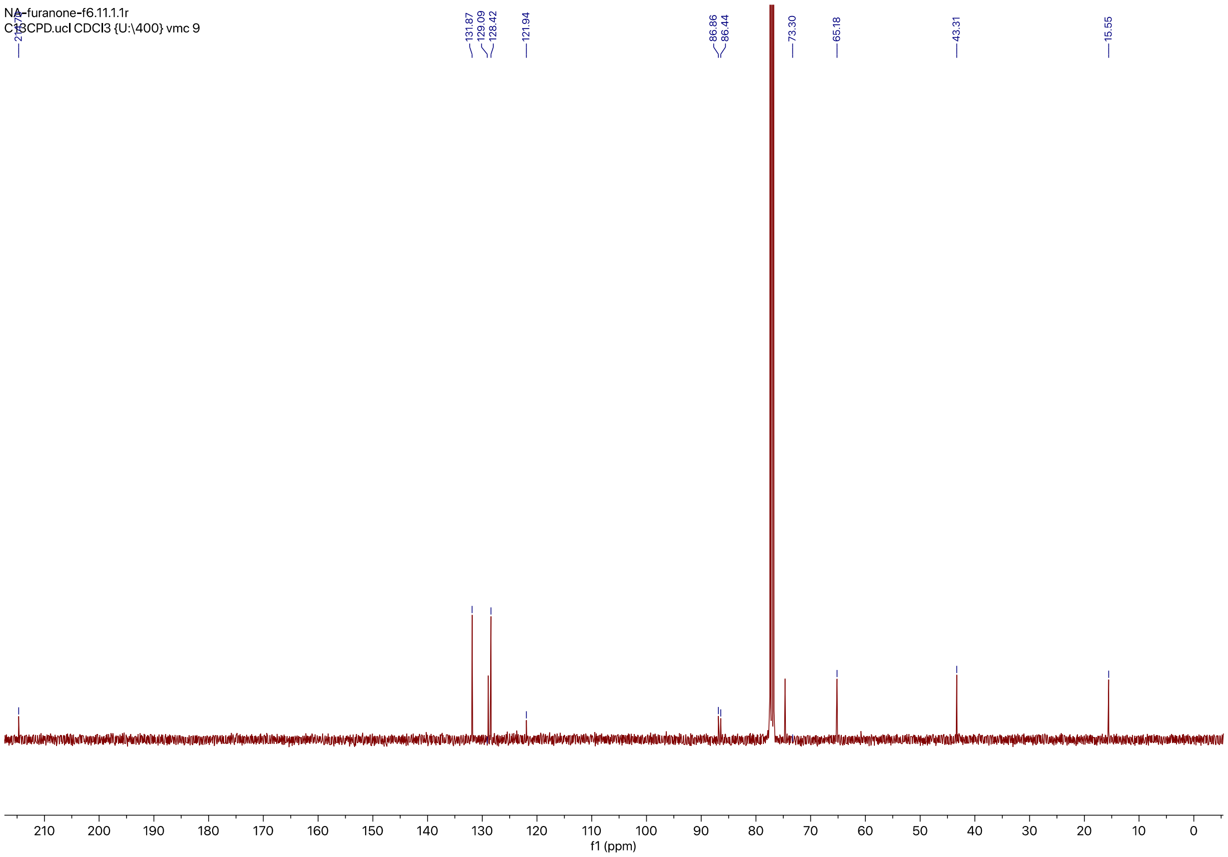
NA-furanone-f6.11.1.1r
C13CPD.ucl CDCl3 {U:\400} vmc 9
214.70
131.87
129.09
128.42
121.94
86.86
86.44
73.30
65.18
43.31
15.55
210
200
190
180
170
160
150
140
130
120
110
100
90
80
70
60
50
40
30
20
10
0
f1 (ppm)

((2*R*)-4-Oxo-5-(phenylethynyl)tetrahydrofuran-2-yl)methyl acetate and (2-(phenylethynyl)tetrahydrofuran-2-yl)methyl acetate – 8g-a, 8g-b and 8h.

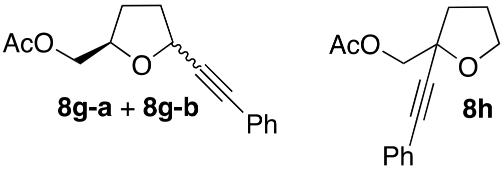

Following application of method A, purification by column chromatography (0-15% EtOAc/cyclohexane) afforded ((2*R*)-4-oxo-5-(phenylethynyl)tetrahydrofuran-2-yl)methyl acetate **8g-a + 8g-b** and (2-(phenylethynyl)tetrahydrofuran-2-yl)methyl acetate **8h** as an inseperable yellow oil (39 mg, 0.15 mmol, 61%). Assignment for each regioisomer/diastereomer is provided below. Product ratio is **1:0.35:0.85 8g-a**:**8g-b**:**8h** respectively. These ratios were calculated using NMR integrations of key protons such as the propargylic C-H.

8g-a: ^{1}H NMR ($CDCl_3$, 700 MHz): δ 7.47–7.38 (m, 2H), 7.34–7.27 (m, 3H), 4.95 (t, *J* = 6.2 Hz, 1H), 4.42 (qd, *J* = 7.0, 3.6 Hz, 1H), 4.18 (dd, *J* = 11.6, 3.6 Hz, 1H), 4.01–3.97 (m, 1H), 2.17–2.14 (m, 1H), 2.09 (s, 3H), 2.00-1.91 (m, 2H), 1.75–1.70 (m, 1H). ^{13}C NMR (176 MHz, $CDCl_3$): δ 171.2 (br C=O), 131.9 (CH), 128.5 (CH), 128.4 (CH), 122.8 (C), 85.0 (C), 88.5 (C), 76.6 (CH), 69.4 (CH), 27.9 (CH_2), 25.8 (CH_2), 21.06 (CH_3).

8g-b: ^{1}H NMR ($CDCl_3$, 700 MHz): δ 7.47–7.38 (m, 2H), 7.34–7.27 (m, 3H), 4.86 (dd, *J* = 7.1, 5.1 Hz, 1H), 4.29 (dd, *J* = 11.4, 3.7 Hz, 1H), 4.12 (dd, *J* = 11.4, 6.8 Hz, 1H), 4.08–4.04 (m, 1H), 2.25–2.00 (m, 3H), 2.08 (s, 3H), 1.91 (dq, *J* = 12.6, 8.2 Hz, 1H). ^{13}C NMR (176 MHz, $CDCl_3$): δ 171.2 (br C=O), 131.8 (CH), 128.6 (CH), 128.4 (CH), 122.8 (C), 88.9 (C), 84.9 (C), 77.7 (CH), 69.4 (CH), 67.0 (CH_2), 29.8 (CH_2), 28.1 (CH_2), 22.8 (CH_3).

8h: ^{1}H NMR ($CDCl_3$, 700 MHz): δ 7.47–7.38 (m, 2H), 7.34–7.27 (m, 3H), 4.38 (d, *J* = 11.3 Hz, 1H), 4.22 (d, d, *J* = 11.3 Hz, 1H), 4.08–4.04 (m, 2H), 2.28–2.20 (m, 3H), 2.13 (s, 3H), 2.11–2.05 (m. 1H). ^{13}C NMR (176 MHz, $CDCl_3$): δ 170.9 (C=O), 131.9 (CH), 128.6 (CH), 128.4 (CH), 122.6 (C), 88.8 (C), 84.9 (C), 77.2 (C), 68.2 (C), 66.4 (C), 36.0 (CH_2), 33.5 (CH_2), 21.1 (CH_3).

IR (thin film): 2901, 2211, 1745, 1600, 1565, 1547 cm^{-1}. HRMS (ESI) calcd for $C_{15}H_{16}O_3$ $[M+H]^+$ 245.1172; observed 245.1161.

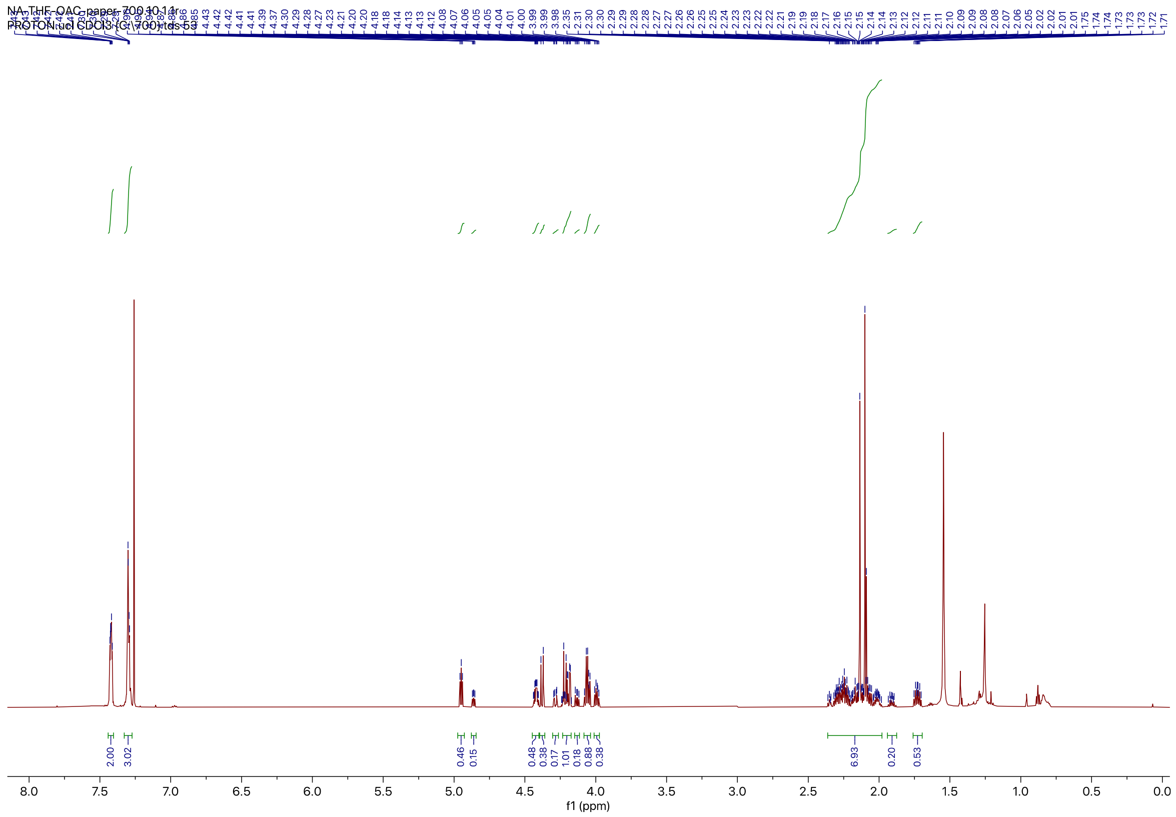

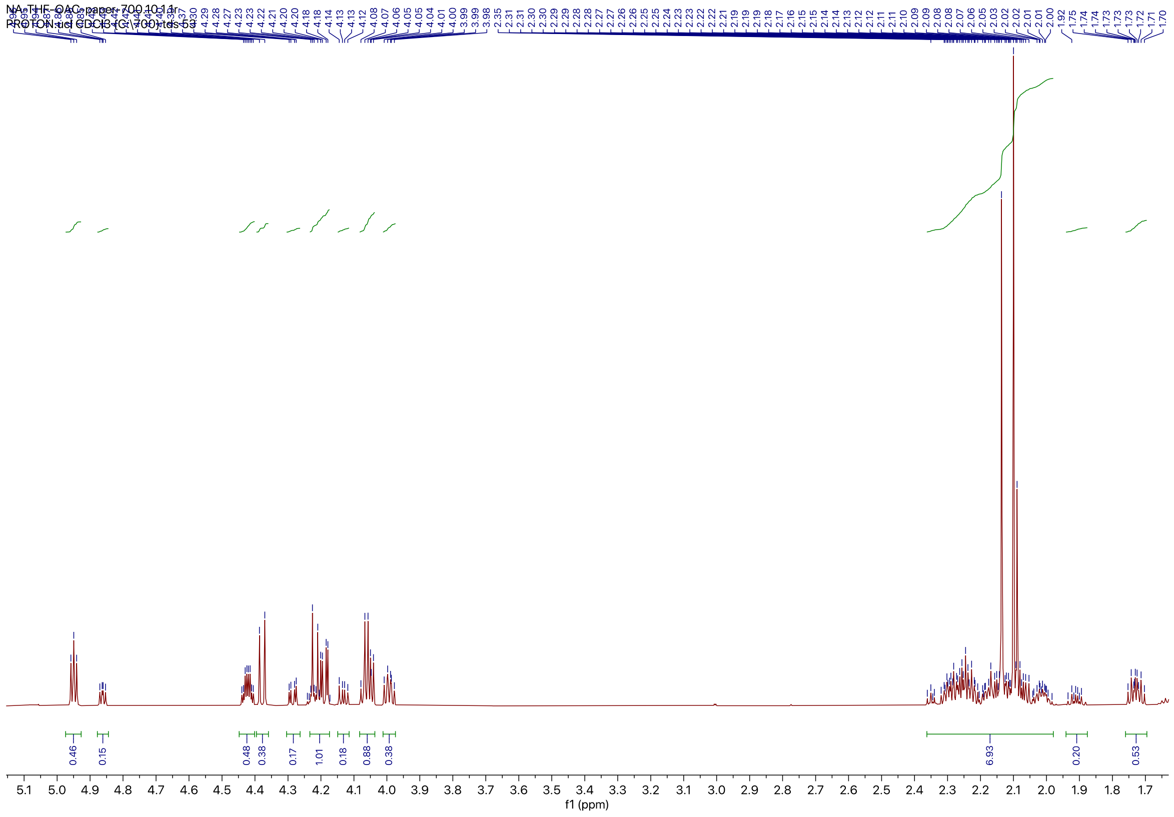

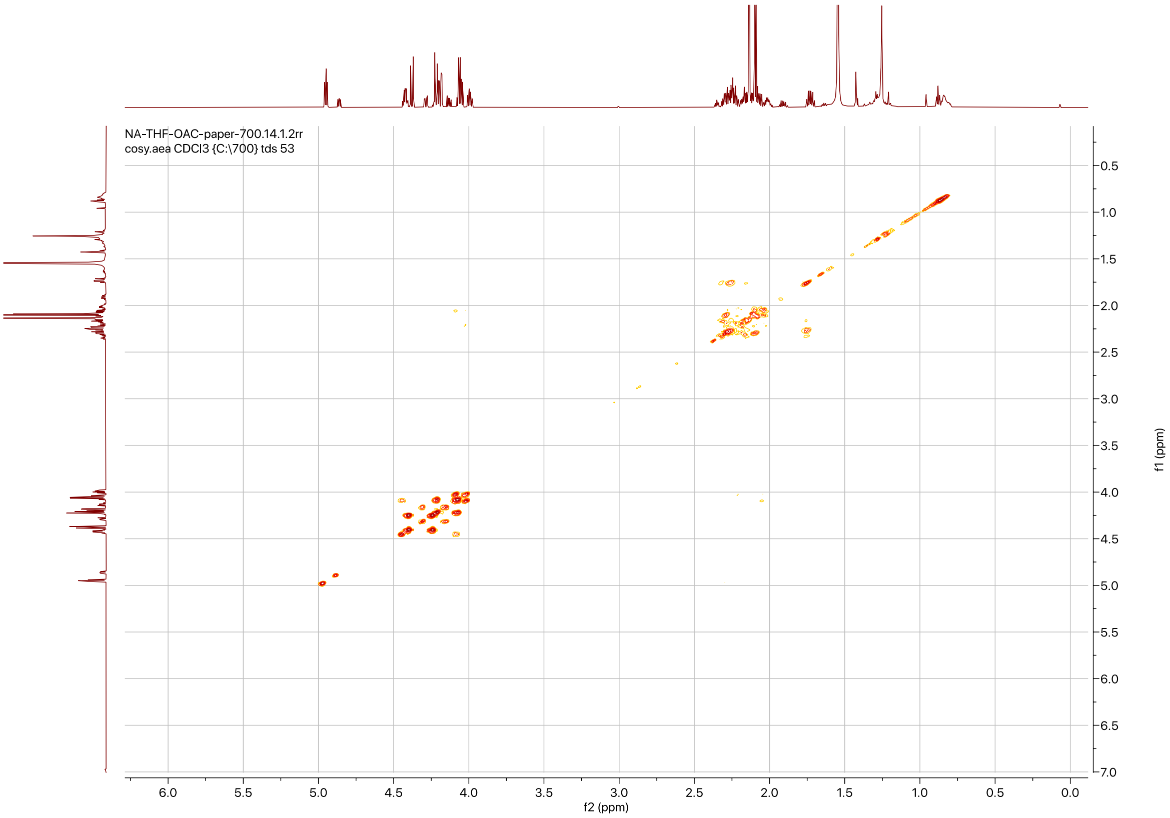
NA-THF-OAC-paper-700.14.1.2rr
cosy.aea CDCl3 {C:\700} tds 53
f2 (ppm)
f1 (ppm)

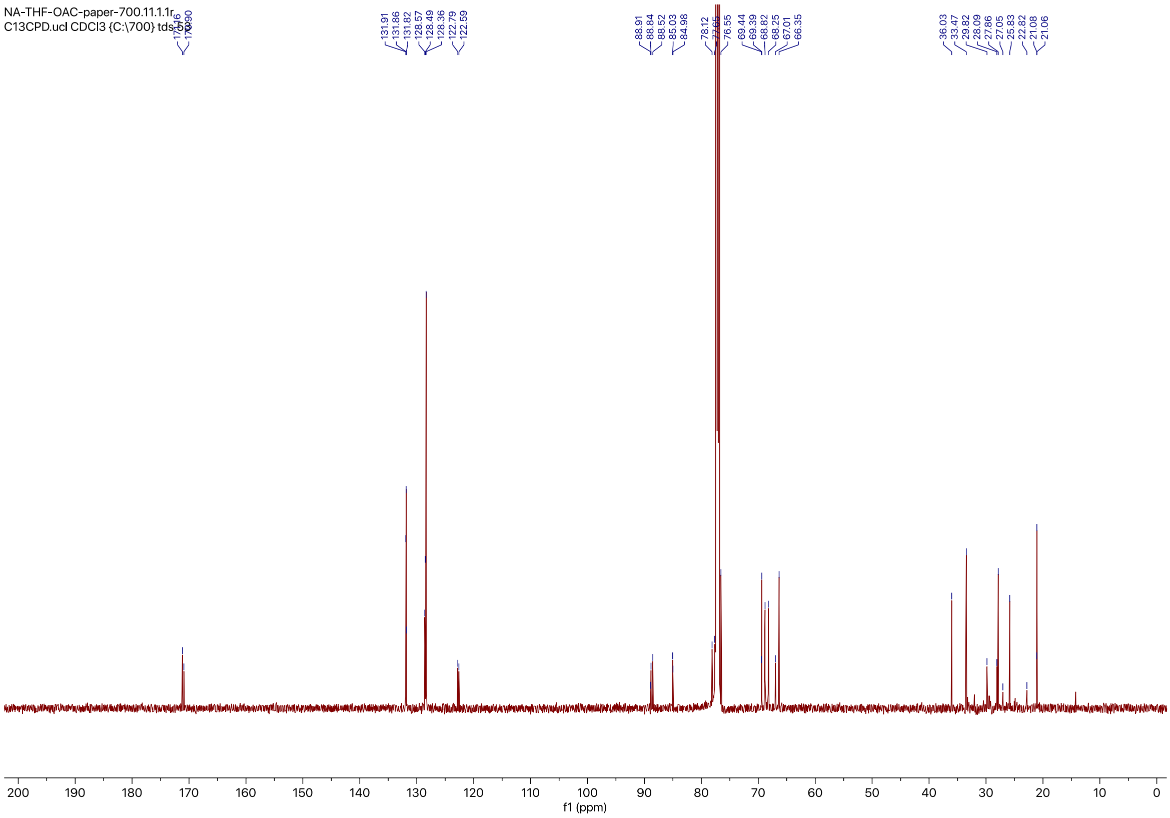
NA-THF-OAC-paper-700.11.1.1r
C13CPD.ucl CDCl3 {C:\700} tds 53
171.16
170.90
131.91
131.86
131.82
128.57
128.49
128.36
122.79
122.59
88.91
88.84
88.52
85.03
84.98
78.12
77.65
76.55
69.44
69.39
68.82
68.25
67.01
66.35
36.03
33.47
29.82
28.09
27.86
27.05
25.83
22.82
21.08
21.06
f1 (ppm)

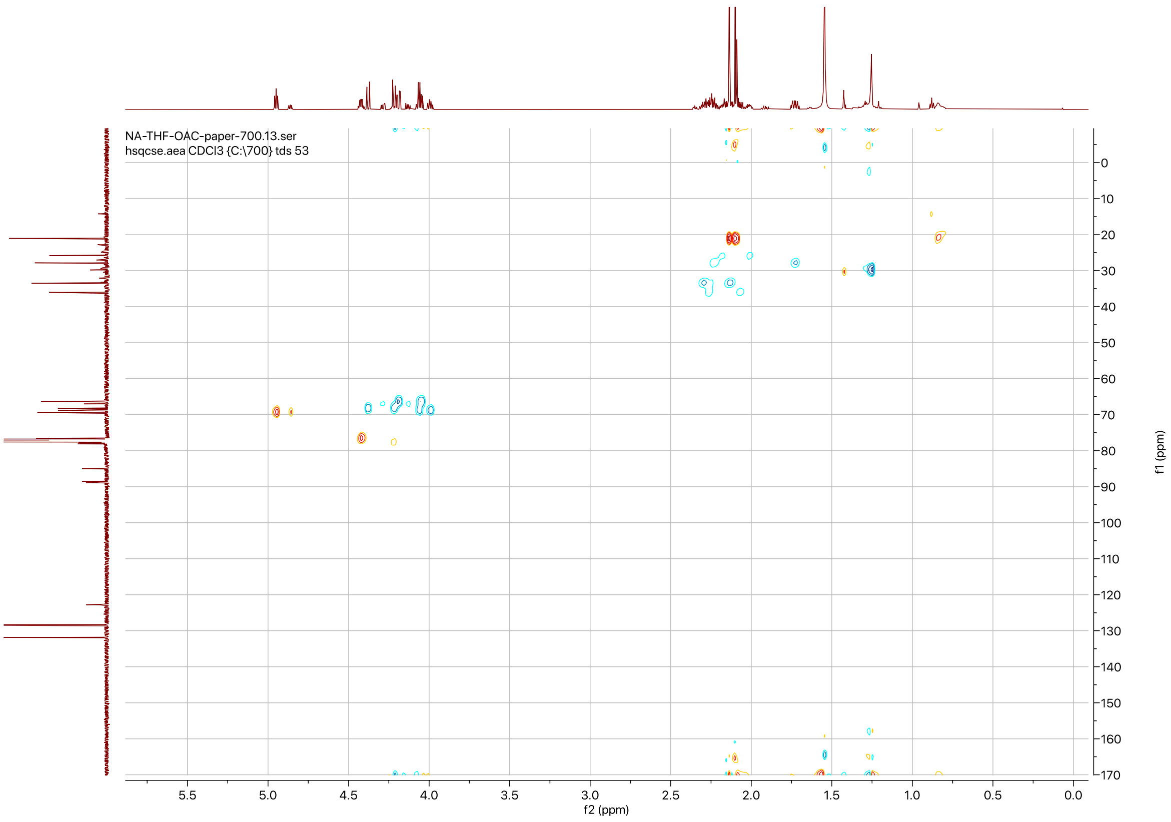
NA-THF-OAC-paper-700.13.ser
hsqcse.aea CDCl3 {C:\700} tds 53
f2 (ppm)
f1 (ppm)

Ethyl 5-(phenylethynyl)tetrahydrofuran-2-carboxylate – 8i

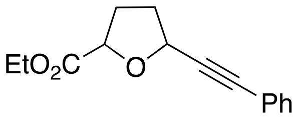

Following application of method A, purification by column chromatography (0-15% EtOAc/cyclohexane) afforded ethyl 5-(phenylethynyl)tetrahydrofuran-2-carboxylate **8i** as a clear oil (46 mg, 0.18 mmol, 76%). ^{1}H NMR (500 MHz, $CDCl_3$) δ 7.46–7.38 (m, 2H), 7.37–7.27 (m, 3H), 5.12–5.06 (m, 1H), 4.67 (dd, *J* = 8.3, 5.3 Hz, 1H), 4.23 (dq, *J* = 11.9, 7.1 Hz, 1H), 4.20 (dq, *J* = 11.9, 7.1 Hz, 1H), 2.53–2.41 (m, 1H), 2.38–2.27 (m, 1H), 2.18–2.05 (m, 2H), 1.29 (t, *J* = 7.1 Hz, 1H). ^{13}C NMR (126 MHz, $CDCl_3$) δ 172.8 (C=O), 131.9 (CH), 128.5 (CH), 128.4 (CH), 122.7 (C), 88.0 (C), 85.4 (C), 70.2 (CH_2), 61.2 (CH), 32.9 (CH_2), 29.9 (CH_2), 14.3 (CH_3); IR (thin film): 2921, 2199, 1743, 1575, 1547 cm^{-1}; HRMS (ESI) calcd for $C_{15}H_{16}O_3$ $[M+H]^+$ 245.1172; observed 245.1170.

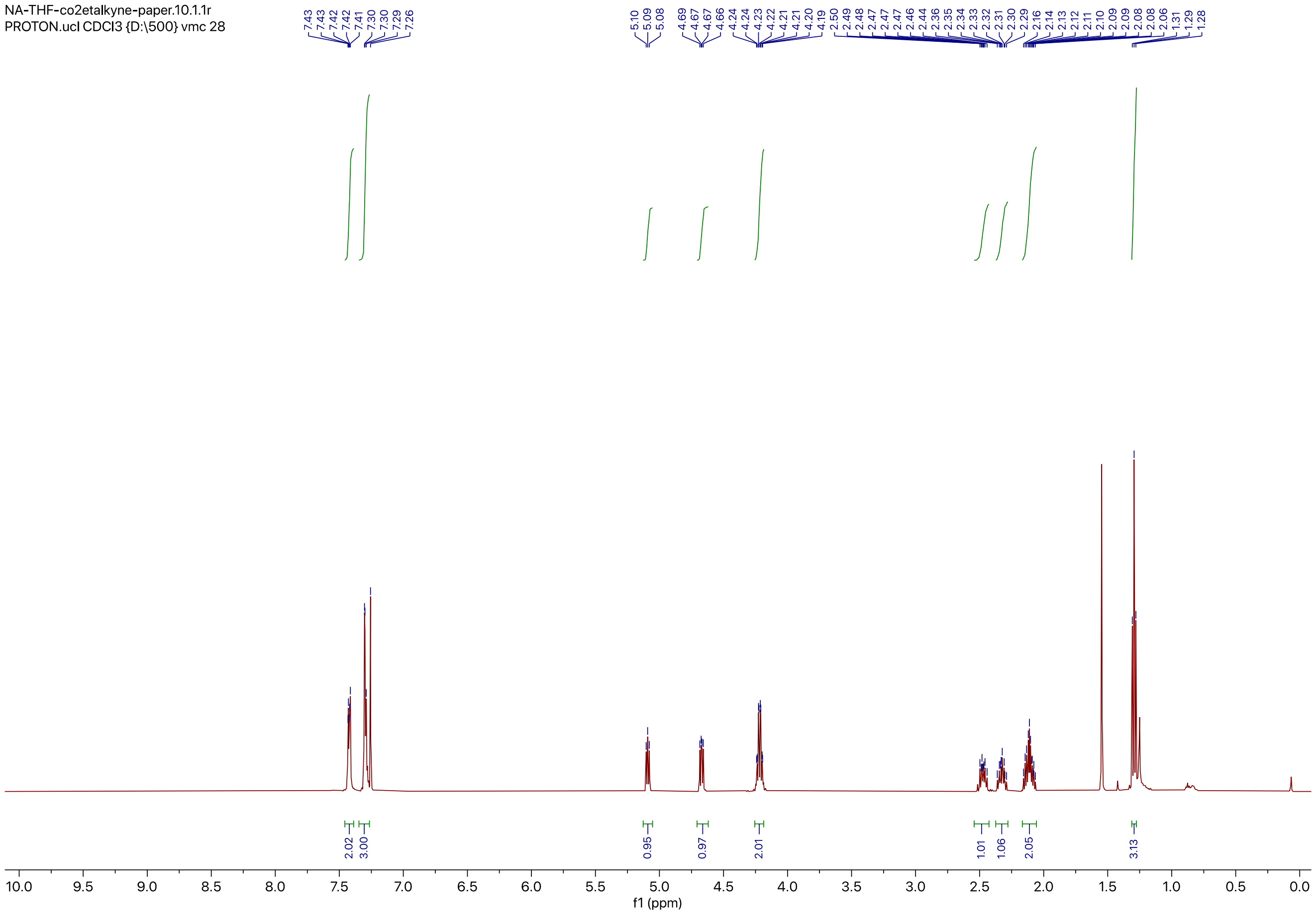

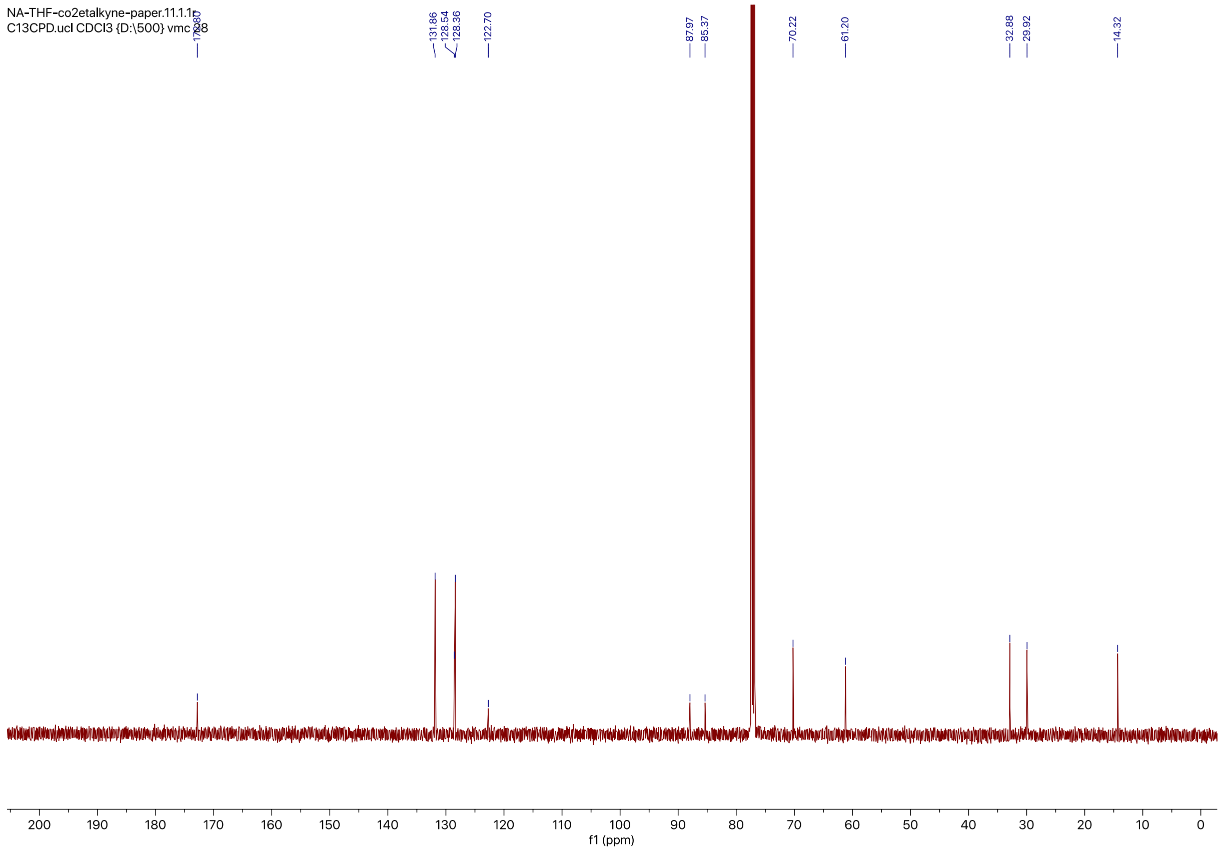

NA-THF-co2etalkyne-paper.11.1.1r
C13CPD.ucl CDCl3 {D:\500} vmc 28
172.87
131.86
128.54
128.36
122.70
87.97
85.37
70.22
61.20
32.88
29.92
14.32
200 190 180 170 160 150 140 130 120 110 100 90 80 70 60 50 40 30 20 10 0
f1 (ppm)

2,2-Dimethyl-4-(phenylethynyl)-1,3-dioxolane – 8j

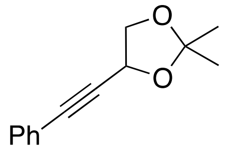

Following application of method A, purification by column chromatography (0-15% EtOAc/cyclohexane) afforded 2,2-dimethyl-4-(phenylethynyl)-1,3-dioxolane **8j** as a clear oil (46 mg, 0.18 mmol, 76%). ^{1}H NMR (300 MHz, $CDCl_3$) δ 7.48–7.41 (m, 2H), 7.31 (m, 3H), 4.95 (t, *J* = 6.4 Hz, 1H), 4.24 (dd, *J* = 8.0, 6.3 Hz, 1H), 4.02 (dd, *J* = 8.0, 6.3 Hz, 1H), 1.55 (s, 3H), 1.42 (s, 3H); ^{13}C NMR (151 MHz, $CDCl_3$) δ 131.9 (CH), 128.8 (CH), 128.4 (CH), 122.4 (C), 110.5 (C), 86.3 (C), 86.0 (C), 70.2 (CH), 66.2 (CH_2), 26.4 (CH_3), 26.2 (CH_3). IR (thin film): 3001, 2923, 2204, 1601, 1575, 1557 cm^{-1}. Known Compound.[4]

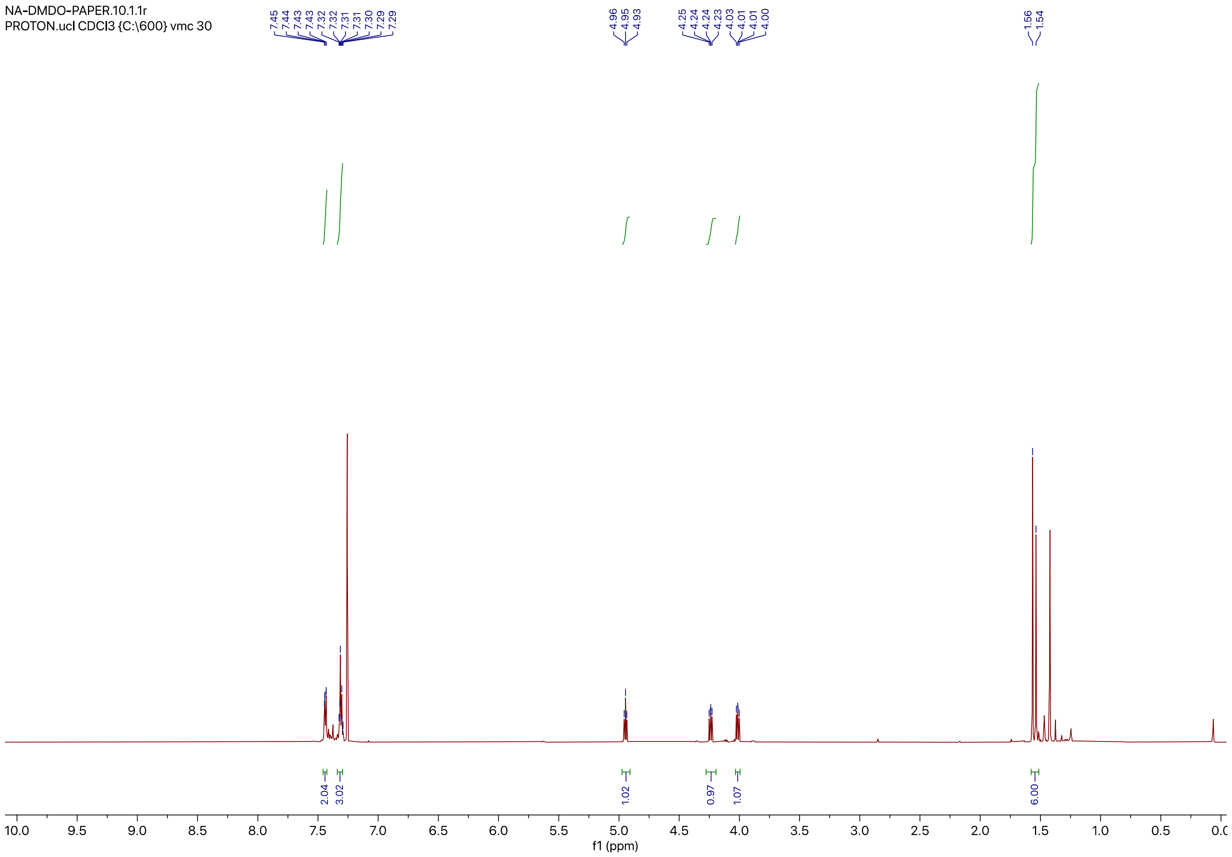

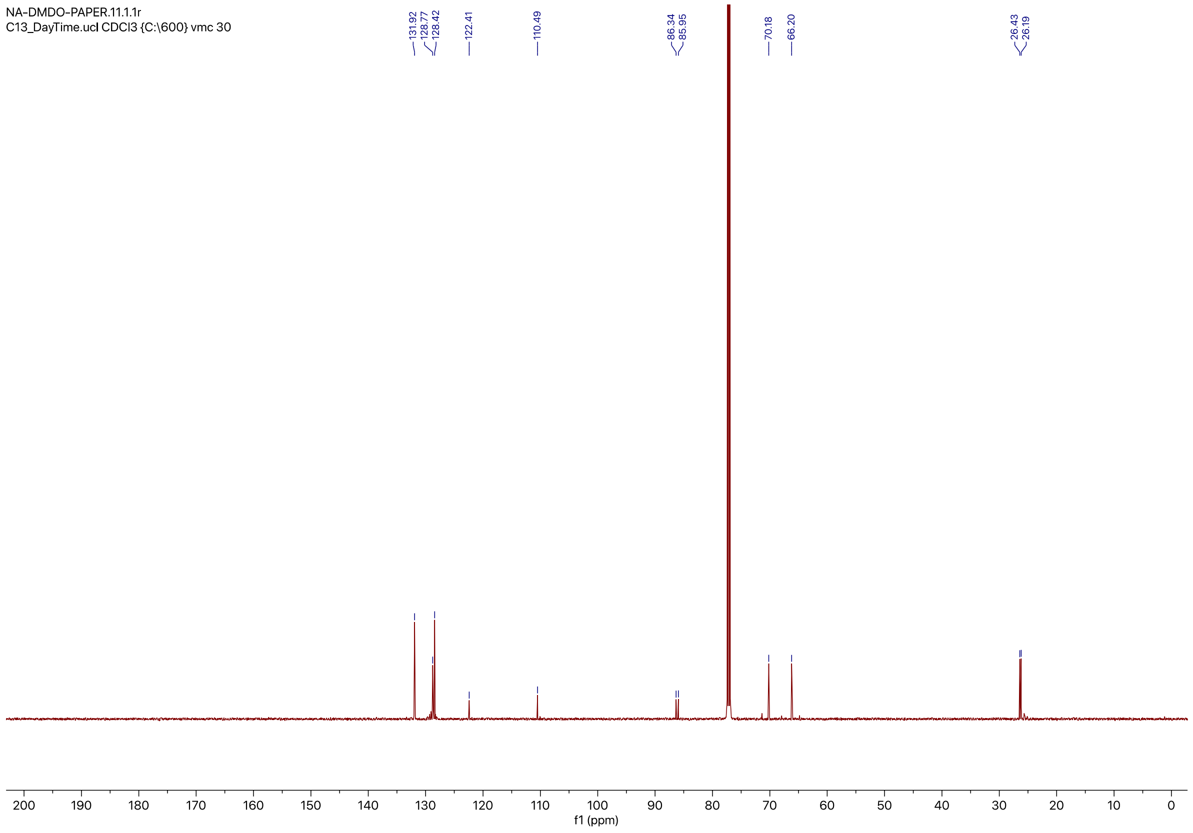
NA-DMDO-PAPER.11.1.1r
C13_DayTime.ucl CDCl3 {C:\600} vmc 30
131.92
128.77
128.42
122.41
110.49
86.34
85.95
70.18
66.20
26.43
26.19
200 190 180 170 160 150 140 130 120 110 100 90 80 70 60 50 40 30 20 10 0
f1 (ppm)

2-(Phenylethynyl)tetrahydro-2*H*-pyran – 8k

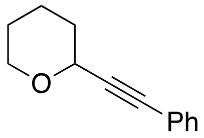

Following application of method A (10 equivalents of THP), purification by column chromatography (0-15% EtOAc/cyclohexane) afforded 2-(phenylethynyl)tetrahydro-2*H*-pyran **8k** as a clear oil (30 mg, 0.16 mmol, 64%). ^{1}H NMR ($CDCl_3$, 600 MHz): δ 7.45–7.44 (m, 2H), 7.31–7.29 (m, 3H), 4.52 (dd, J = 7.9, 2.7 Hz, 1H), 4.06–4.04 (m, 1H), 3.57–3.53 (m, 1H), 1.93–1.90 (m, 2H), 1.79–1.76 (m, 1H), 1.61–1.59 (m, 3H); ^{13}C NMR (151 MHz, $CDCl_3$) δ 131.9 (CH), 128.4 (CH), 128.4 (CH), 122.9 (C), 88.2 (C), 85.3 (C), 67.6 (CH), 66.8 (CH_2), 32.3 (CH_2), 25.8 (CH_2), 22.0 (CH_2); IR (thin film): 2945, 2901, 2211, 1608, 1562, 1553 cm^{-1}. Known compound.[2]

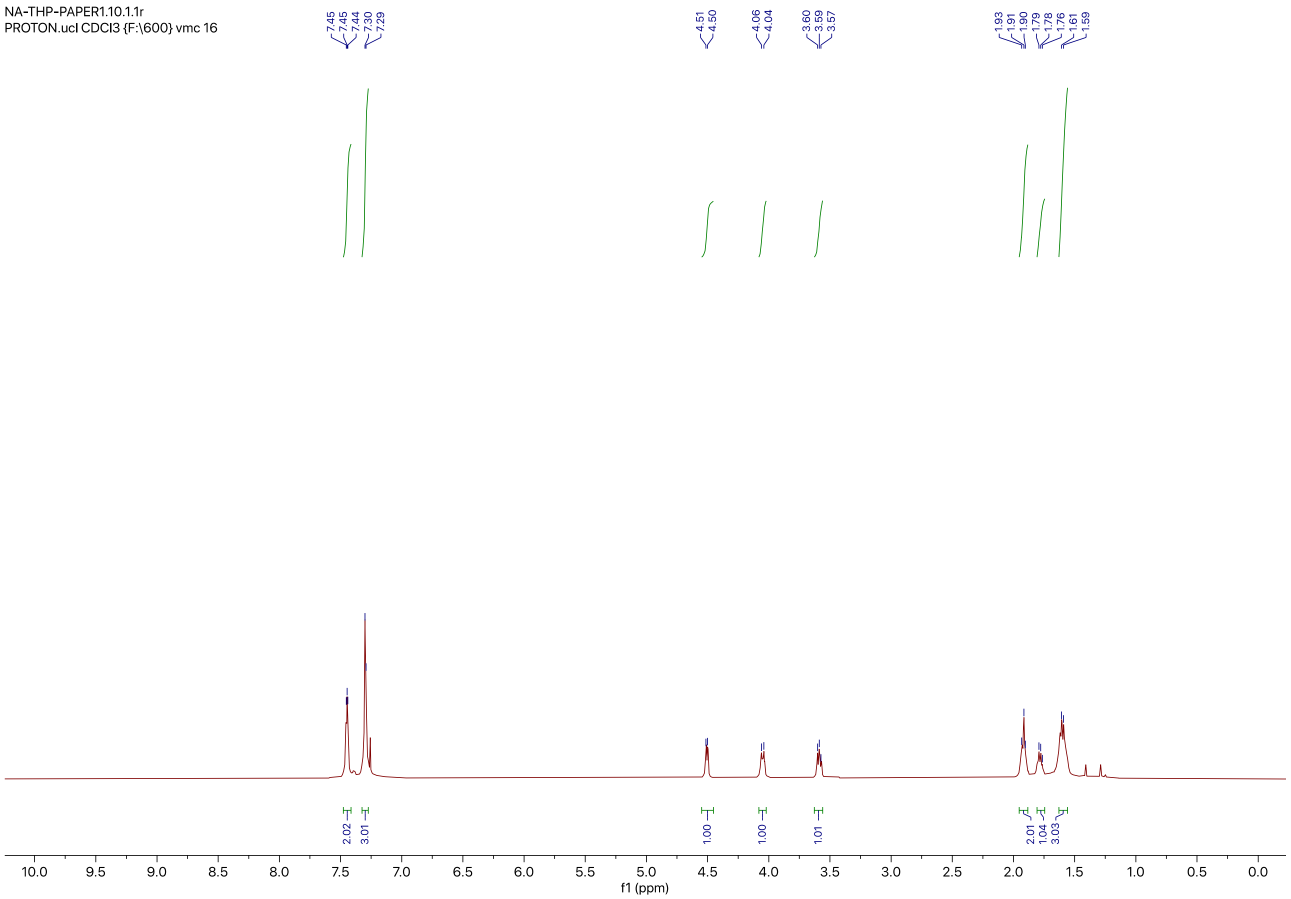

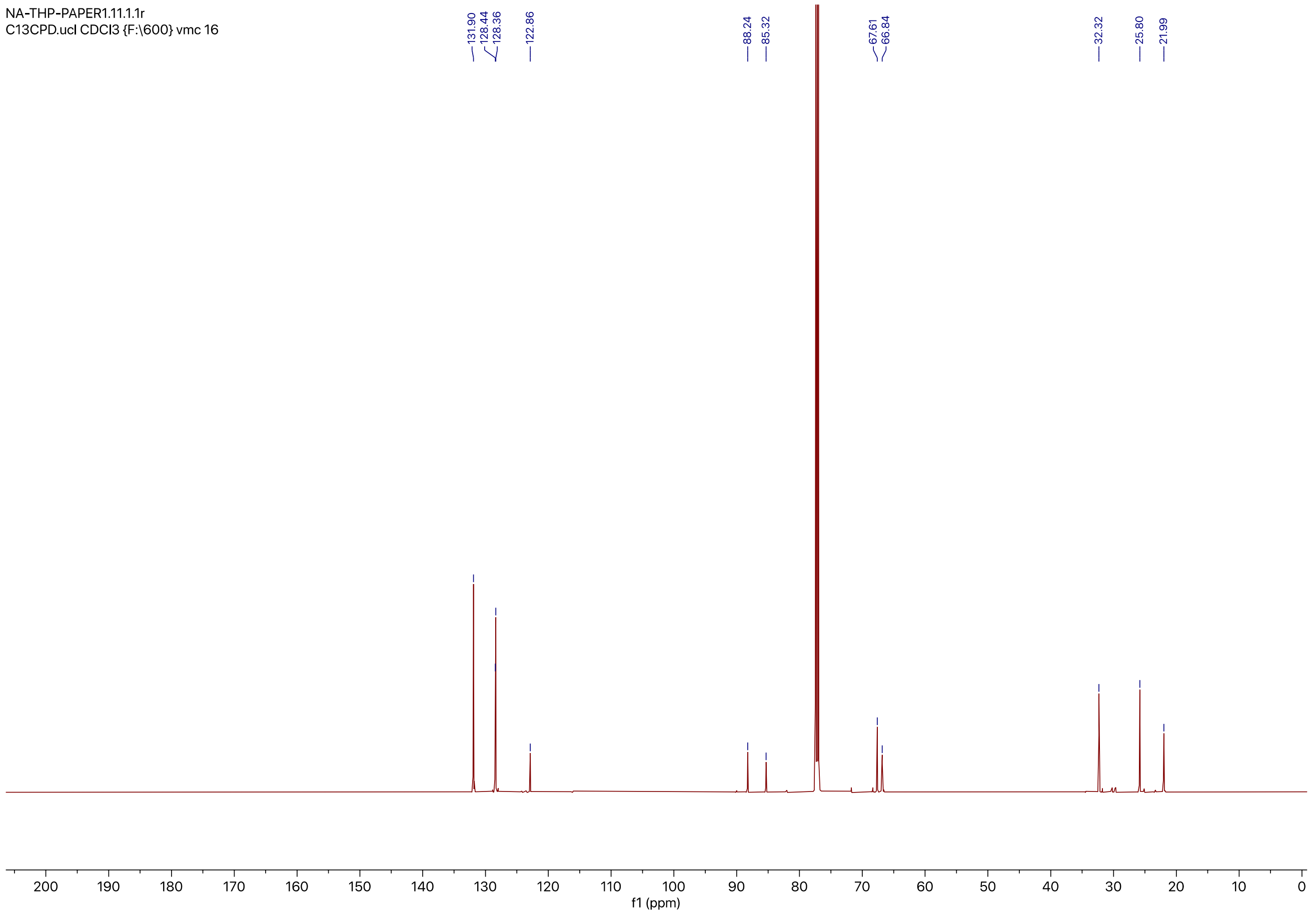
NA-THP-PAPER1.11.1.1r
C13CPD.ucl CDCl3 {F:\600} vmc 16
131.90
128.44
128.36
122.86
88.24
85.32
67.61
66.84
32.32
25.80
21.99
200 190 180 170 160 150 140 130 120 110 100 90 80 70 60 50 40 30 20 10 0
f1 (ppm)

(2*R*,3*S*,4*R*,6*S*)-2-(Acetoxymethyl)-6-(phenylethynyl)tetrahydro-2*H*-pyran-3,4-diyl diacetate and (2*R*,3*S*,4*R*,6*R*)-2-(acetoxymethyl)-6-(phenylethynyl)tetrahydro-2*H*-pyran-3,4-diyl diacetate – 8l-a and 8l-b

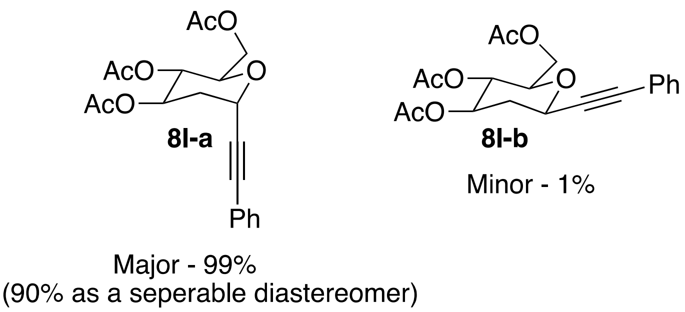

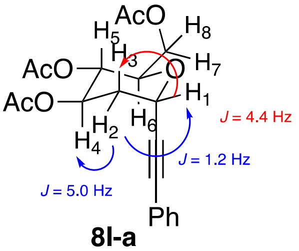

8l-a

Following application of method A, purification by column chromatography (0-25% EtOAc/cyclohexane) afforded single diastereomer (2*R*,3*S*,4*R*,6*S*)-2-(acetoxymethyl)-6-(phenylethynyl)tetrahydro-2*H*-pyran-3,4-diyl diacetate **8l-a** as a yellow oil (41 mg, 0.11 mmol, 41%). A inseparable mixture of **8l-a** and **8l-b** was also isolated, the yield and assignment quoted is of the pure diastereomer **8l-a**. Stereochemical assignment is based on J coupling values, in particular of equatorial C-H_1. ^{1}H NMR ($CDCl_3$, 600 MHz): δ ^{1}H NMR (600 MHz, $CDCl_3$) δ 7.53–7.46 (m, 2H), 7.38–7.29 (m, 3H), 5.43 (ddd, *J* = 11.6, 9.5, 5.0 Hz, 1H, H_4), 5.11 (br d, *J* = 4.4 Hz, 1H, H_1), 5.01 (t, *J* = 9.7 Hz, 1H, H_5), 4.35 (dd, *J* = 12.3, 4.2 Hz, 1H, H_7), 4.23 (ddd, *J* = 9.9, 4.2, 2.1 Hz, 1H, H_6), 4.10 (dd, *J* = 12.3, 2.1 Hz, 1H, H_8), 2.34 (ddd, *J* = 12.8, 5.0, 1.5 Hz, 1H, H_2), 2.10 (s, 3H), 2.07 (m, 1H, H_3), 2.04 (s, 3H), 2.03 (s, 3H). ^{13}C NMR (151 MHz, $CDCl_3$) δ 171.0 (C=O), 170.4 (C=O), 170.1 (C=O), 132.1 (CH), 129.1 (CH), 128.5 (CH), 121.9 (C), 88.7 (C), 84.6 (C), 71.4 (CH), 70.1 (CH), 69.4 (CH), 64.7 (CH), 62.5 (CH), 35.6 (CH_2), 21.2 (CH_3), 21.0 (CH_3), 20.9 (CH_3). IR (thin film): 2961, 2867, 2214, 1741, 1501, 1440 cm^{-1}. HRMS (ESI) calcd for $C_{20}H_{22}O_7$ $[M+H]^+$ 375.1444; observed 375.1436.

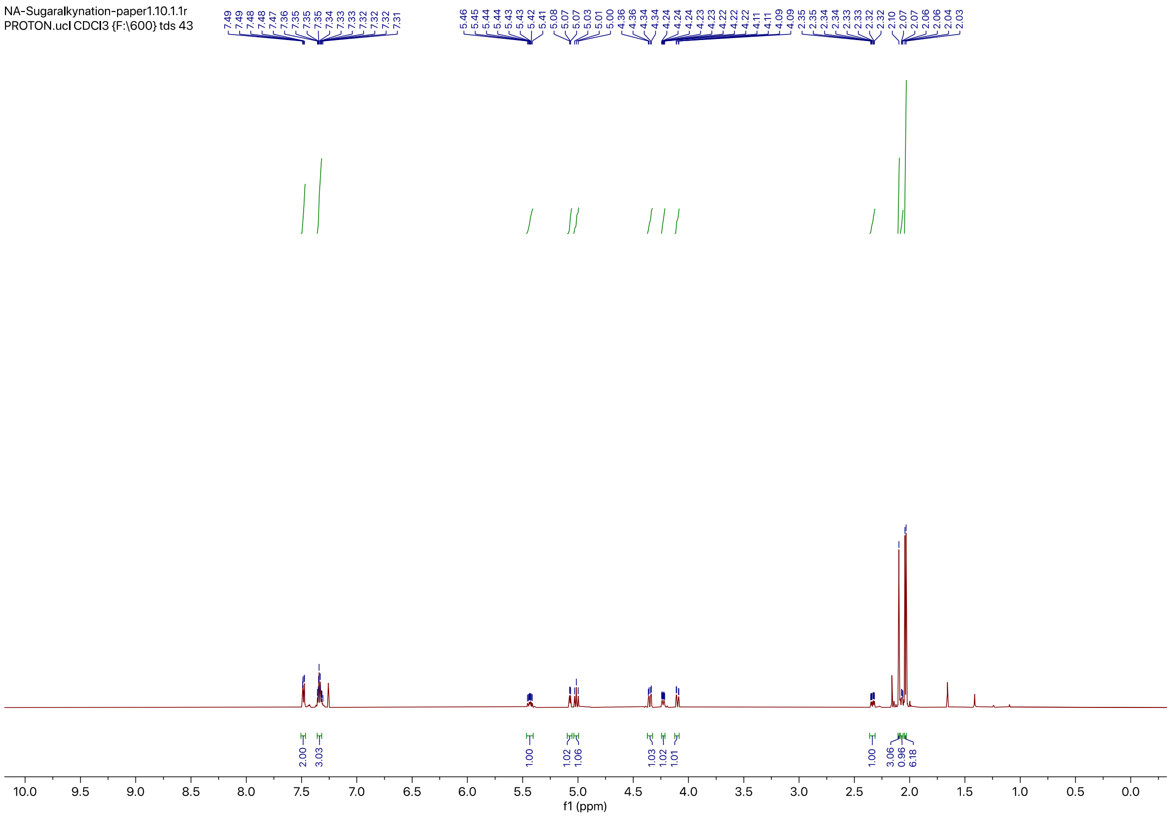
NA-Sugaralkynation-paper1.10.1.1r
PROTON.ucl CDCl3 {F:\600} tds 43
f1 (ppm)

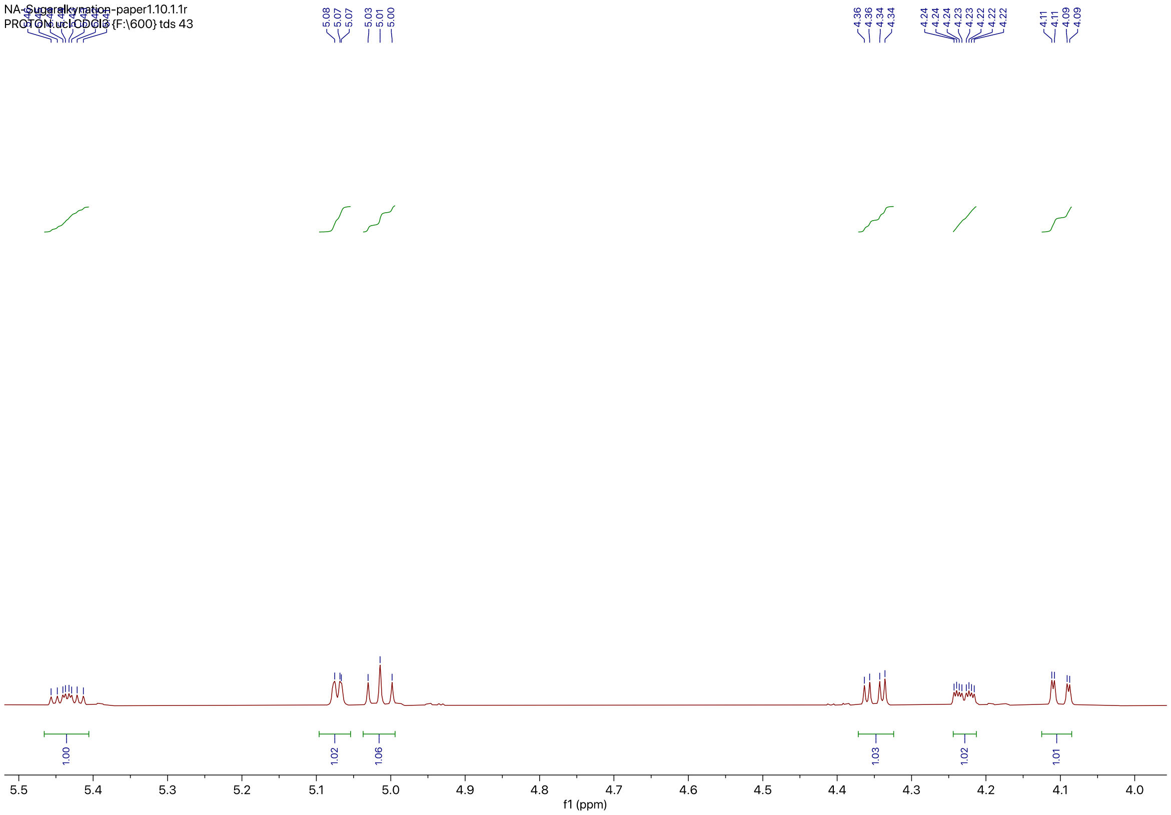
NA-Sugaralkynation-paper1.10.1.1r
PROTON.ucl CDCl3 {F:\600} tds 43
f1 (ppm)

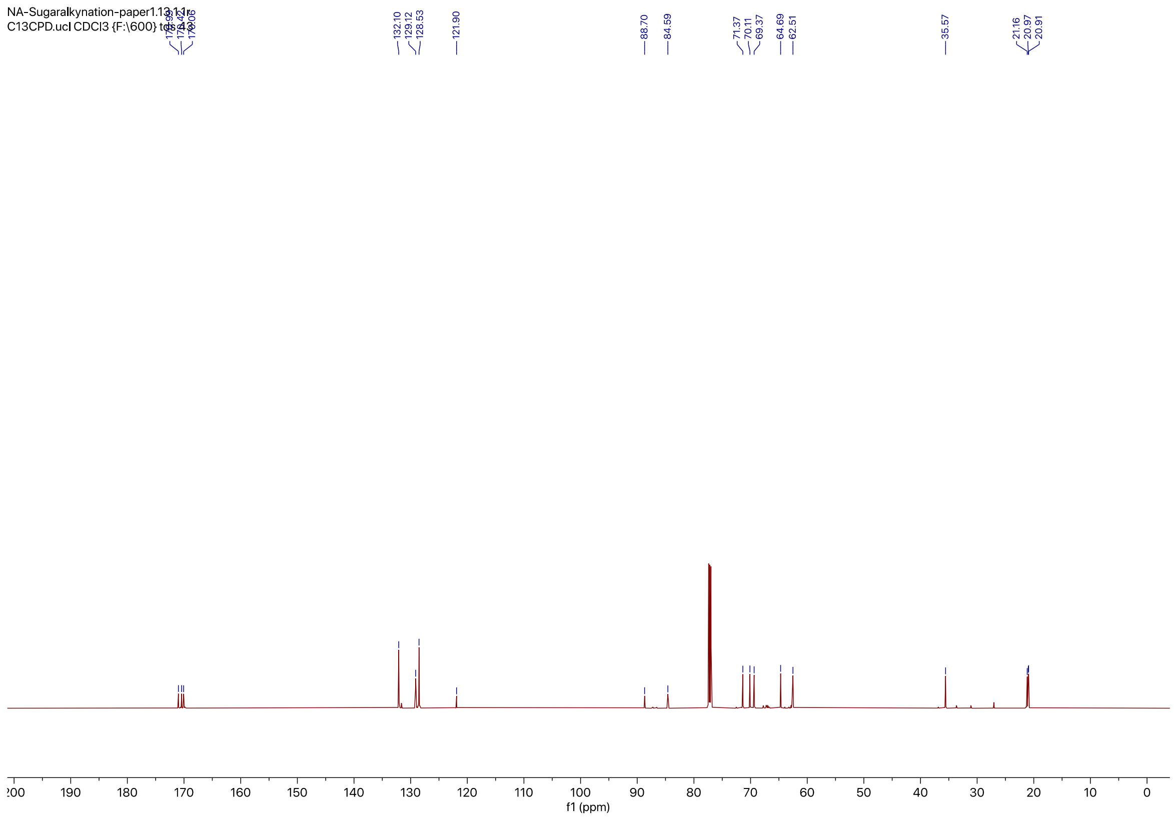
NA-Sugaralkynation-paper1.13.1.1r
C13CPD.ucl CDCl3 {F:\600} tds 43
170.99
170.42
170.06
132.10
129.12
128.53
121.90
88.70
84.59
71.37
70.11
69.37
64.69
62.51
35.57
21.16
20.97
20.91
f1 (ppm)

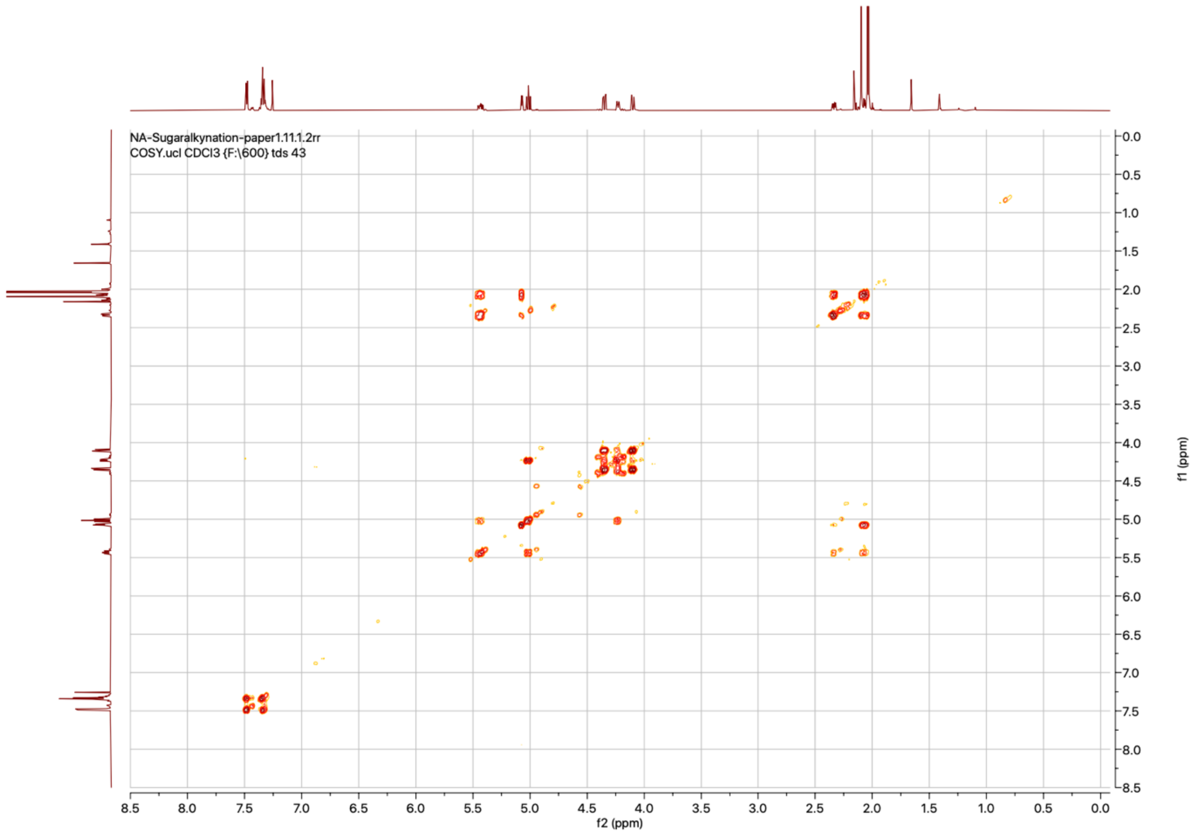
NA-Sugaralkynation-paper1.11.1.2rr
COSY.ucl CDCl3 {F:\600} tds 43
f2 (ppm)
f1 (ppm)

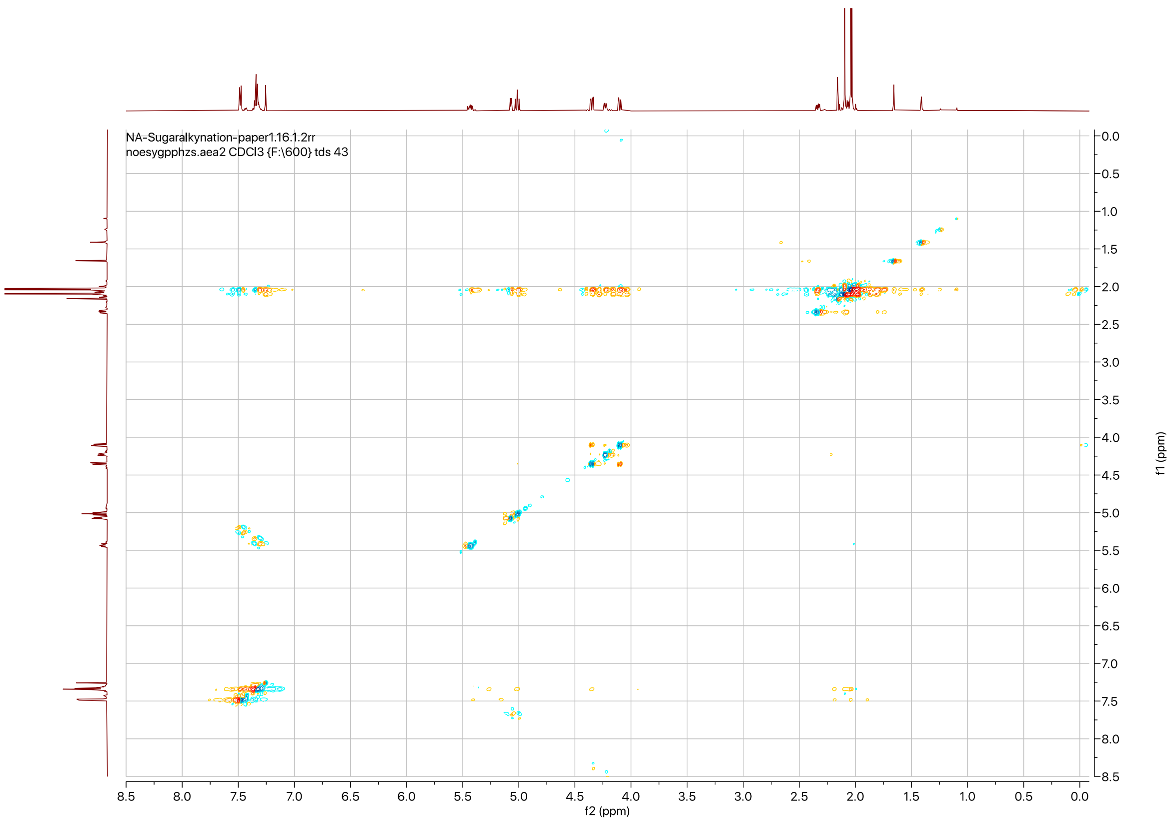
NA-Sugaralkynation-paper1.16.1.2rr
noesygpphzs.aea2 CDCl3 {F:\600} tds 43
f2 (ppm)
f1 (ppm)

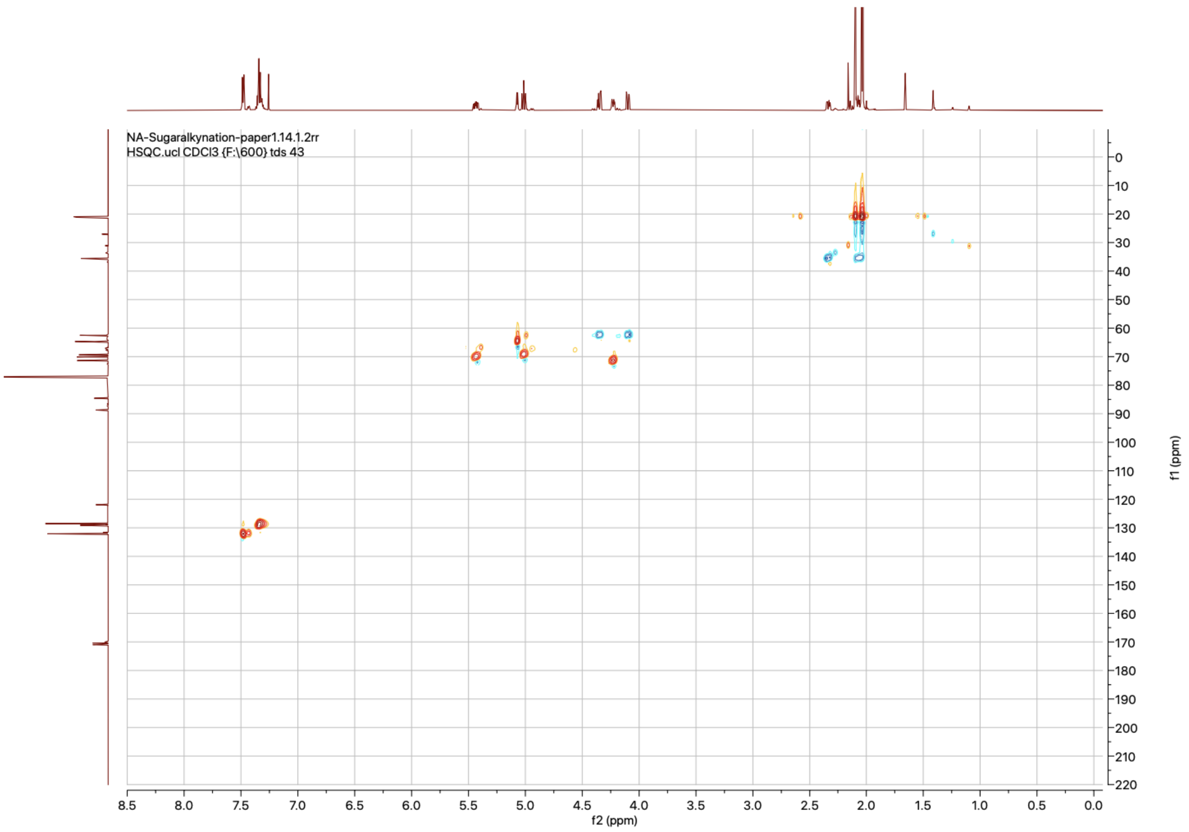
NA-Sugaralkynation-paper1.14.1.2rr
HSQC.ucl CDCl3 {F:\600} tds 43
f2 (ppm)
f1 (ppm)

2-(Phenylethynyl)-1,4-dioxane – 8m

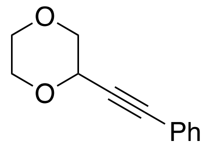

Following application of method A, purification by column chromatography (0-15% EtOAc/cyclohexane) afforded 2-(phenylethynyl)-1,4-dioxane **8m** as a yellow oil (33 mg, 0.18 mmol, 71%). ^{1}H NMR ($CDCl_3$, 600 MHz): δ 7.45 (dd, *J* = 6.2, 1.8 Hz, 2H), 7.31–7.29 (m, 3H), 4.57 (dd, *J* = 8.2, 1.6 Hz, 1H), 3.95–3.92 (m, 2H), 3.77–3.68 (m, 4H); ^{13}C NMR (151 MHz, $CDCl_3$) δ 132.0 (CH), 128.9 (CH), 128.4 (CH), 122.2 (CH_2), 86.7 (C), 84.4 (C), 70.7 (CH), 66.6 (CH_2), 66.5 (CH_2), 66.0 (CH_2); IR (thin film): 2905, 2892, 2199, 1613, 1560, 1546 cm^{-1}. Known compound.[2]

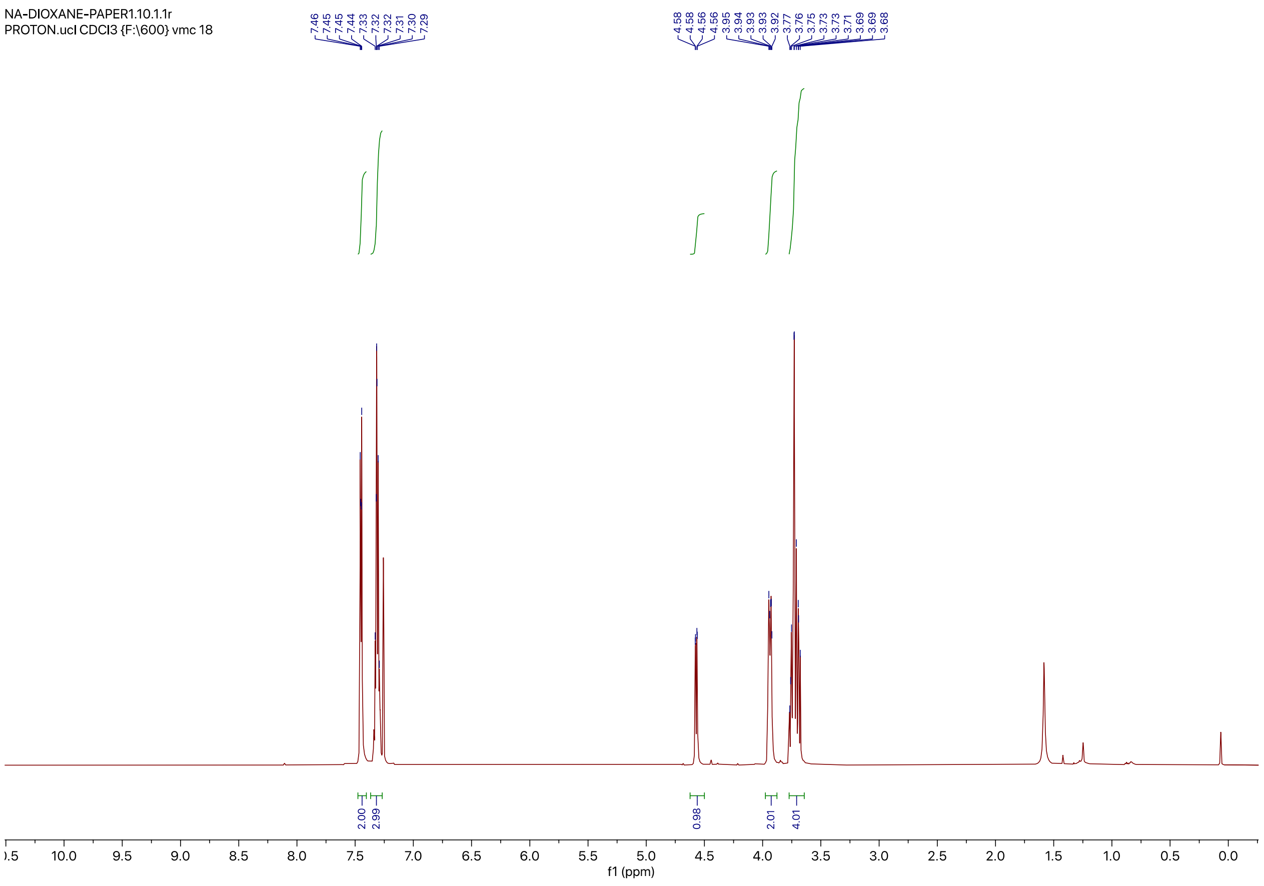

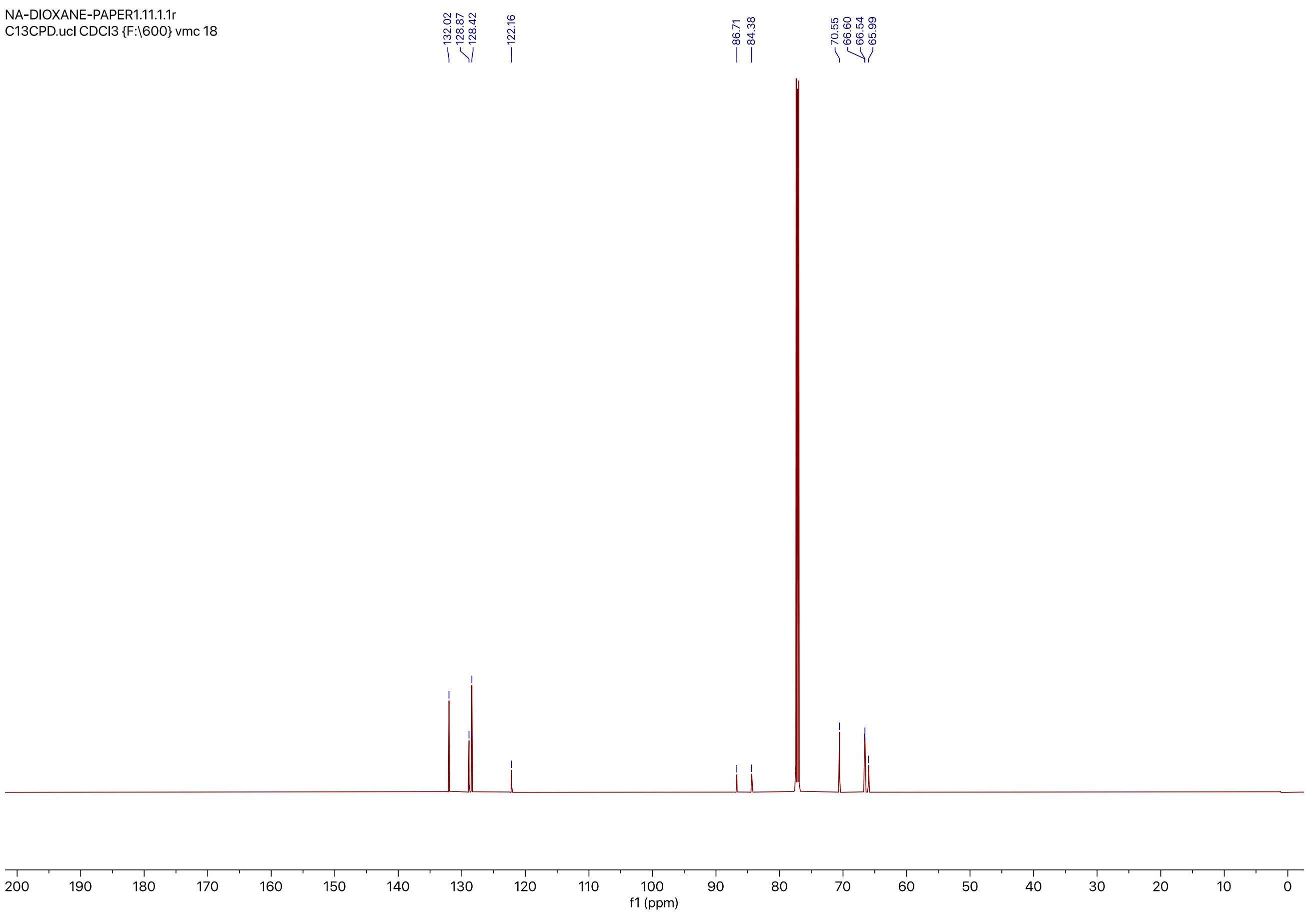

NA-DIOXANE-PAPER1.11.1.1r
C13CPD.ucl CDCl3 {F:\600} vmc 18
132.02
128.87
128.42
122.16
86.71
84.38
70.55
66.60
66.54
65.99
200 190 180 170 160 150 140 130 120 110 100 90 80 70 60 50 40 30 20 10 0
f1 (ppm)

3-(Phenylethynyl)-1,4-oxathiane and 2-(phenylethynyl)-1,4-oxathiane – 8p + 8q

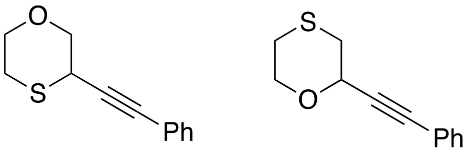

59%, 10:1, **8p**:**8q**

Following application of method A, purification by column chromatography (0-15% EtOAc/cyclohexane) afforded 3-(phenylethynyl)-1,4-oxathiane and 2-(phenylethynyl)-1,4-oxathian **8p** and **8q** as an inseparable mixture of regioisomers – yellow oil (30 mg, 0.15 mmol, 59%). **8q** (major): ^{1}H NMR (600 MHz, $CDCl_3$) δ 7.51–7.38 (m, 2H), 7.36–7.27 (m, 3H), 4.20 (d, J = 8.9 Hz, 1H), 4.05 (ddd, J = 11.8, 4.7, 2.9 Hz, 1H), 3.93–3.79 (m, 3H), 2.85 (ddd, J = 13.6, 8.9, 2.9 Hz, 1H), 2.72 (ddd, J = 13.6, 4.7, 2.2 Hz, 1H); ^{13}C NMR (151 MHz, $CDCl_3$) δ 132.0 (CH), 129.6 (CH), 128.4 (CH), 122.7 (C), 85.4 (C), 84.5 (C), 72.8 (CH_2), 68.5 (CH_2), 30.4 (CH), 27.1 (CH_2); IR (thin film): 2918, 2221, 1619, 1560, 1531 cm^{-1}. HRMS (ESI) calcd for $C_{12}H_{12}OS$ [M+H]$^+$ 205.0682; observed 205.0680.

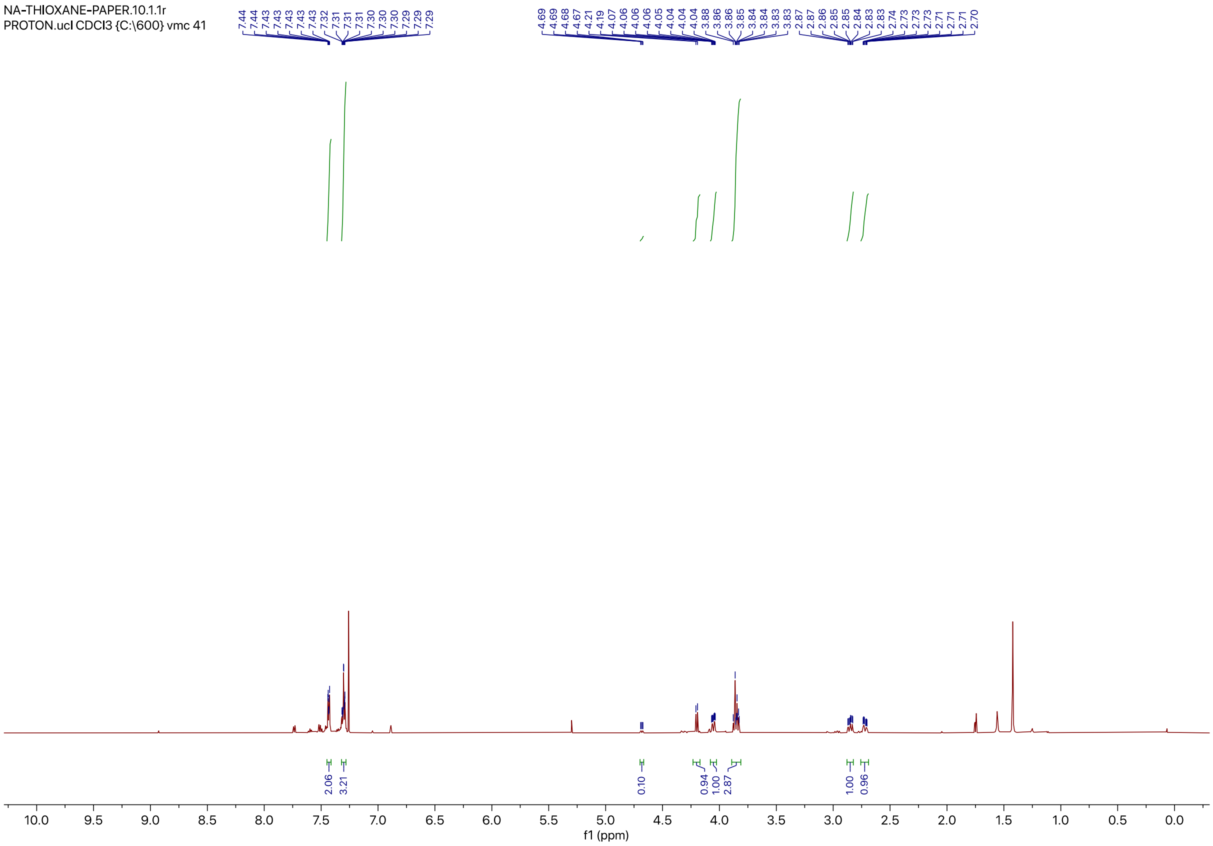

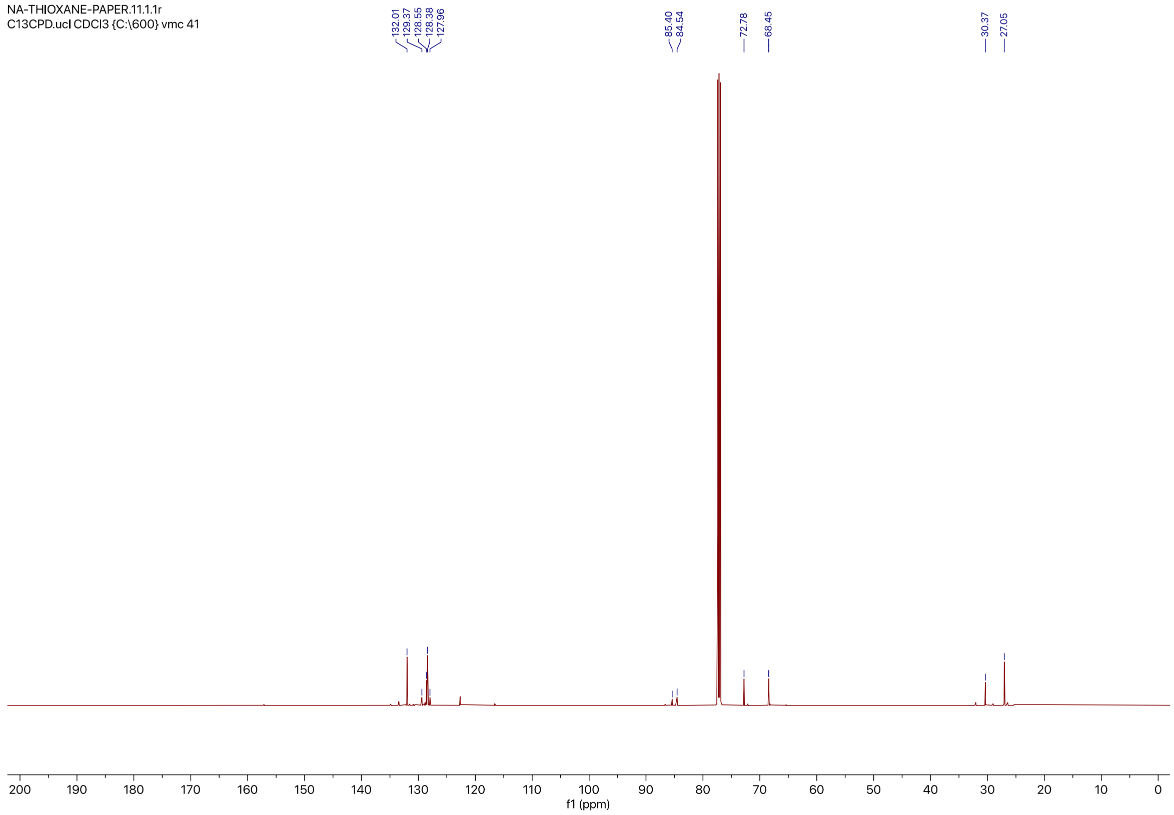
NA-THIOXANE-PAPER.11.1.1r
C13CPD.ucl CDCl3 {C:\600} vmc 41
132.01
129.37
128.55
128.38
127.96
85.40
84.54
72.78
68.45
30.37
27.05
200 190 180 170 160 150 140 130 120 110 100 90 80 70 60 50 40 30 20 10 0
f1 (ppm)

2-(Phenylethynyl)tetrahydrothiophene – 8r

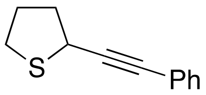

Following application of method A with the exclusion of HFIP, purification by column chromatography (0-15% EtOAc/cyclohexane) afforded 2-(phenylethynyl)tetrahydrothiophene **8r** as a yellow oil (34 mg, 0.18 mmol, 72%). ^{1}H NMR ($CDCl_3$, 500 MHz): ^{1}H NMR (500 MHz, $CDCl_3$) δ 7.40 (ddd, J = 6.0, 2.4, 1.6 Hz, 2H), 7.29–7.26 (m, 3H), 4.27 (t, J = 5.8 Hz, 1H), 3.16 –3.09 (m, 1H), 2.93 (dd, J = 10.1, 6.6 Hz, 1H), 2.30–2.13 (m, 3H), 2.12–2.04 (m, 1H). ^{13}C NMR (126 MHz, $CDCl_3$) δ 131.8 (CH), 128.3 (CH), 128.1 (CH), 123.4 (C), 90.8 (C), 83.0 (C), 39.1 (CH), 37.1 (CH_2), 33.0 (CH_2), 30.6 (CH_2). IR (thin film): 3002, 2931, 2209, 1602, 1558 cm^{-1}. Known compound.[6]

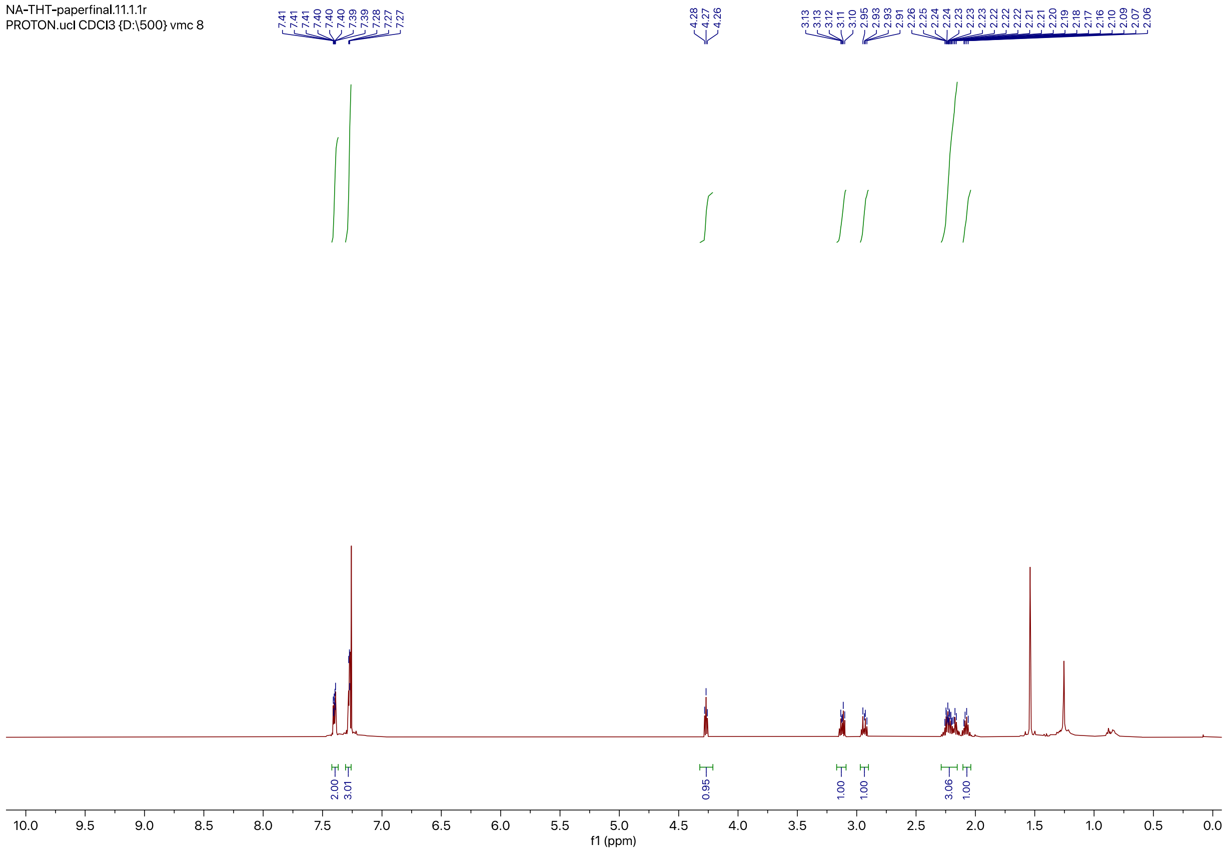

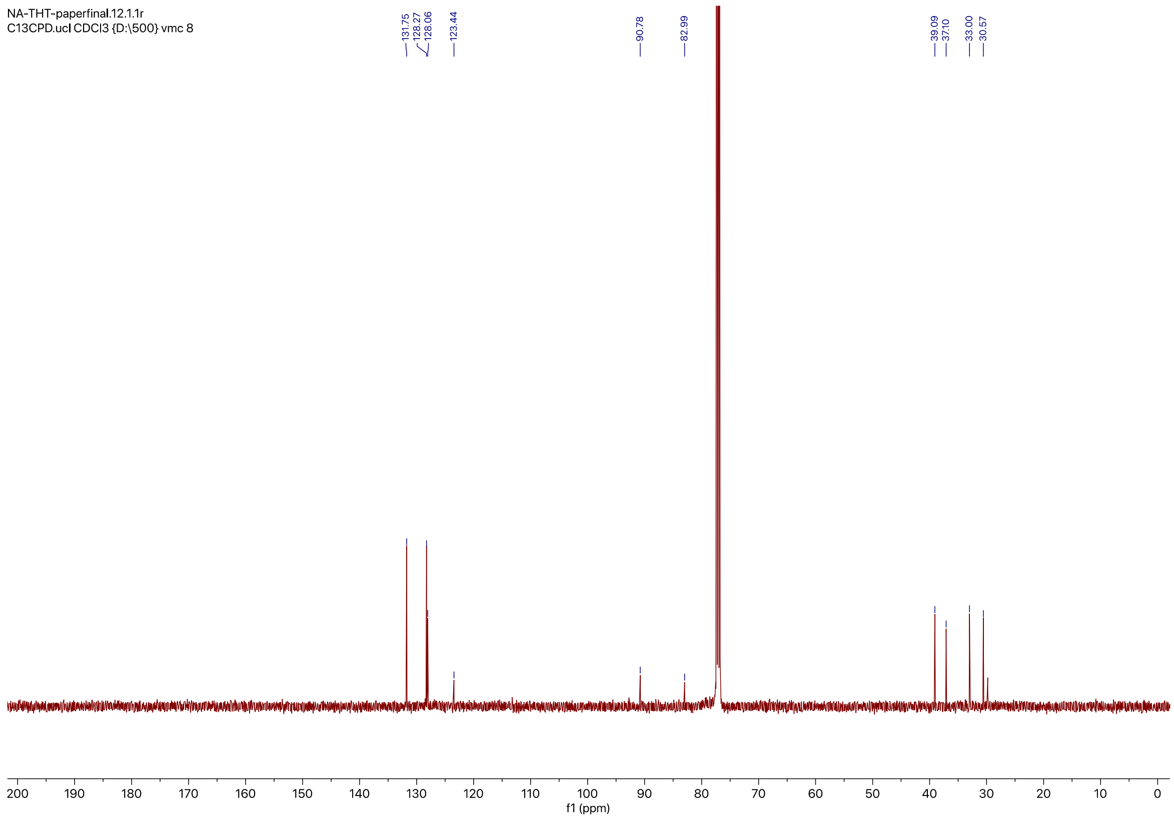

NA-THT-paperfinal.12.1.1r
C13CPD.ucl CDCl3 {D:\500} vmc 8
131.75
128.27
128.06
123.44
90.78
82.99
39.09
37.10
33.00
30.57
200 190 180 170 160 150 140 130 120 110 100 90 80 70 60 50 40 30 20 10 0
f1 (ppm)

(3-Ethoxybut-1-yn-1-yl)benzene – **8s**

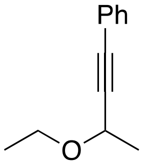

Following application of method A, purification by column chromatography (0-15% EtOAc/cyclohexane) afforded (3-ethoxybut-1-yn-1-yl)benzene **8s** as a clear oil (25 mg, 0.14 mmol, 57%). ^{1}H NMR ($CDCl_3$, 500 MHz): δ 7.45-7.43 (m, 2H), 7.31–7.29 (m, 3H), 4.39 (q, *J* = 6.6 Hz, 1H), 3.85 (dq, *J* = 14.0 Hz, 7.0, 1H), 3.51 (dq, *J* = 14.0 Hz, 7.0, 1H), 1.53 (d, *J* = 6.6 Hz, 3H), 1.26 (t, *J* = 7.0 Hz, 3H); ^{13}C NMR (126 MHz, $CDCl_3$) δ 131.8 (CH), 128.4 (CH), 123.0 (C), 89.6 (C), 84.8 (C), 65.6 (CH), 64.3 (CH_2), 22.4 (CH_3), 15.4 (CH_3); IR (thin film): 2930, 2902, 2185, 1603, 1548, 1521 cm^{-1}. Known compound.[8]

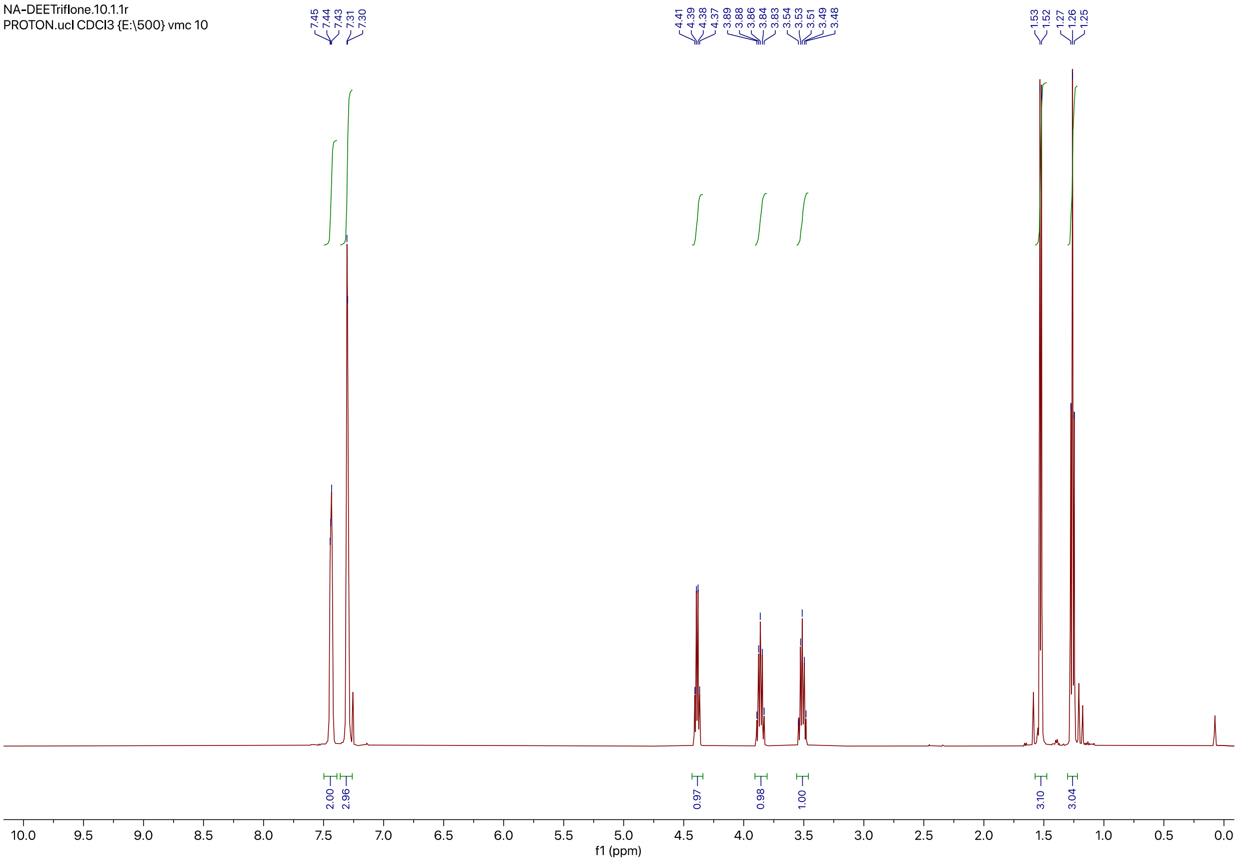

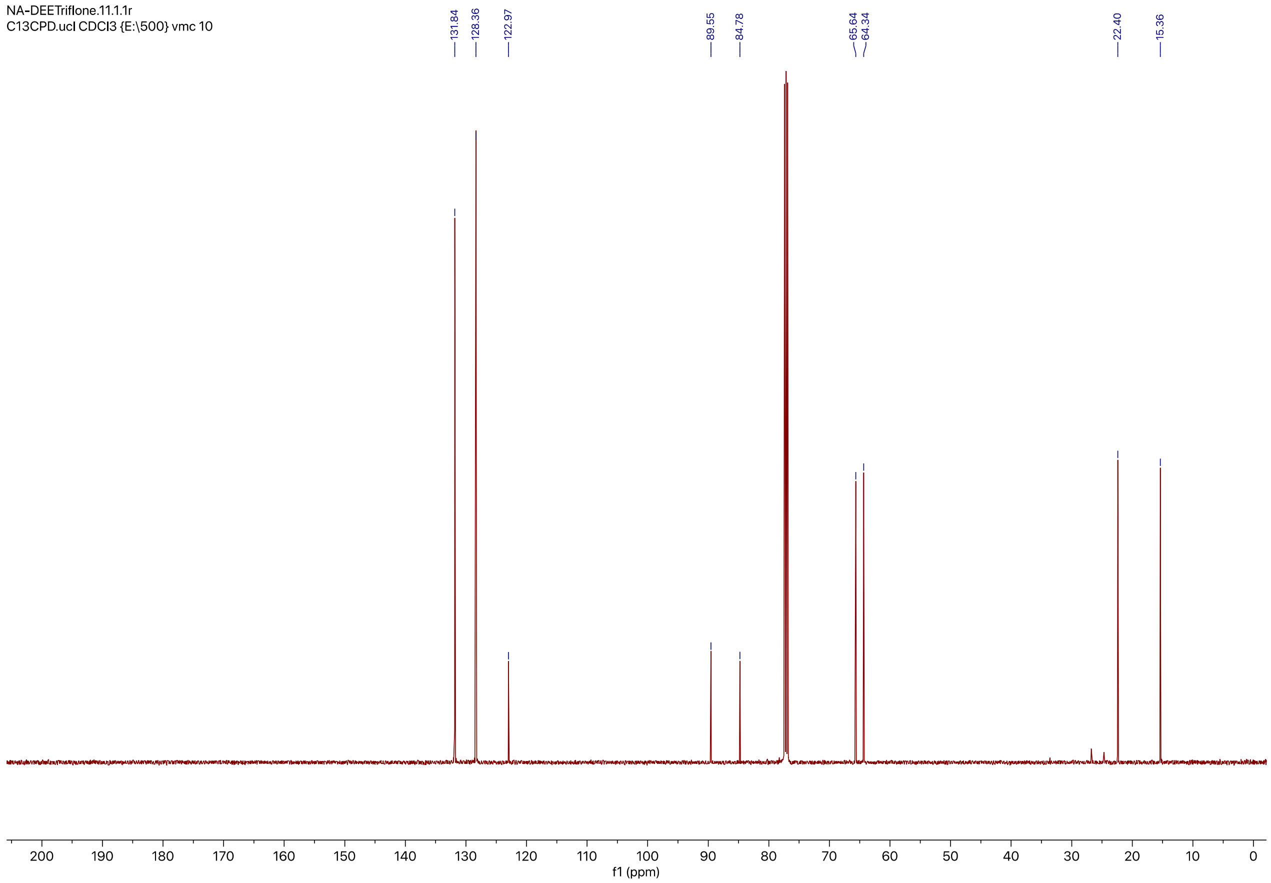
NA-DEETriflone.11.1.1r
C13CPD.ucl CDCl3 {E:\500} vmc 10
131.84
128.36
122.97
89.55
84.78
65.64
64.34
22.40
15.36
200
190
180
170
160
150
140
130
120
110
100
90
80
70
60
50
40
30
20
10
0
f1 (ppm)

(3,4-Dimethoxybut-1-yn-1-yl)benzene – 8t

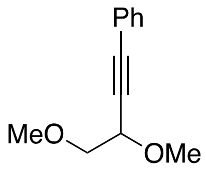

Following application of method A, purification by column chromatography (0-15% EtOAc/cyclohexane) afforded (3,4-dimethoxybut-1-yn-1-yl)benzene **8t** as a yellow oil (36 mg, 0.19 mmol, 76%). ^{1}H NMR (500 MHz, $CDCl_3$) δ 7.48–7.42 (m, 2H), 7.38–7.28 (m, 3H), 4.41 (dd, *J* = 7.4, 3.8 Hz, 1H), 3.68 (dd, *J* = 10.5, 7.4 Hz, 1H), 3.64 (dd, *J* = 10.5, 3.8 Hz, 1H), 3.53 (s, 3H), 3.45 (s, 3H); ^{13}C NMR (126 MHz, $CDCl_3$) δ 132.0 (CH), 128.7 (CH), 128.4 (CH), 122.5 (C), 87.0 (C), 85.1 (C), 75.1 (CH), 71.3 (CH_2), 59.5 (CH_3), 57.0 (CH_3); IR (thin film): 2920, 2903, 2197, 1548, 1521 cm^{-1}. Known compound.[2]

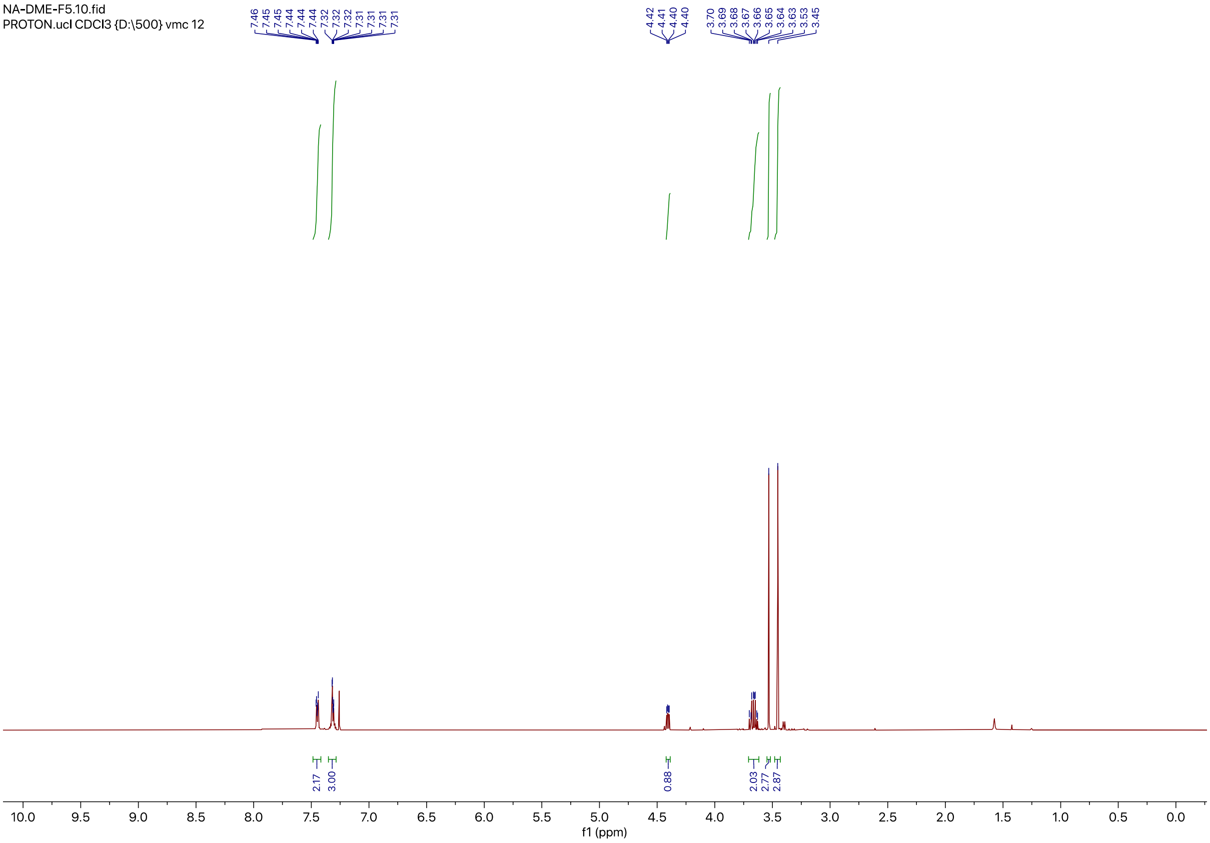

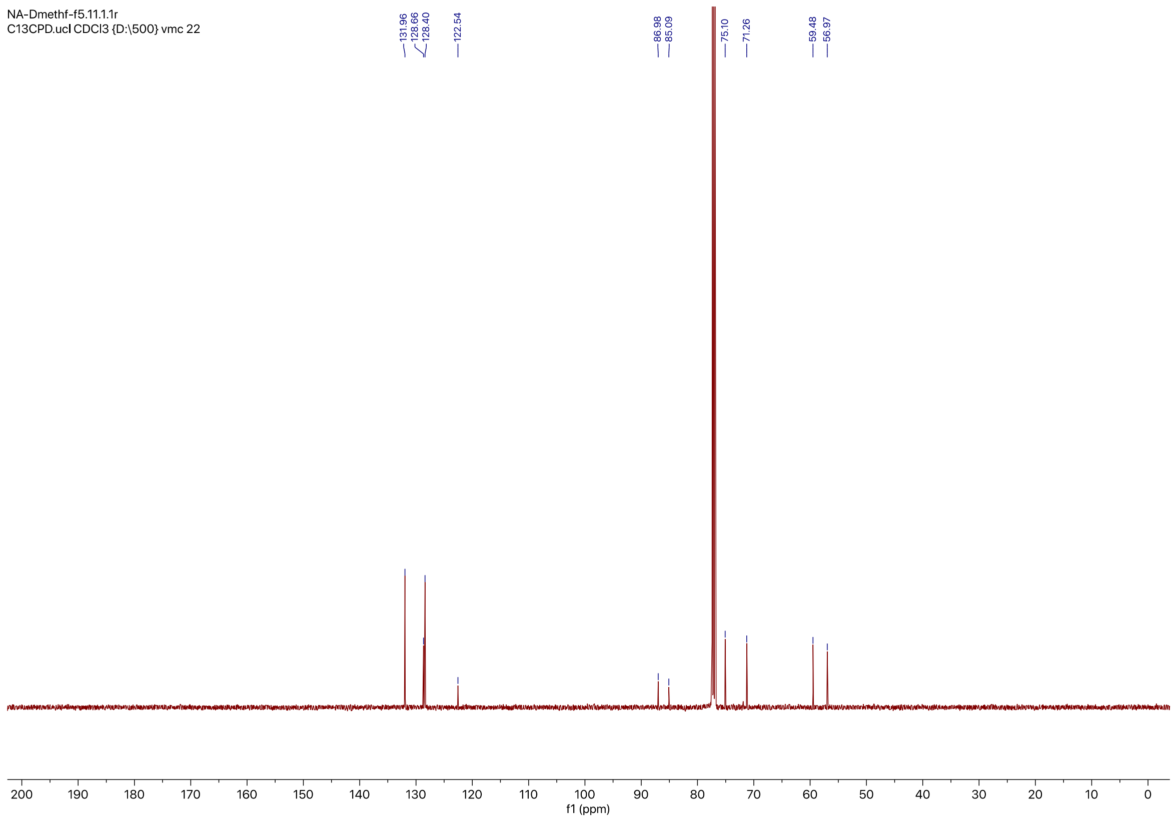
NA-Dmethf-f5.11.1.1r
C13CPD.ucl CDCl3 {D:\500} vmc 22
131.96
128.66
128.40
122.54
86.98
85.09
75.10
71.26
59.48
56.97
200
190
180
170
160
150
140
130
120
110
100
90
80
70
60
50
40
30
20
10
0
f1 (ppm)

(Cyclopent-1-en-1-ylethynyl)benzene – 8u'

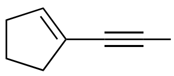

Following application of method A, purification by column chromatography (0-15% EtOAc/cyclohexane) afforded (cyclopent-1-en-1-ylethynyl)benzene **8u'** as a clear oil (37 mg, 0.22 mmol, 89%). ^{1}H NMR (500 MHz, MeOD) δ 7.43–7.36 (m, 2H), 7.35–7.27 (m, 3H), 6.09 (ddd, J = 4.6, 2.6, 2.1 Hz, 1H), 2.50 (m, 4H), 1.96 (m, 2H). ^{13}C NMR (126 MHz, MeOD) δ 138.6 (CH), 132.3 (CH), 129.4 (CH), 129.0 (CH), 126.0 (C), 125.0 (C), 91.3 (C), 87.4 (C), 37.3 (CH_2), 34.1 (CH_2), 24.3 (CH_2). IR (thin film): 2944, 2204, 1651, 1550, 1527, 1496 cm^{-1}. Known compound.[5]

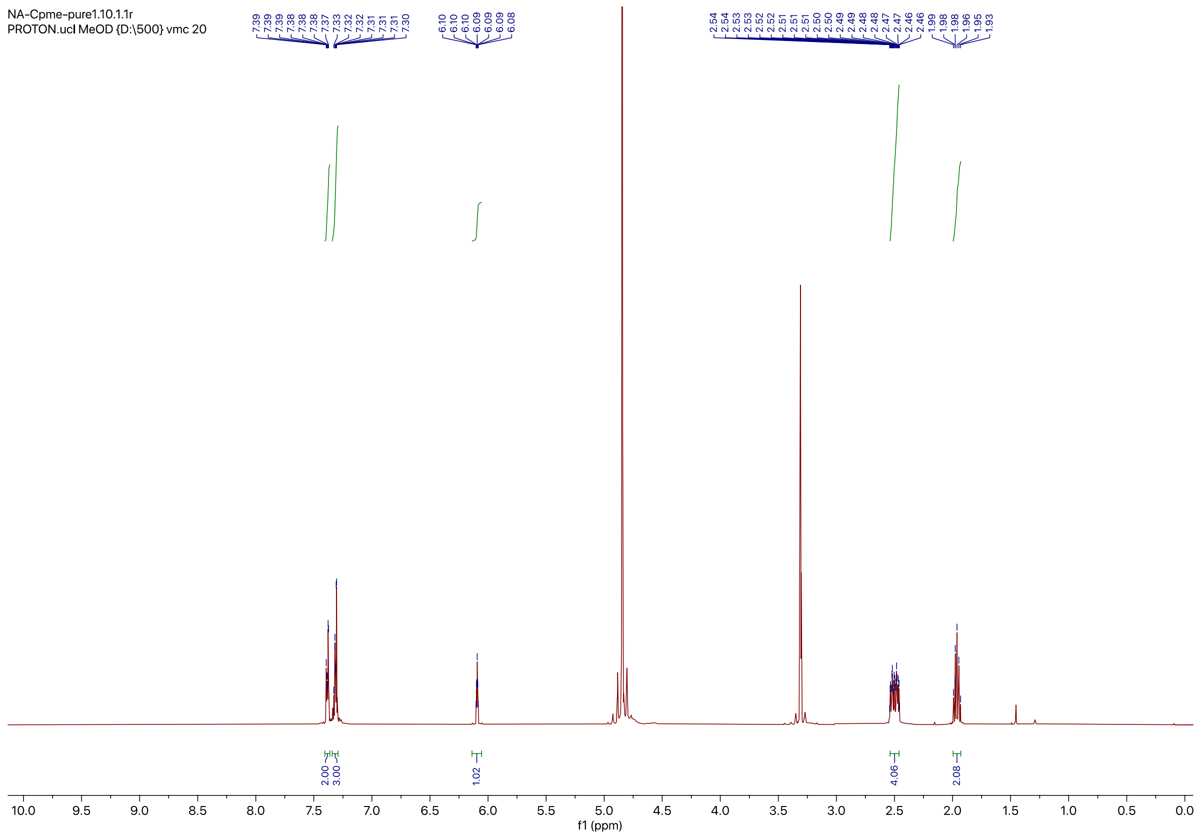

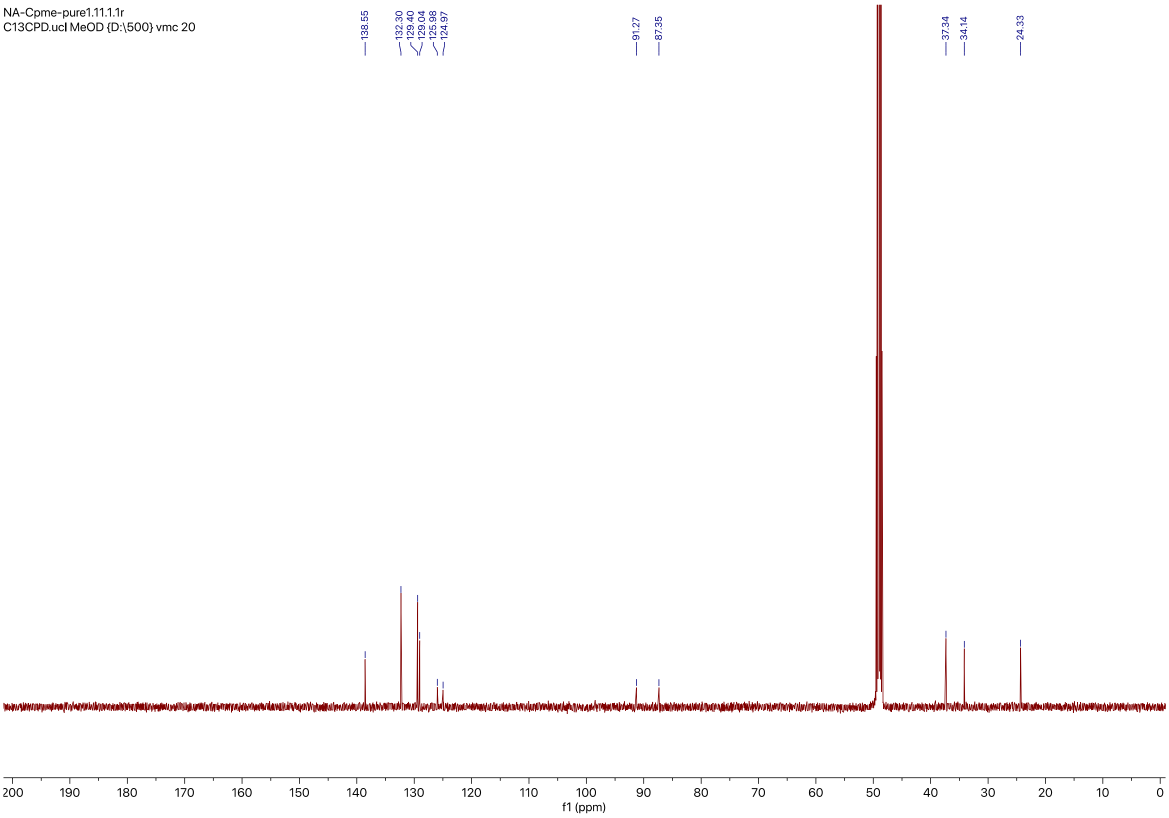
NA-Cpme-pure1.11.1.1r
C13CPD.ucl MeOD {D:\500} vmc 20
138.55
132.30
129.40
129.04
125.98
124.97
91.27
87.35
37.34
34.14
24.33
f1 (ppm)

General Procedure for aerobic C-H functionalisation of THF – Method B (Alkynylation)

To a solution of THF (1.25 mmol, 5.0 eq.) in HFIP (0.40 mmol) was added the respective acetylenic triflone **6x** (60 mg, 0.25 mmol, 1.0 eq). The reaction mixture was stirred at 80 °C for 16 h and then poured over sat. aq. $NaHCO_3$ (5 mL). The organics were extracted with EtOAc (3 × 10 mL), dried over $MgSO_4$, filtered and the solvent removed under reduced pressure. The resultant crude residue was purified as described below.

2-((4-Methoxyphenyl)ethynyl)tetrahydrofuran – 9a

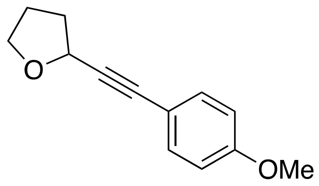

Following application of method B, purification by column chromatography (0-15% EtOAc/cyclohexane) afforded 2-((4-methoxyphenyl)ethynyl)tetrahydrofuran **9a** as a yellow oil (35 mg, 0.17 mmol, 69%). ^{1}H NMR (400 MHz, $CDCl_3$) δ 7.41–7.32 (m, 2H), 6.86–6.77 (m, 2H), 4.79 (dd, *J* = 7.1, 5.2 Hz, 1H), 4.06–3.96 (m, 1H), 3.85 (td, *J* = 8.0, 5.5 Hz, 1H), 3.80 (s, 3H), 2.36–2.17 (m, 1H), 2.14–2.00 (m, 2H), 1.99–1.85 (m, 1H); ^{13}C NMR (126 MHz, $CDCl_3$) δ 159.7 (C), 133.3 (CH), 115.1 (C), 114.0 (CH), 87.8 (C), 84.5 (C), 68.8 (CH_2), 68.0 (CH), 55.4 (CH_3), 33.6 (CH_2), 25.6 (CH_2); IR (thin film): 2930, 2903, 2234, 1568, 1501 cm^{-1}. Known compound.[2]

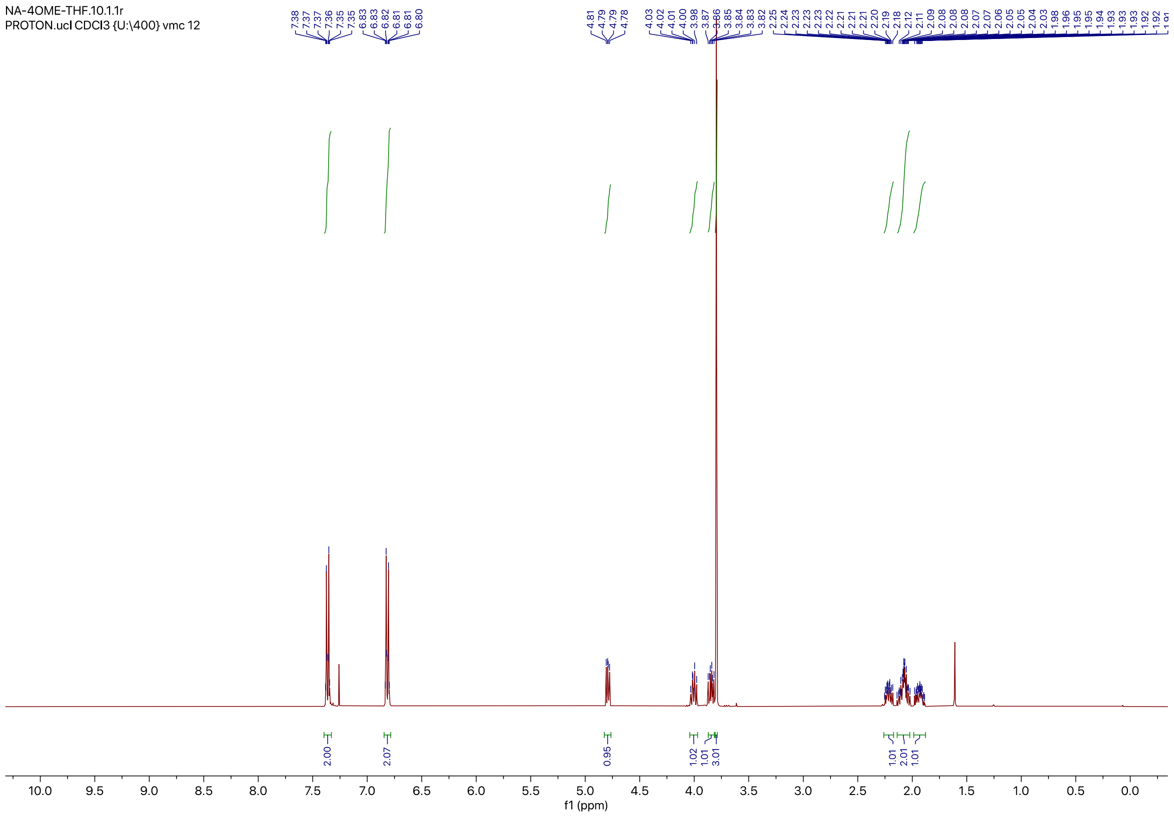

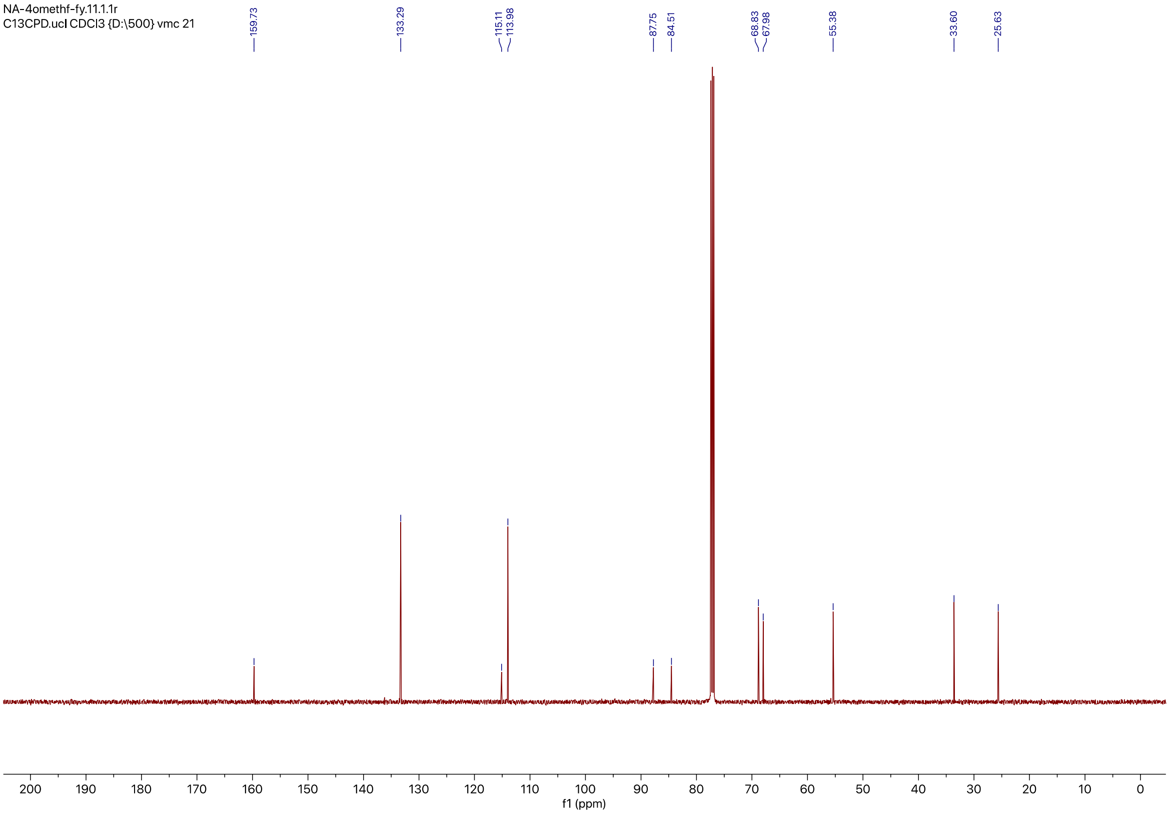
NA-4omethf-fy.11.1.1r
C13CPD.ucl CDCl3 {D:\500} vmc 21
159.73
133.29
115.11
113.98
87.75
84.51
68.83
67.98
55.38
33.60
25.63
200 190 180 170 160 150 140 130 120 110 100 90 80 70 60 50 40 30 20 10 0
f1 (ppm)

2-((4-Ethylphenyl)ethynyl)tetrahydrofuran – 10a

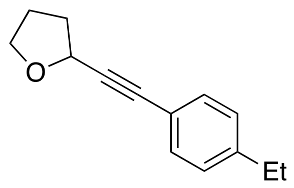

Following application of method B, purification by column chromatography (0-15% EtOAc/cyclohexane) afforded 2-((4-ethylphenyl)ethynyl)tetrahydrofuran **10a** as a yellow oil (39 mg, 0.23 mmol, 90%). ^{1}H NMR (400 MHz, $CDCl_3$) δ 7.41–7.31 (m, 2H), 7.12 (d, *J* = 8.4 Hz, 2H), 4.81 (dd, *J* = 7.1, 5.0 Hz, 1H), 4.06–3.96 (m, 1H), 3.90–3.80 (m, 1H), 2.63 (q, *J* = 7.6 Hz, 2H), 2.27–2.17 (m, 1H), 2.14–2.01 (m, 2H), 1.99–1.89 (m, 1H), 1.22 (t, *J* = 7.6 Hz, 3H); ^{13}C NMR (126 MHz, $CDCl_3$) δ 144.8 (C), 131.8 (CH), 127.9 (CH), 120.1 (C), 88.4 (C), 84.8 (C), 68.8 (CH), 68.0 (CH_2), 33.6 (CH_2), 28.9 (CH_2), 25.6 (CH_2), 15.5 (CH_3); IR (thin film): 2942, 2913, 2217, 1558, 1511 cm^{-1}. Known compound.[9]

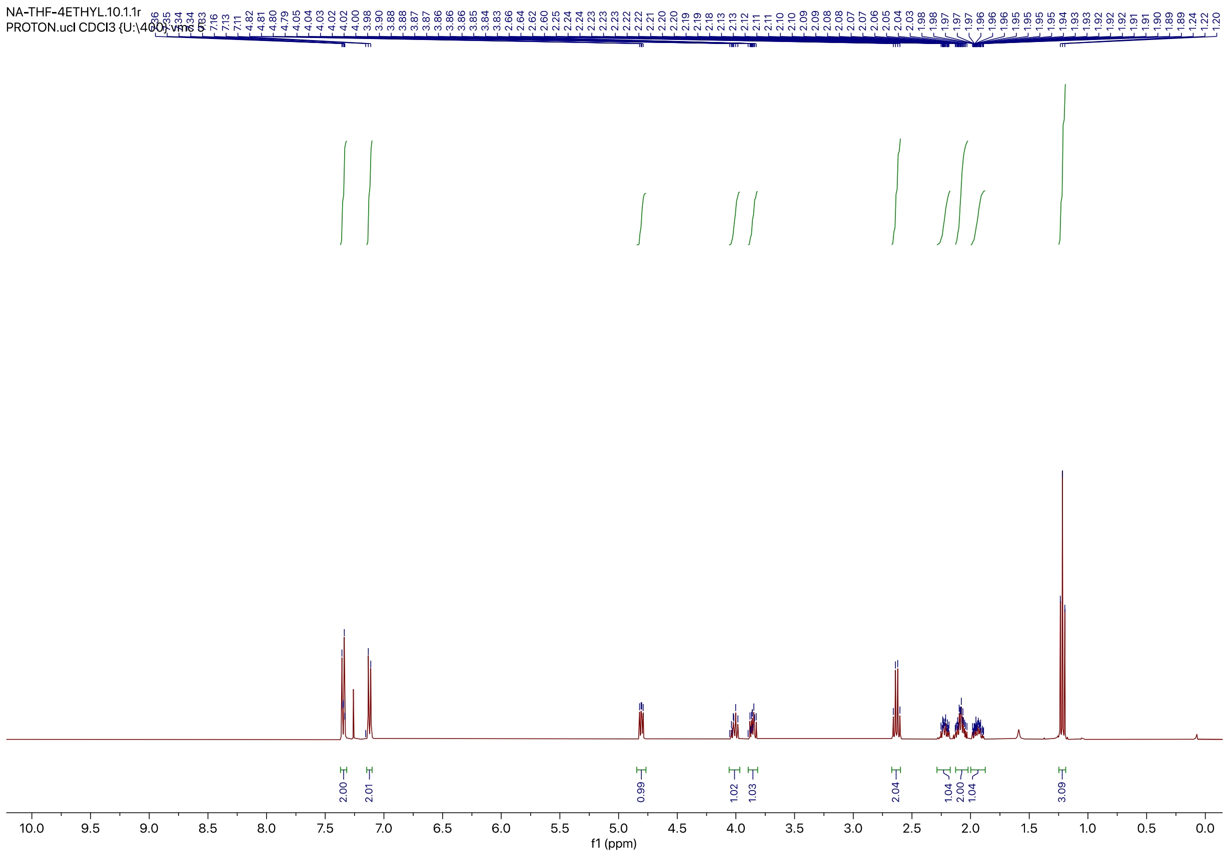

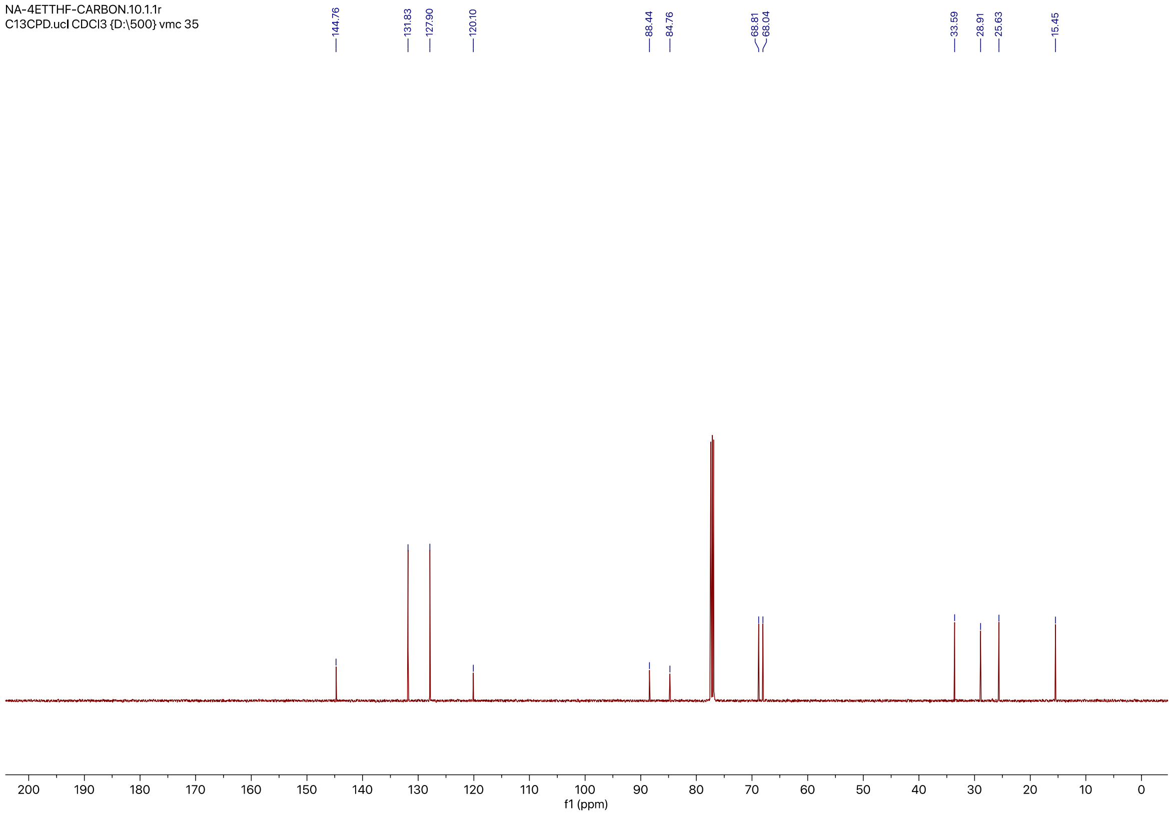
NA-4ETTHF-CARBON.10.1.1r
C13CPD.ucl CDCl3 {D:\500} vmc 35
144.76
131.83
127.90
120.10
88.44
84.76
68.81
68.04
33.59
28.91
25.63
15.45
200 190 180 170 160 150 140 130 120 110 100 90 80 70 60 50 40 30 20 10 0
f1 (ppm)

2-((4-Pentylphenyl)ethynyl)tetrahydrofuran – 11a

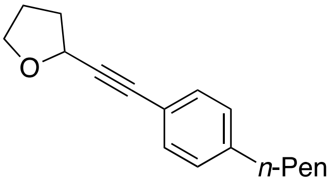

Following application of method B, purification by column chromatography (0-15% EtOAc/cyclohexane) afforded 2-((4-pentylphenyl)ethynyl)tetrahydrofuran **11a** as a clear oil (51 mg, 0.21 mmol, 84%). ^{1}H NMR (600 MHz, $CDCl_3$) δ 7.34 (d, J = 8.2 Hz, 2H), 7.10 (d, J = 8.3 Hz, 2H), 4.80 (dd, J = 7.2, 5.1 Hz, 1H), 4.08–3.96 (m, 1H), 3.85 (td, J = 8.0, 5.5 Hz, 1H), 2.60–2.54 (m, 2H), 2.28–2.17 (m, 1H), 2.13–2.03 (m, 2H), 1.98–1.87 (m, 1H), 1.58 (tt, J = 15.0, 7.4 Hz, 3H), 1.30 (ttd, J = 15.4, 8.2, 2.3 Hz, 5H), 0.88 (t, J = 7.1 Hz, 3H). ^{13}C NMR (151 MHz, $CDCl_3$) δ 143.5 (C), 131.7 (CH), 128.5 (CH), 120.0 (C), 88.4 (C), 84.8 (C), 68.8 (CH), 68.0 (CH_2), 35.9 (CH_2), 33.6 (CH_2), 31.6 (CH_2), 31.1 (CH_2), 27.1 (CH_2), 25.6 (CH_2), 22.7 (CH_2), 14.2 (CH_3); IR (thin film): 2934, 2911, 2902, 2205, 1560, 1512 cm^{-1}. Known compound.[10]

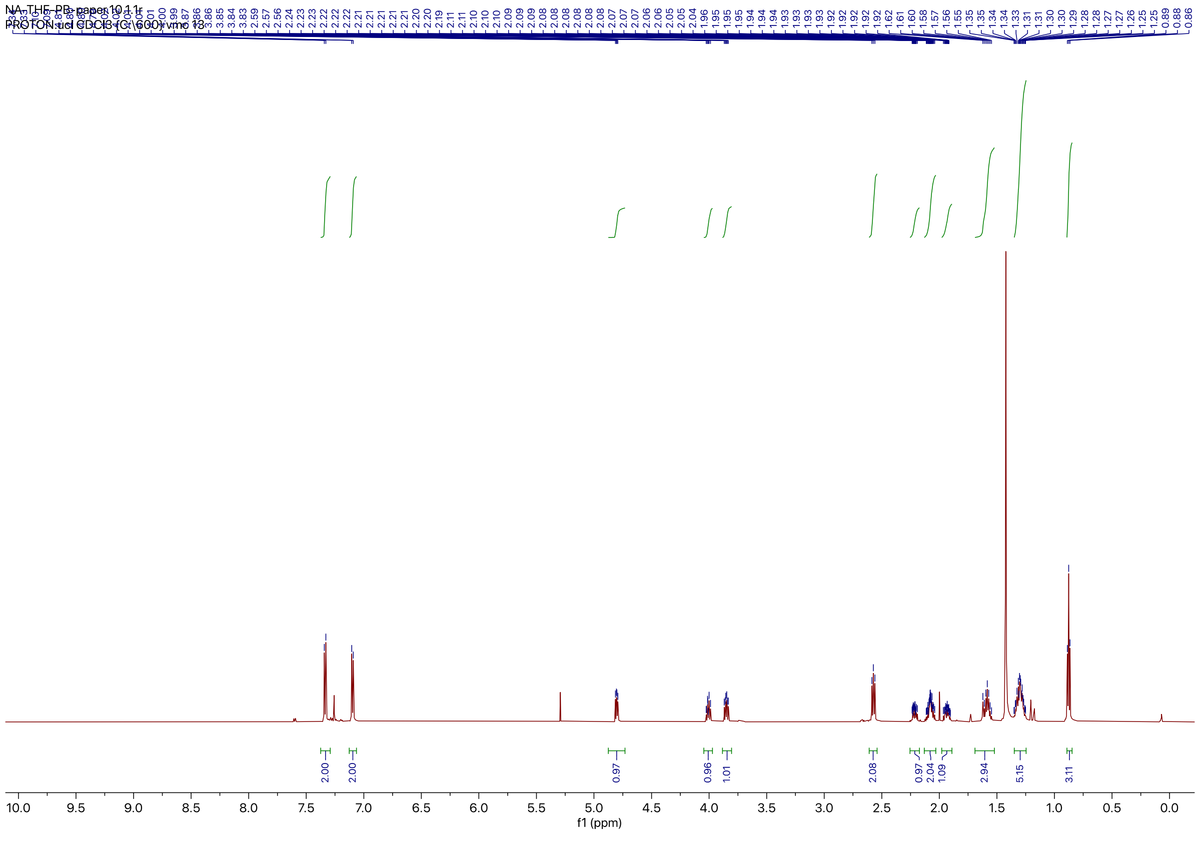

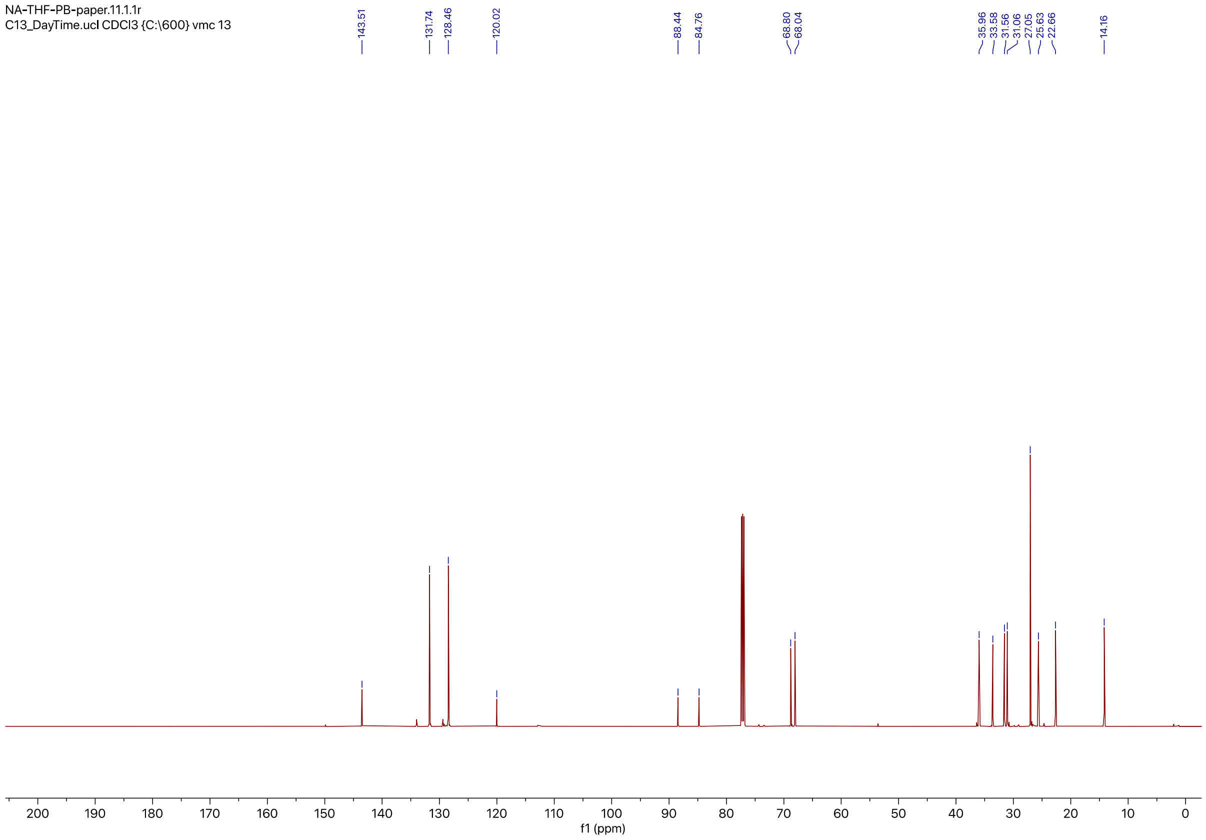
NA-THF-PB-paper.11.1.1r
C13_DayTime.ucl CDCl3 {C:\600} vmc 13
143.51
131.74
128.46
120.02
88.44
84.76
68.80
68.04
35.96
33.58
31.56
31.06
27.05
25.63
22.66
14.16
200
190
180
170
160
150
140
130
120
110
100
90
80
70
60
50
40
30
20
10
0
f1 (ppm)

2-(*M*-tolylethynyl)tetrahydrofuran – 12a

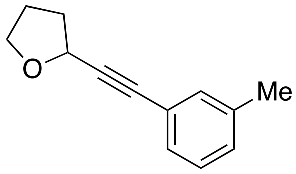

Following application of method B, purification by column chromatography (0-15% EtOAc/cyclohexane) afforded 2-(*m*-tolylethynyl)tetrahydrofuran **12a** as a yellow oil (38 mg, 0.20 mmol, 80%). ^{1}H NMR (400 MHz, $CDCl_3$) δ 7.28–7.22 (m, 3H), 7.18 (t, J = 7.5 Hz, 1H), 7.11 (d, J = 7.7 Hz, 1H), 4.81 (dd, J = 7.2, 5.0 Hz, 1H), 4.07–3.94 (m, 1H), 3.92–3.79 (m, 1H), 2.31 (s, 3H), 2.27–2.18 (m, 1H), 2.09-2.02 (m, 2H), 2.00–1.90 (m, 1H). ^{13}C NMR (126 MHz, $CDCl_3$) δ 138.0 (C), 132.5 (CH), 129.3 (CH), 128.9 (CH), 128.2 (CH), 122.8 (C), 88.8 (C), 84.8 (C), 68.8 (CH), 68.1 (CH_2), 33.6 (CH_2), 25.6 (CH_2), 21.3 (CH_3). IR (thin film): 2911, 2902, 2213, 1559, 1512 cm^{-1}. Known compound.[2]

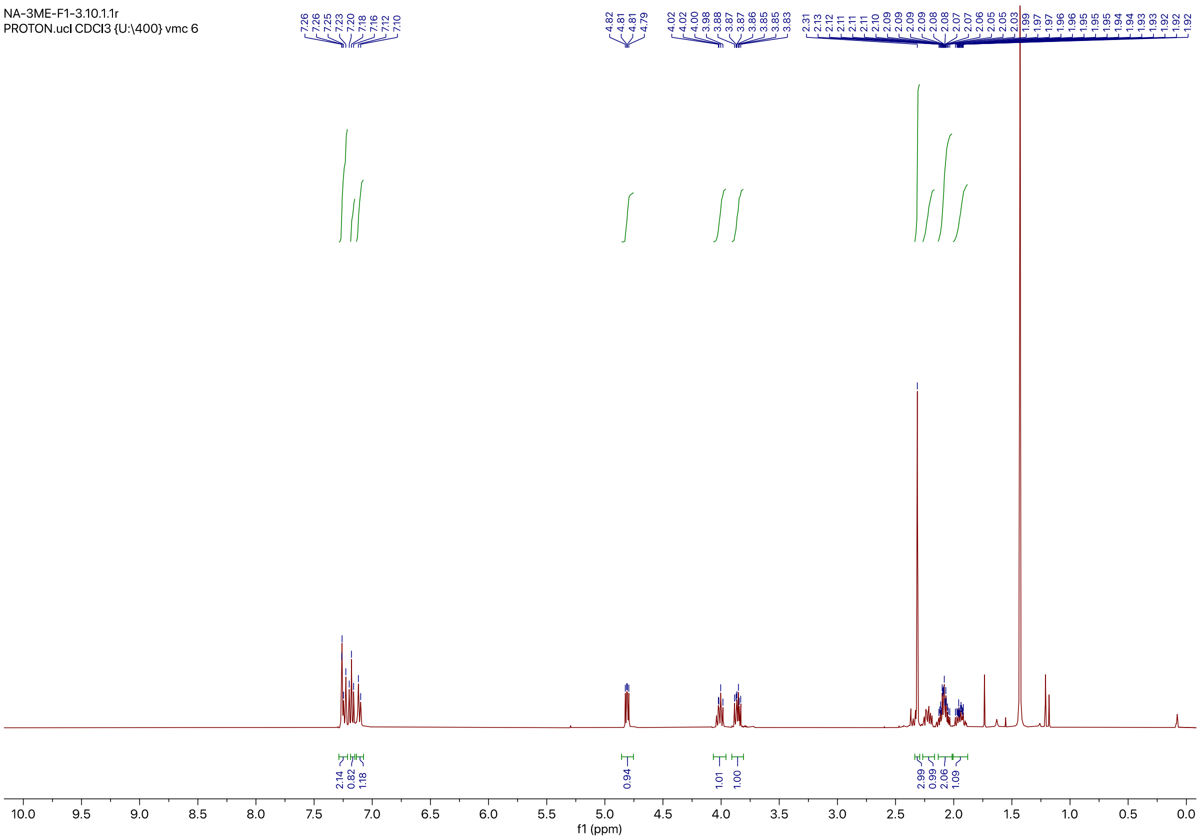

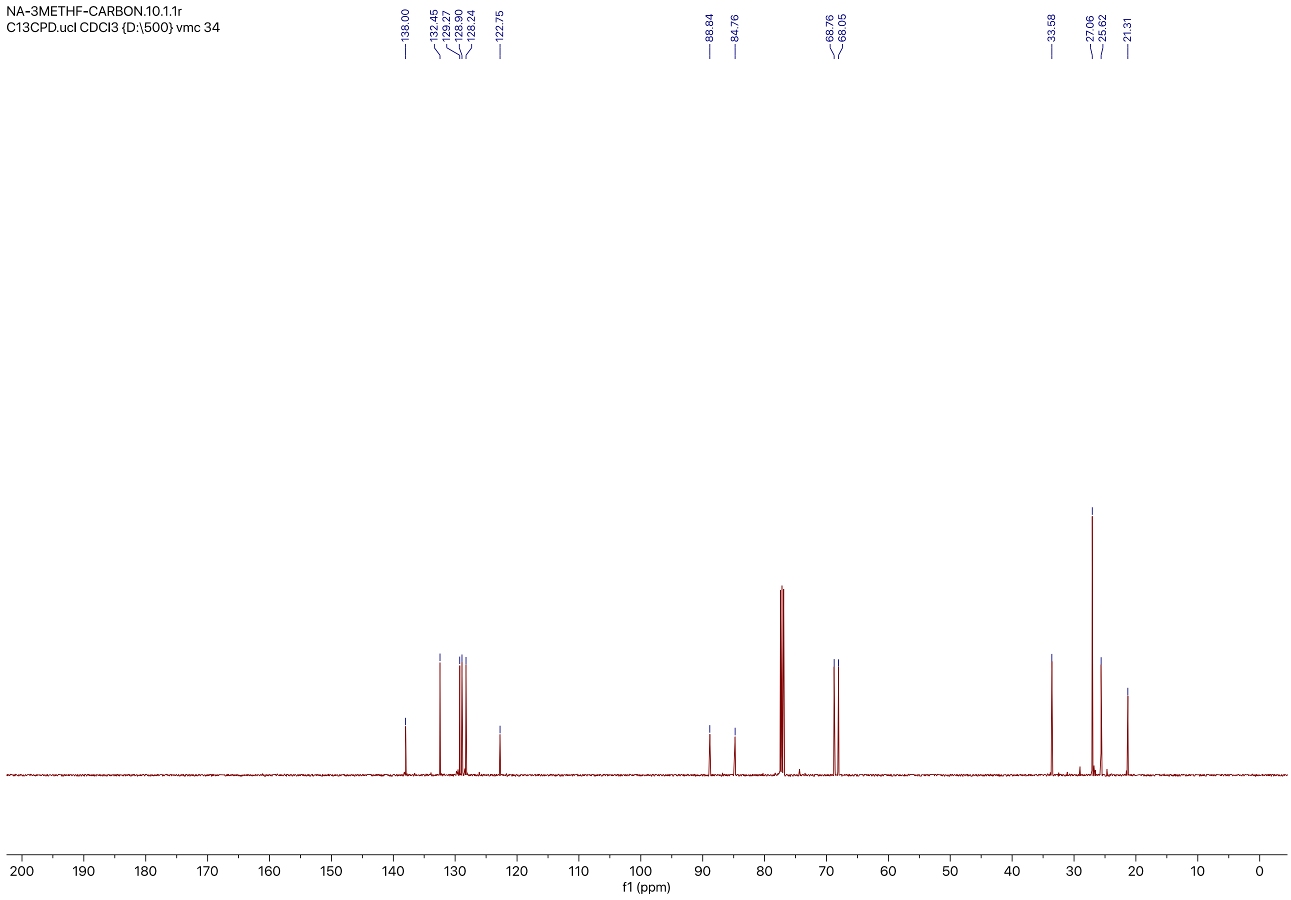

NA-3METHF-CARBON.10.1.1r
C13CPD.ucl CDCl3 {D:\500} vmc 34
138.00
132.45
129.27
128.90
128.24
122.75
88.84
84.76
68.76
68.05
33.58
27.06
25.62
21.31
200 190 180 170 160 150 140 130 120 110 100 90 80 70 60 50 40 30 20 10 0
f1 (ppm)

2-((4-Bromophenyl)ethynyl)tetrahydrofuran – 13a

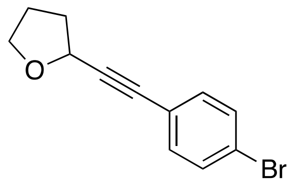

Following application of method B, purification by column chromatography (0-15% EtOAc/cyclohexane) afforded 2-((4-bromophenyl)ethynyl)tetrahydrofuran **13a** as a yellow oil (60 mg, 0.24 mmol, 96%). ^{1}H NMR (500 MHz, $CDCl_3$) δ 7.45–7.38 (m, 2H), 7.33–7.26 (m, 2H), 4.78 (dd, J = 7.2, 5.1 Hz, 1H), 4.05–3.94 (m, 1H), 3.85 (ddd, J = 8.3, 7.7, 5.5 Hz, 1H), 2.27–2.18 (m, 1H), 2.14–2.01 (m, 2H), 2.00–1.89 (m, 1H); ^{13}C NMR (126 MHz, $CDCl_3$) δ 133.3 (CH), 131.6 (CH), 122.7 (C), 121.9 (C), 90.4 (C), 83.6 (C), 68.7 (CH_2), 68.2 (CH), 33.5 (CH_2), 25.7 (CH_2); IR (thin film): 2929, 2909, 2215, 1540, 1522 cm^{-1}. Known compound.[10]

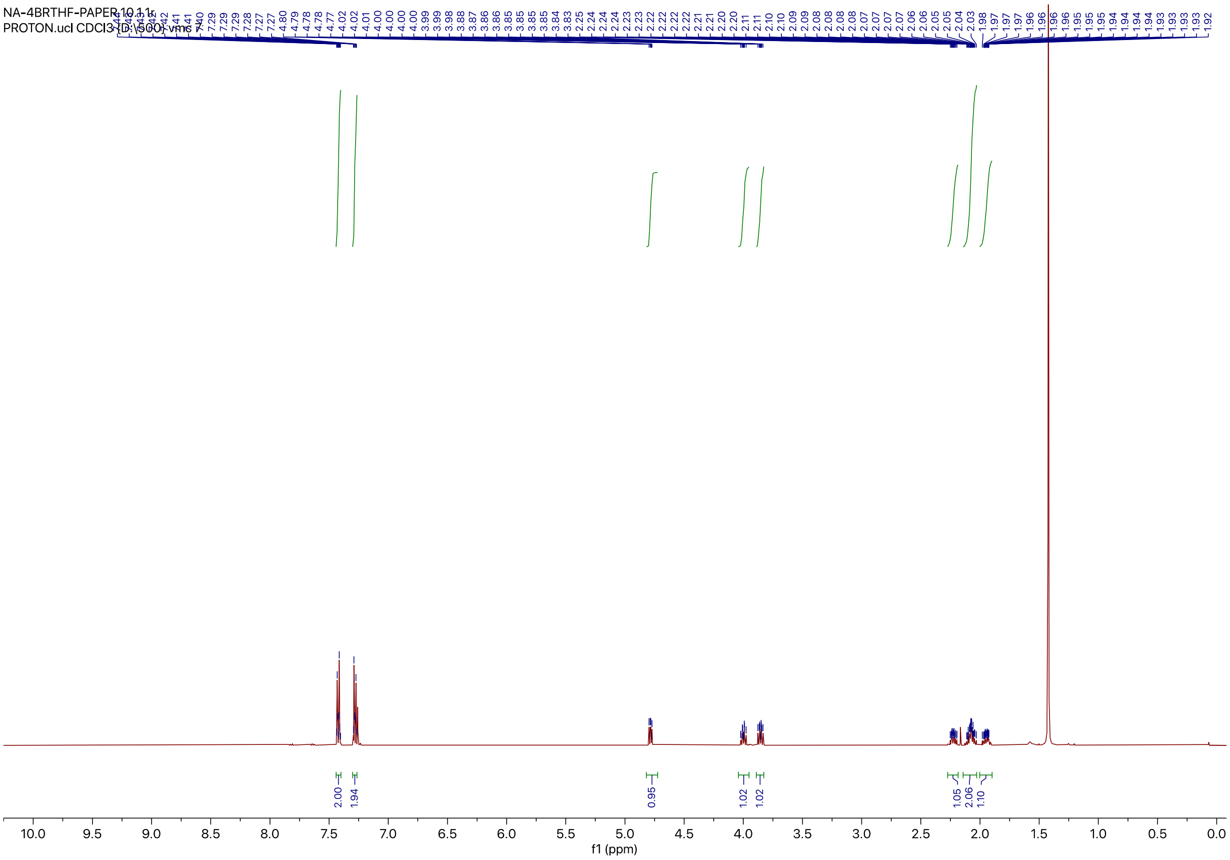

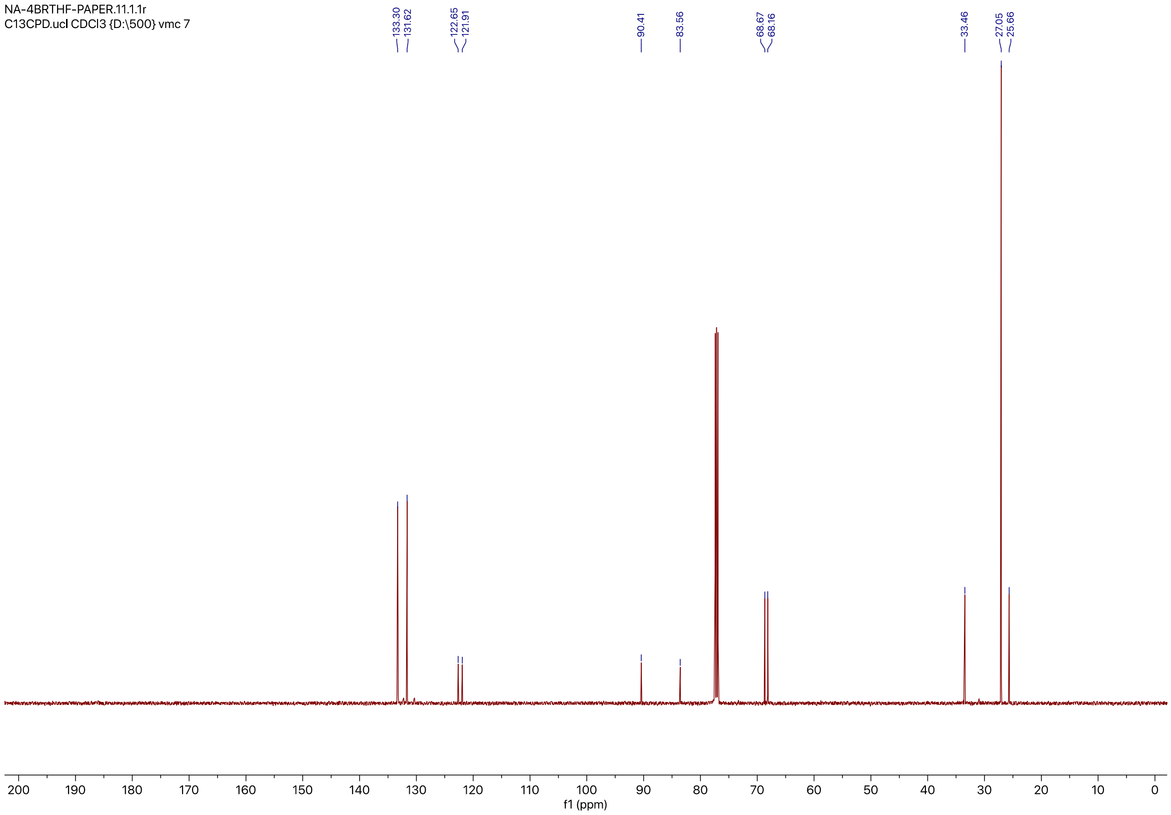
NA-4BRTHF-PAPER.11.1.1r
C13CPD.ucl CDCl3 {D:\500} vmc 7
133.30
131.62
122.65
121.91
90.41
83.56
68.67
68.16
33.46
27.05
25.66
200
190
180
170
160
150
140
130
120
110
100
90
80
70
60
50
40
30
20
10
0
f1 (ppm)

2-((3-Fluorophenyl)ethynyl)tetrahydrofuran – 14a

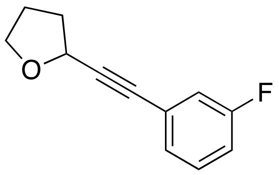

Following application of method B, purification by column chromatography (0-15% EtOAc/cyclohexane) afforded 2-((3-fluorophenyl)ethynyl)tetrahydrofuran **14a** as a yellow oil (36 mg, 0.19 mmol, 76%). ^{1}H NMR (400 MHz, $CDCl_3$) δ 7.29–7.18 (m, 2H), 7.12 (ddd, *J* = 9.5, 2.5,1.4 Hz, 1H), 7.05–6.96 (m, 1H), 4.80 (dd, *J* = 7.2, 5.0 Hz, 1H), 4.06–3.94 (m, 1H), 3.92–3.81 (m, 1H), 2.31–2.17 (m, 1H), 2.14–2.03 (m, 2H), 2.01–1.89 (m, 1H); ^{13}C NMR (126 MHz, $CDCl_3$) δ ^{13}C NMR (126 MHz, $CDCl_3$) δ 162.4 (d, *J* = 246.4 Hz), 129.9 (d, *J* = 8.6 Hz), 127.7 (d, *J* = 3.0 Hz), 124.8 (d, *J* = 9.4 Hz), 118.6 (d, *J* = 22.8 Hz), 115.7 (d, *J* = 21.2 Hz), 90.3 (C), 83.4 (C), 68.6 (CH_2), 68.1 (CH), 33.5 (CH_2), 25.6 (CH_2). IR (thin film): 2930, 2230, 1555, 1512 cm^{-1}. Known compound.[2]

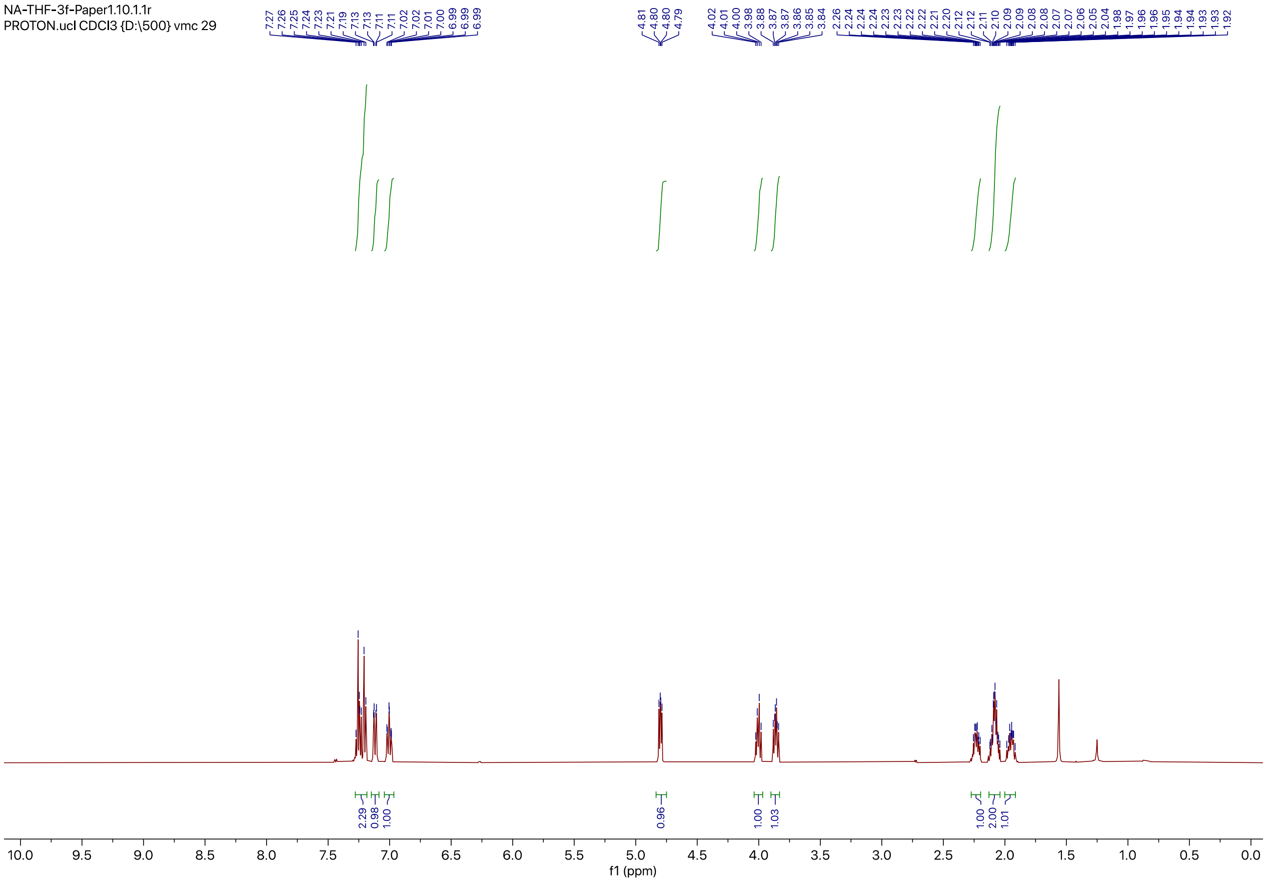

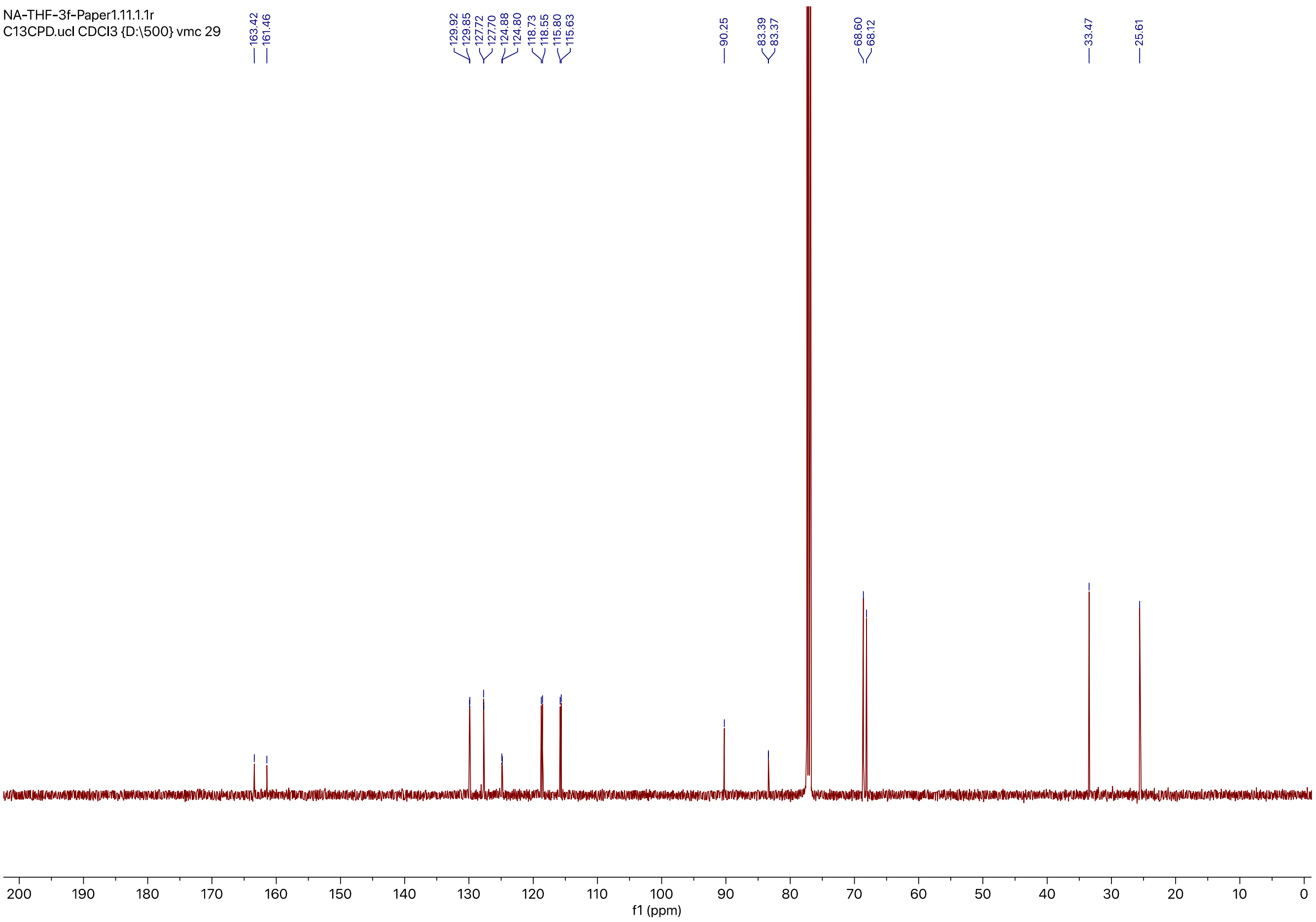
NA-THF-3f-Paper1.11.1.1r
C13CPD.ucl CDCl3 {D:\500} vmc 29
163.42
161.46
129.92
129.85
127.72
127.70
124.88
124.80
118.73
118.55
115.80
115.63
90.25
83.39
83.37
68.60
68.12
33.47
25.61
f1 (ppm)

2-((2-Chlorophenyl)ethynyl)tetrahydrofuran – 15a

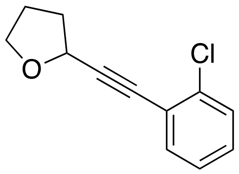

Following application of method B, purification by column chromatography (0-15% EtOAc/cyclohexane) afforded 2-((2-chlorophenyl)ethynyl)tetrahydrofuran **15a** as a yellow oil (37 mg, 0.18 mmol, 73%). ^{1}H NMR (600 MHz, $CDCl_3$) δ 7.45 (dd, J = 7.6, 1.8 Hz, 1H), 7.37 (dd, J = 8.0, 1.2 Hz, 1H), 7.22 (td, J = 7.7, 1.8 Hz, 1H), 7.18 (td, J = 7.5, 1.3 Hz, 1H), 4.87 (dd, J = 7.4, 4.5 Hz, 1H), 4.08–3.97 (m, 1H), 3.88 (td, J = 8.2, 5.6 Hz, 1H), 2.28–2.19 (m, 1H), 2.17 –2.08 (m, 2H), 1.99–1.90 (m, 1H). ^{13}C NMR (151 MHz, $CDCl_3$) δ 136.1 (C), 133.5 (CH), 129.5 (CH), 129.3 (CH), 126.5 (CH), 122.8 (C), 94.7 (C), 81.4 (C), 68.7 (CH_2), 68.1 (CH), 33.5 (CH_2), 25.5 (CH_2); IR (thin film): 2929, 2910, 2214, 1543, 1502 cm^{-1}. Known compound.[9]

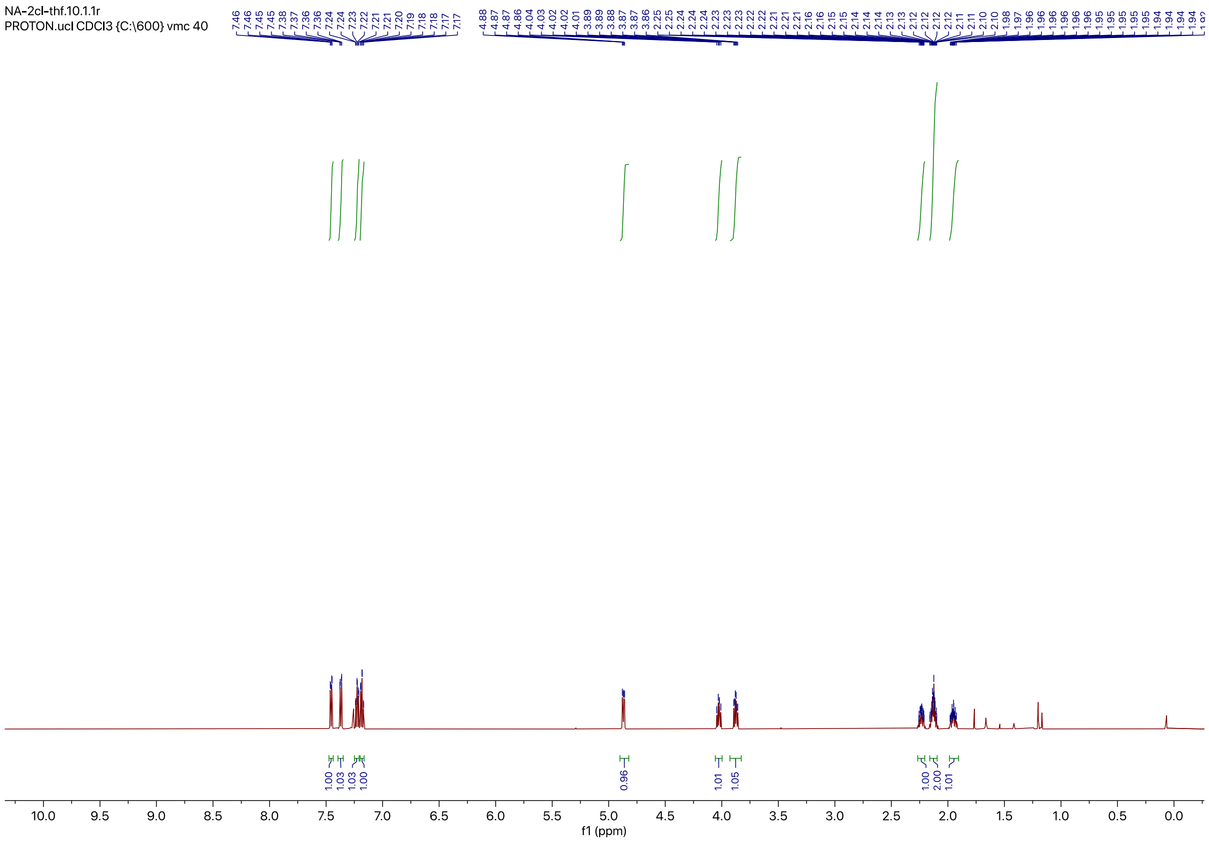

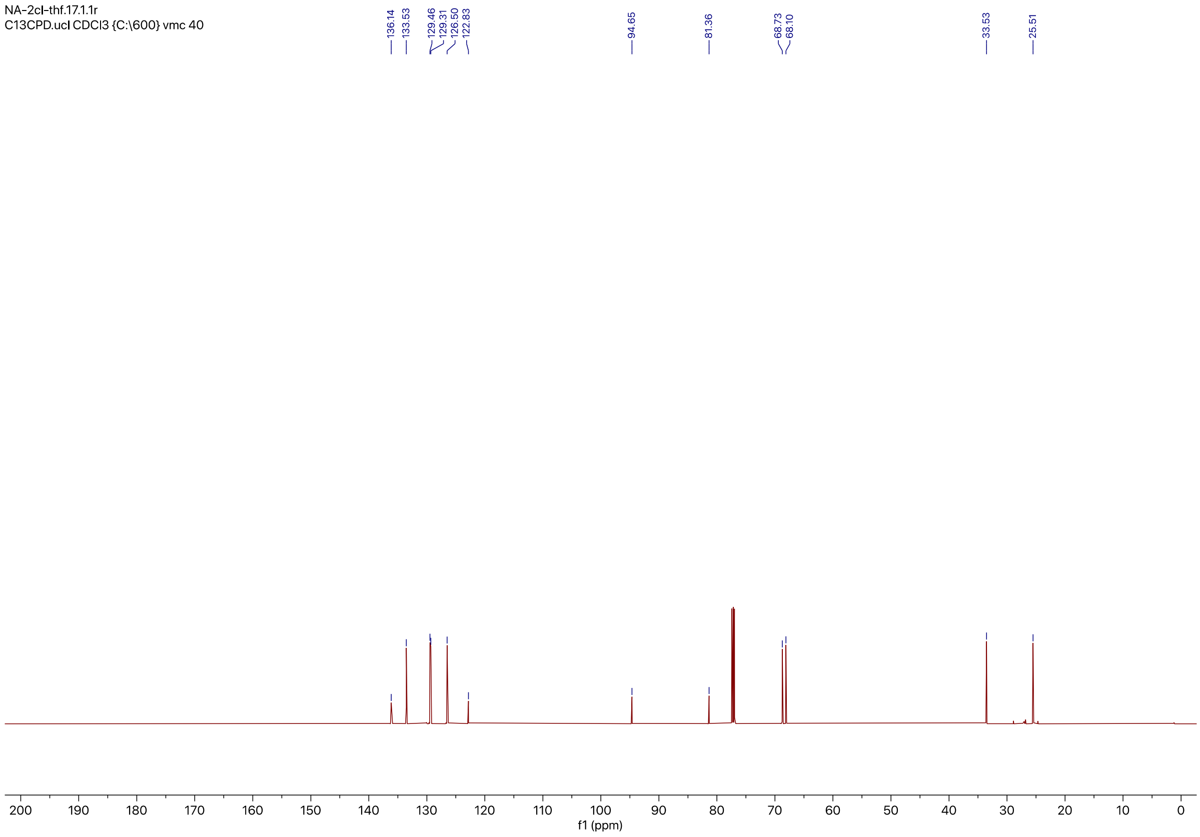
NA-2cl-thf.17.1.1r
C13CPD.ucl CDCl3 {C:\600} vmc 40
136.14
133.53
129.46
129.31
126.50
122.83
94.65
81.36
68.73
68.10
33.53
25.51
200
190
180
170
160
150
140
130
120
110
100
90
80
70
60
50
40
30
20
10
0
f1 (ppm)

2-((4-(Trifluoromethyl)phenyl)ethynyl)tetrahydrofuran – 16a

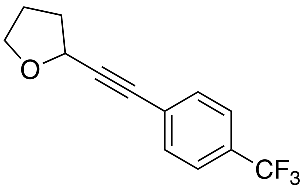

Following application of method B, purification by column chromatography (0-15% EtOAc/cyclohexane) afforded 2-((4-(trifluoromethyl)phenyl)ethynyl)tetrahydrofuran **16a** as a yellow oil (52 mg, 0.22 mmol, 88%). ^{1}H NMR (600 MHz, $CDCl_3$) δ 7.56–7.54 (m, 2H), 7.53–7.51 (m, 2H), 4.81 (dd, *J* = 7.3, 5.0 Hz, 1H), 4.05–3.94 (m, 1H), 3.87 (td, *J* = 7.8, 5.5 Hz, 1H), 2.31–2.17 (m, 1H), 2.15–2.02 (m, 2H), 1.99–1.88 (m, 1H). ^{13}C NMR (151 MHz, $CDCl_3$) δ 132.1 (CH), 130.0 (q, C, $J_{F,C}$ = 32.7 Hz), 126.8 (C), 125.3 (q, CH, $J_{F,C}$ = 3.7 Hz) , 124.0 (d, CH, $J_{F,C}$ = 272.2 Hz), 121.3 (CH), 91.8 (C), 83.3 (C), 68.6 (CH_2), 68.3 (CH), 33.4 (CH_2), 25.7 (CH_2); IR (thin film): 2949, 2907, 2222, 1543, 1502 cm^{-1}. Known compound.[2]

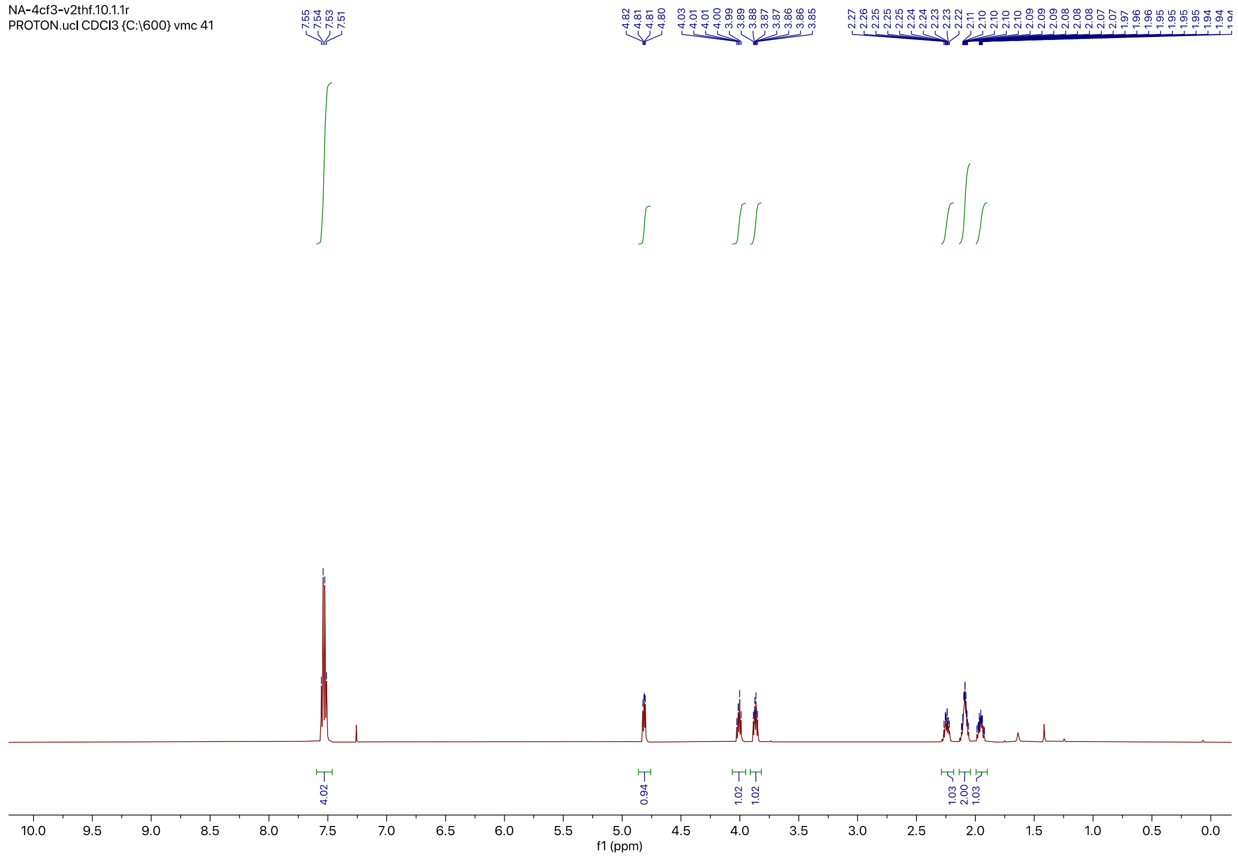

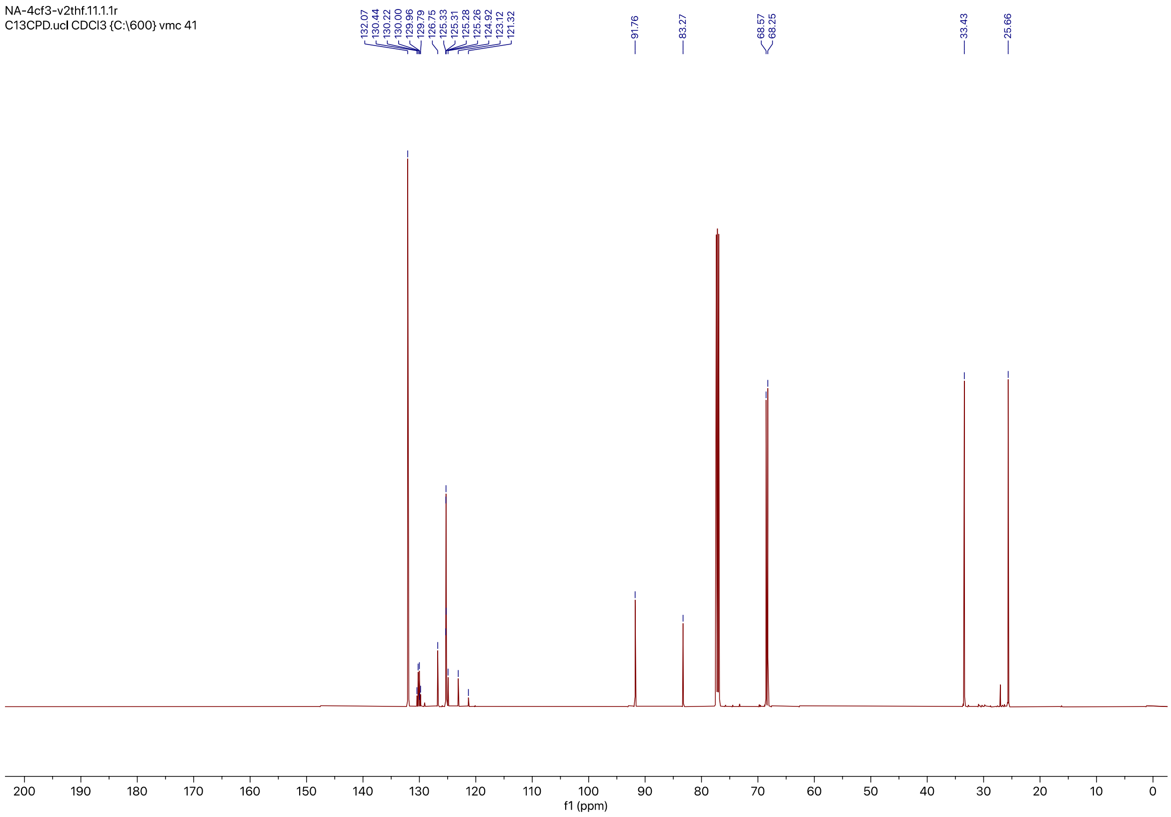

NA-4cf3-v2thf.11.1.1r
C13CPD.ucl CDCl3 {C:\600} vmc 41
132.07
130.44
130.22
130.00
129.96
129.79
126.75
125.33
125.31
125.28
125.26
124.92
123.12
121.32
91.76
83.27
68.57
68.25
33.43
25.66
200 190 180 170 160 150 140 130 120 110 100 90 80 70 60 50 40 30 20 10 0
f1 (ppm)

2-(Thiophen-2-ylethynyl)tetrahydrofuran – 17a

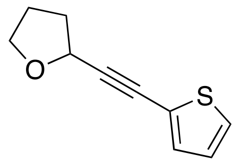

Following application of method B, purification by column chromatography (0-15% EtOAc/cyclohexane) afforded 2-(thiophen-2-ylethynyl)tetrahydrofuran **17a** as a brown oil (32 mg, 0.18 mmol, 72%). ^{1}H NMR (500 MHz, $CDCl_3$) δ 7.29–7.13 (m, 2H), 6.94 (dd, *J* = 5.1, 3.6 Hz, 1H), 4.82 (dd, *J* = 7.3, 4.9 Hz, 1H), 4.07–3.94 (m, 1H), 3.85 (td, *J* = 7.9, 5.5 Hz, 1H), 2.28–2.16 (m, 1H), 2.14–2.04 (m, 2H), 1.99–1.89 (m, 1H). ^{13}C NMR (126 MHz, $CDCl_3$) δ 132.3 (CH), 127.2 (CH), 127.0 (CH), 122.9 (C), 93.1 (C), 77.9 (C), 68.8 (CH_2), 68.2 (CH), 33.4 (CH_2), 25.7 (CH_2); IR (thin film): 3067, 2926, 2209, 1518, 1463 cm^{-1}. Known compound.[10]

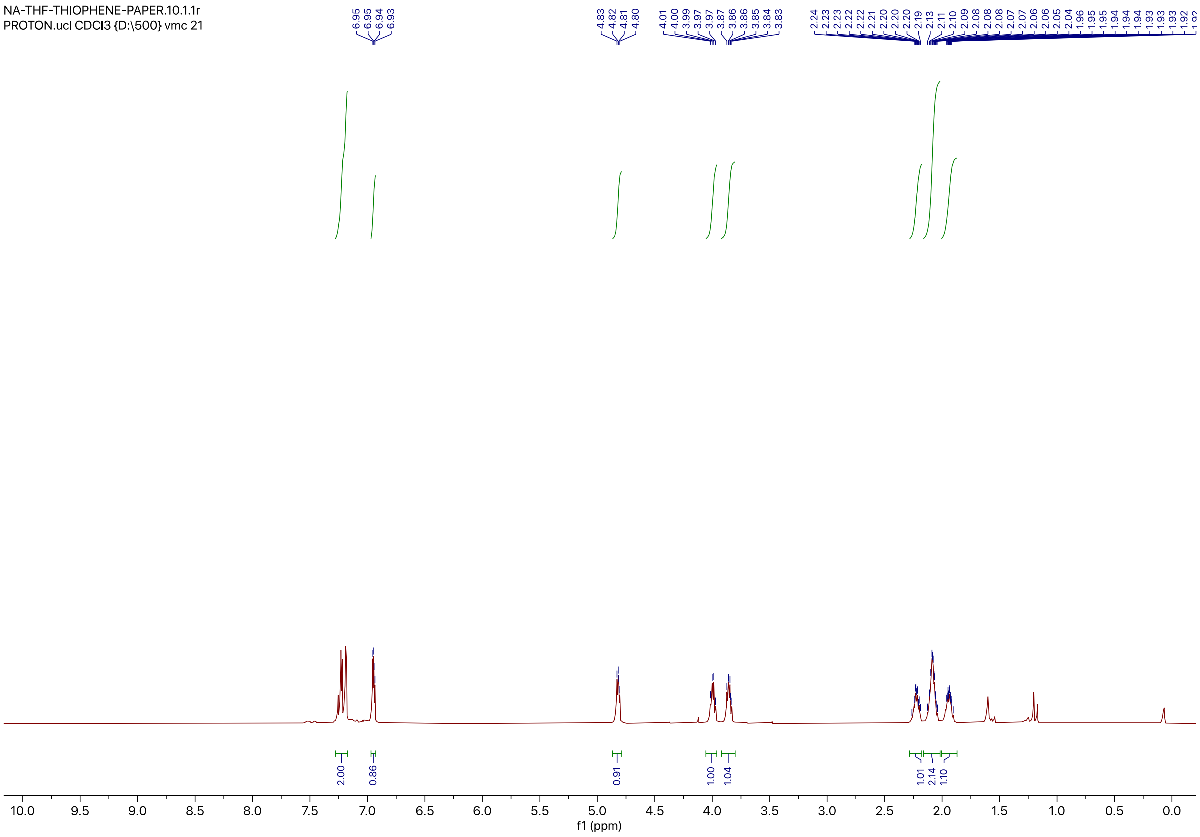

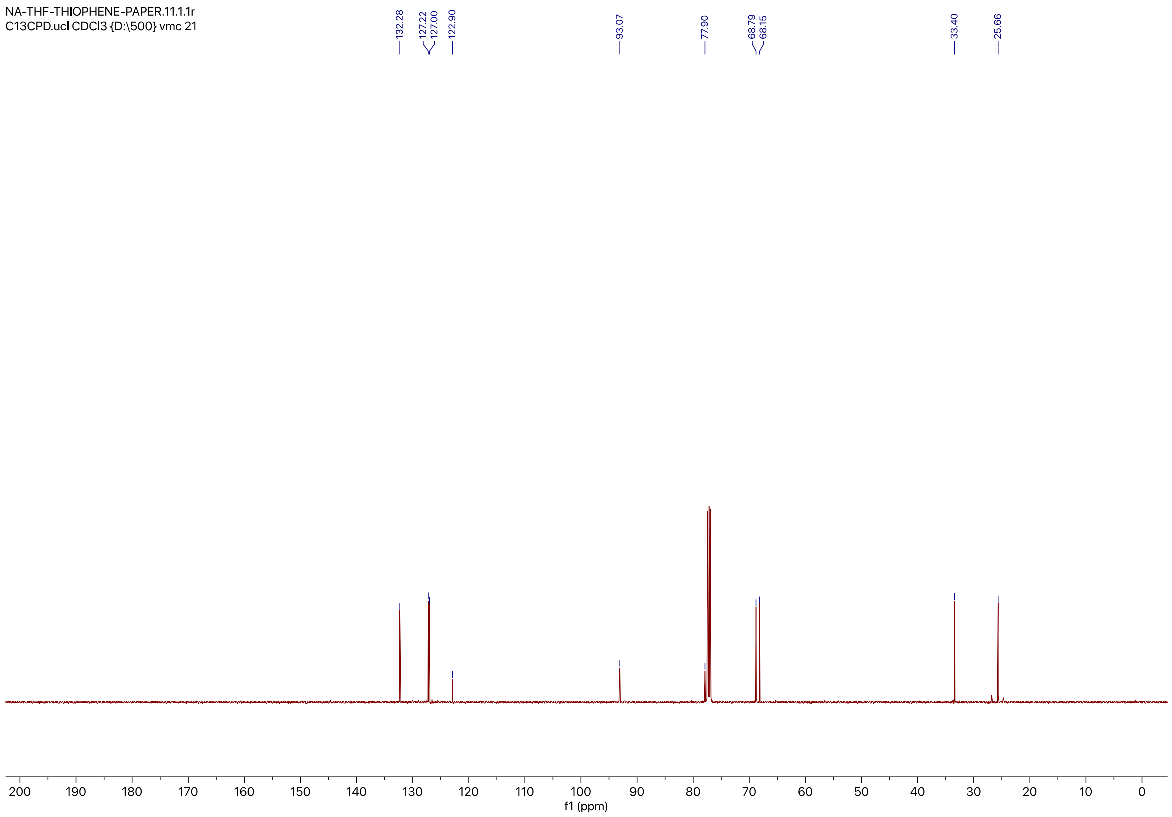
NA-THF-THIOPHENE-PAPER.11.1.1r
C13CPD.ucl CDCl3 {D:\500} vmc 21
132.28
127.22
127.00
122.90
93.07
77.90
68.79
68.15
33.40
25.66
200 190 180 170 160 150 140 130 120 110 100 90 80 70 60 50 40 30 20 10 0
f1 (ppm)

2-((6-Methoxynaphthalen-2-yl)ethynyl)tetrahydrofuran – 18a

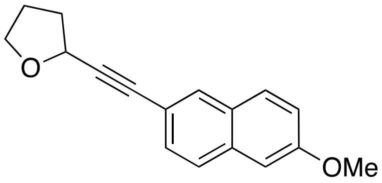

Following application of method B, purification by column chromatography (0-15% EtOAc/cyclohexane) afforded 2-((6-methoxynaphthalen-2-yl)ethynyl)tetrahydrofuran **18a** as a yellow oil (41 mg, 0.16 mmol, 65%). ^{1}H NMR (500 MHz, $CDCl_3$) δ 7.88 (s, 1H), 7.67 (d, *J* = 9.0 Hz, 1H), 7.64 (d, *J* = 8.7 Hz, 1H), 7.44 (dd, *J* = 8.5, 1.6 Hz, 1H), 7.14 (dd, *J* = 8.9, 2.5 Hz, 1H), 7.09 (d, *J* = 2.5 Hz, 1H), 4.85 (dd, *J* = 7.2, 5.1 Hz, 1H), 4.08–4.00 (m, 1H), 3.91 (s, 3H), 3.96–3.84 (m, 1H), 2.33–2.20 (m, 1H), 2.17–2.07 (m, 2H), 2.01–1.91 (m, 1H). ^{13}C NMR (126 MHz, $CDCl_3$) δ ^{13}C NMR (126 MHz, $CDCl_3$) δ 134.9 (C), 134.3 (C), 131.6 (CH), 129.4 (CH), 129.3 (CH), 128.5 (CH), 126.8 (CH), 119.5 (C), 105.9 (C), 88.8 (C), 68.9 (CH_2), 68.1 (CH), 55.5 (CH_3), 33.6 (CH_2), 27.1 (CH_2), 25.7 (CH); IR (thin film): 3012, 2930, 2199, 1555, 1503 cm^{-1}. HRMS (ESI) calcd for $C_{17}H_{16}O_2$ $[M+H]^+$ 253.1223; observed 253.1199.

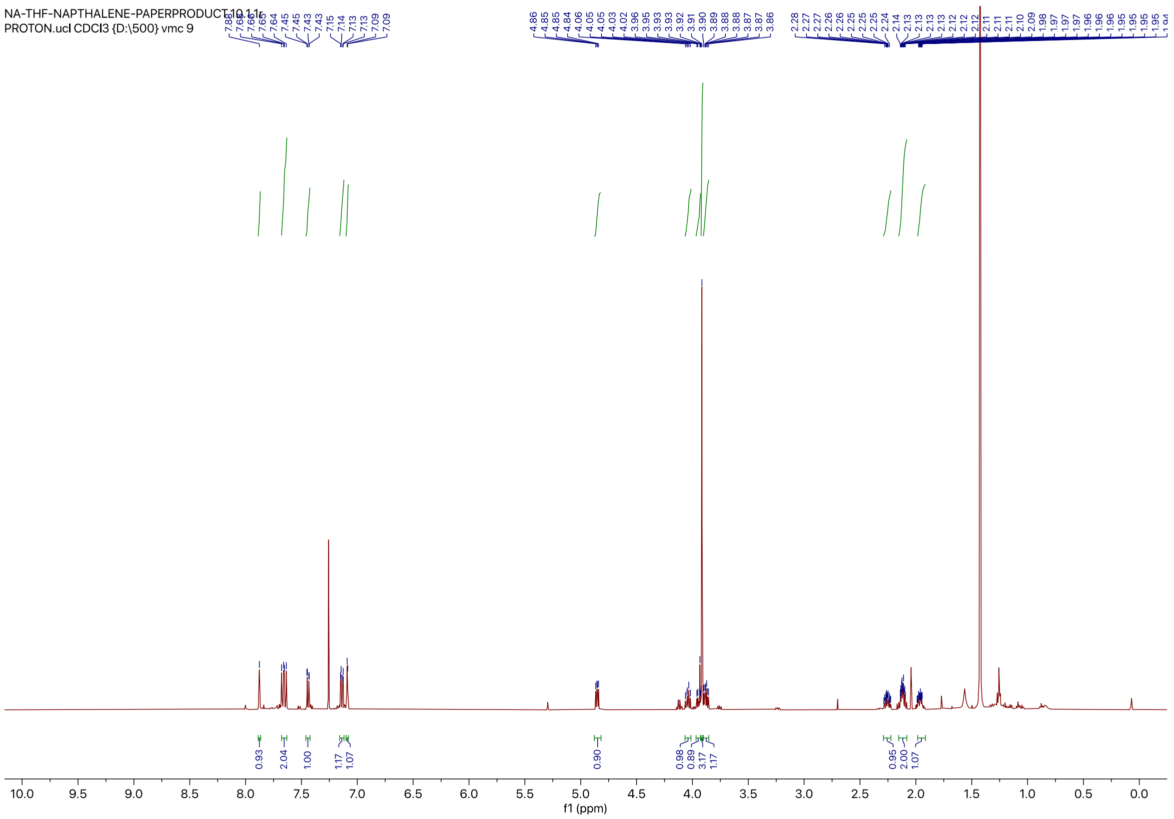

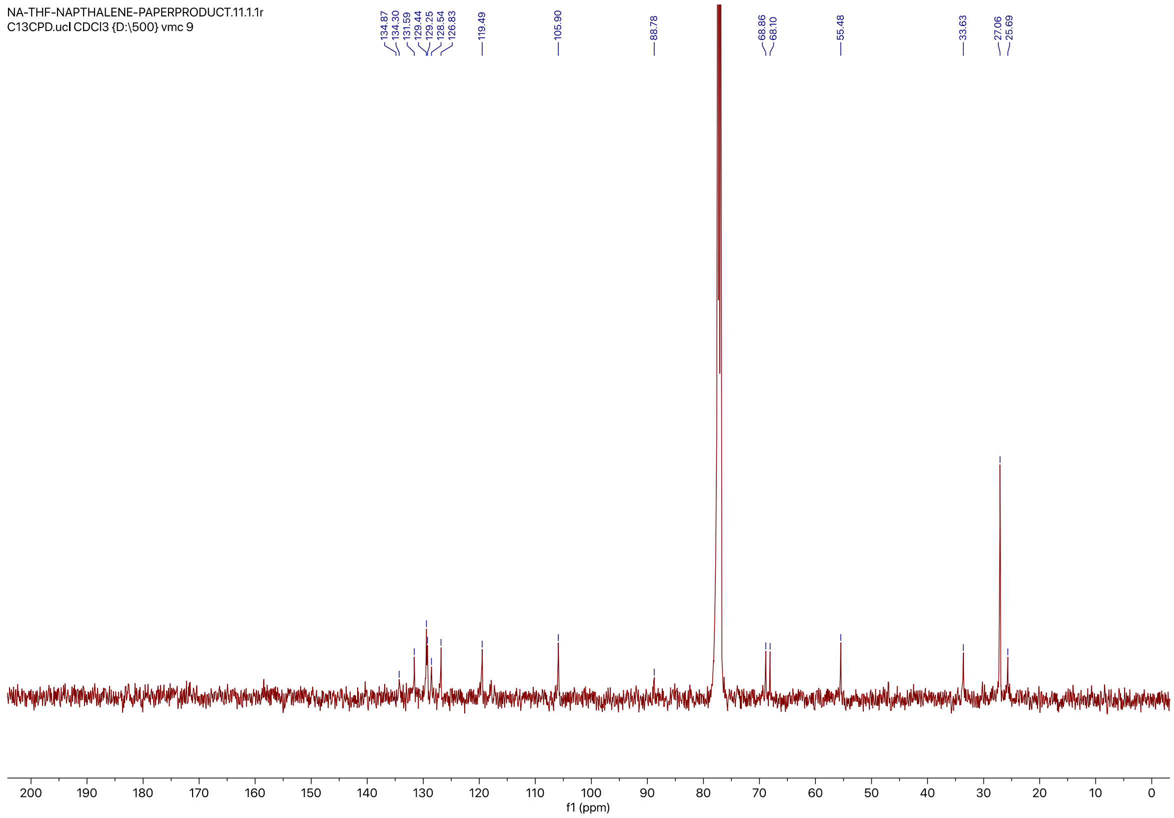
NA-THF-NAPTHALENE-PAPERPRODUCT.11.1.1r
C13CPD.ucl CDCl3 {D:\500} vmc 9
134.87
134.30
131.59
129.44
129.25
128.54
126.83
119.49
105.90
88.78
68.86
68.10
55.48
33.63
27.06
25.69
200 190 180 170 160 150 140 130 120 110 100 90 80 70 60 50 40 30 20 10 0
f1 (ppm)

2-(oct-1-yn-1-yl)tetrahydrofuran – 19a

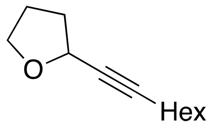

Following application of method B, purification by column chromatography (0-15% EtOAc/cyclohexane) afforded 2-(oct-1-yn-1-yl)tetrahydrofuran **19a** as a yellow oil (31 mg, 0.16 mmol, 63%). ^{1}H NMR (500 MHz, $CDCl_3$) δ 4.60-4.53 (m, 1H), 3.98–3.89 (m, 1H), 3.78 (td, J = 7.8, 6.1 Hz, 1H), 2.19 (td, J = 7.1, 1.8 Hz, 2H), 2.16–2.08 (m, 1H), 2.05–1.97 (m, 1H), 1.96–1.80 (m, 2H), 1.54–1.44 (m, 2H), 1.36-1.25 (m, 6H), 0.88 (t, J = 7.0 Hz, 3H). ^{13}C NMR (126 MHz, $CDCl_3$) δ 85.4 (C), 80.1 (C), 68.6 (CH_2), 67.7 (CH), 33.7 (CH_2), 31.4 (CH_2), 28.7 (CH_2), 28.6 (CH_2), 25.5 (CH_2), 22.6 (CH_2), 18.9 (CH_2), 14.1 (CH_3); IR (thin film): 3022, 2991, 2204. Known compound.[6]

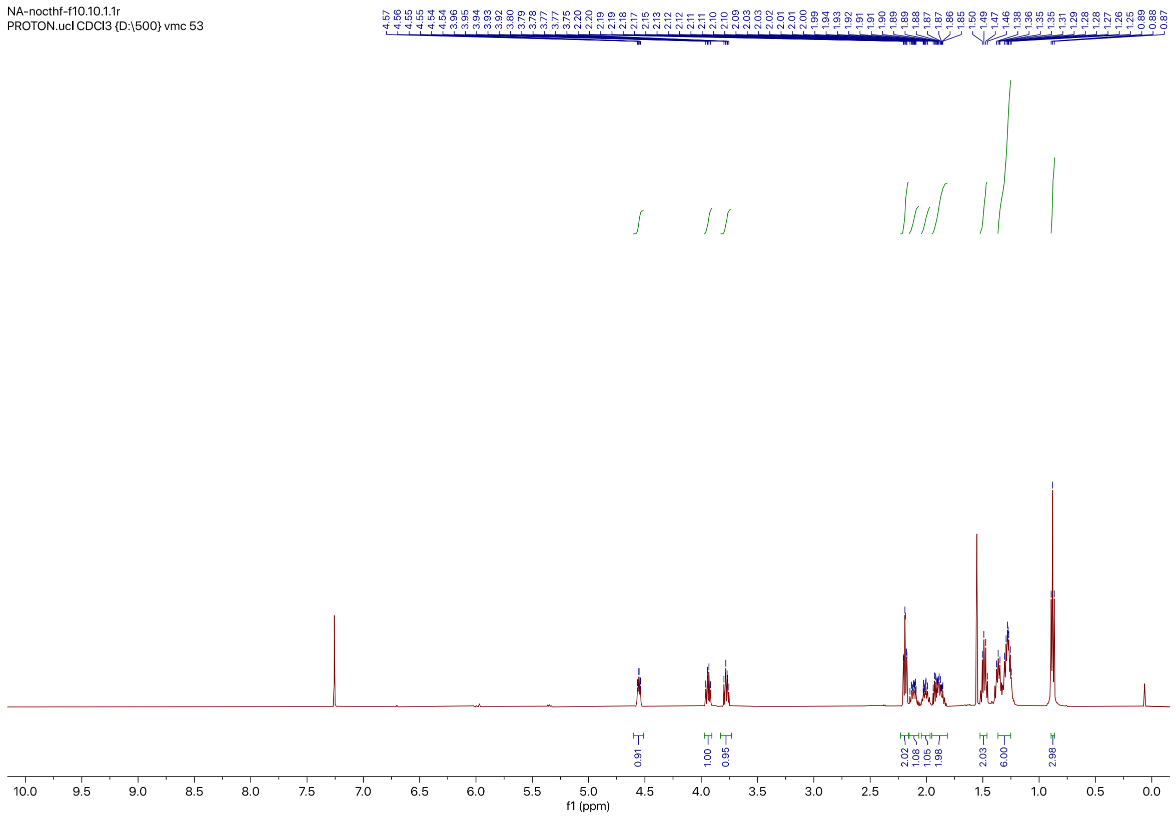

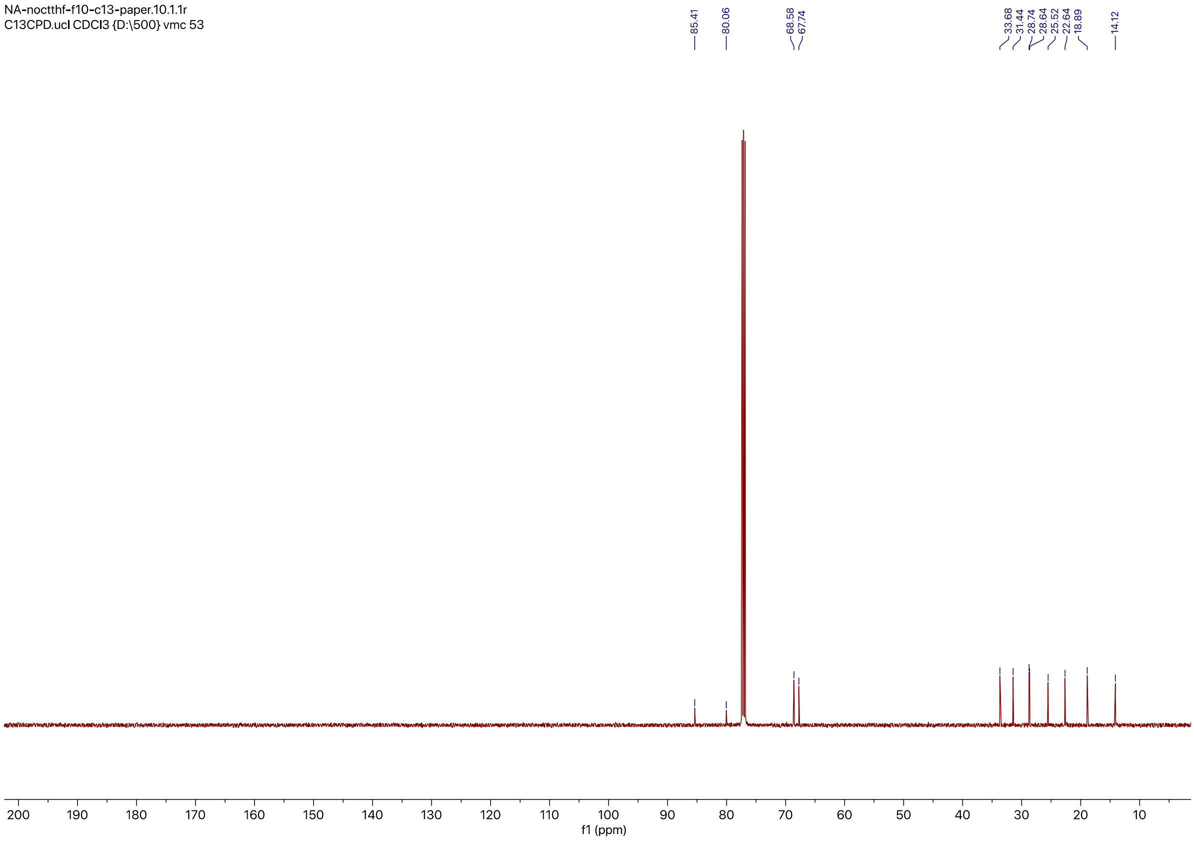

NA-noctthf-f10-c13-paper.10.1.1r
C13CPD.ucl CDCl3 {D:\500} vmc 53
85.41
80.06
68.58
67.74
33.68
31.44
28.74
28.64
25.52
22.64
18.89
14.12
200
190
180
170
160
150
140
130
120
110
100
90
80
70
60
50
40
30
20
10
f1 (ppm)

2-(3,3-Dimethylbut-1-yn-1-yl)tetrahydrofuran – 20a

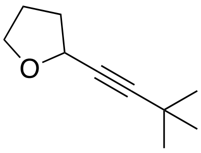

Following application of method B, purification by column chromatography (0-15% EtOAc/cyclohexane) afforded 2-(3,3-dimethylbut-1-yn-1-yl)tetrahydrofuran **20a** as a clear oil (27 mg, 0.18 mmol, 72%). ^{1}H NMR (500 MHz, CDCl3) δ 4.52 (dd, *J* = 7.0, 5.8 Hz, 1H), 3.98–3.89 (m, 1H), 3.81–3.71 (m, 1H), 2.21–2.04 (m, 1H), 2.05–1.92 (m, 2H), 1.93–1.78 (m, 1H), 1.20 (s, 9H). ^{13}C NMR (126 MHz, CDCl3) δ 93.5 (C), 78.3 (C), 68.6 (CH), 67.7 (CH_2), 33.8 (C), 31.1 (CH_3), 27.0 (CH_2), 25.4 (CH_2); IR (thin film): 3002, 2920, 2221 cm^{-1}. Known compound.[6]

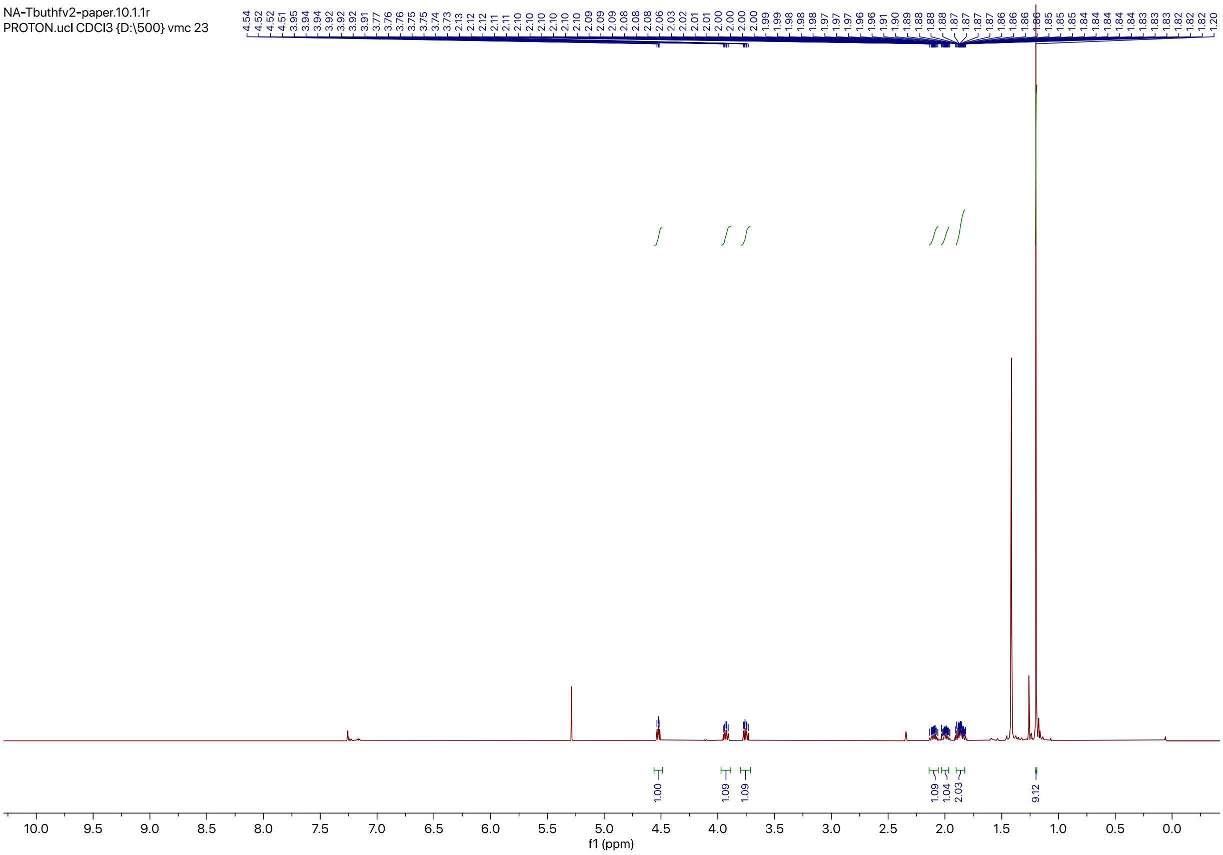

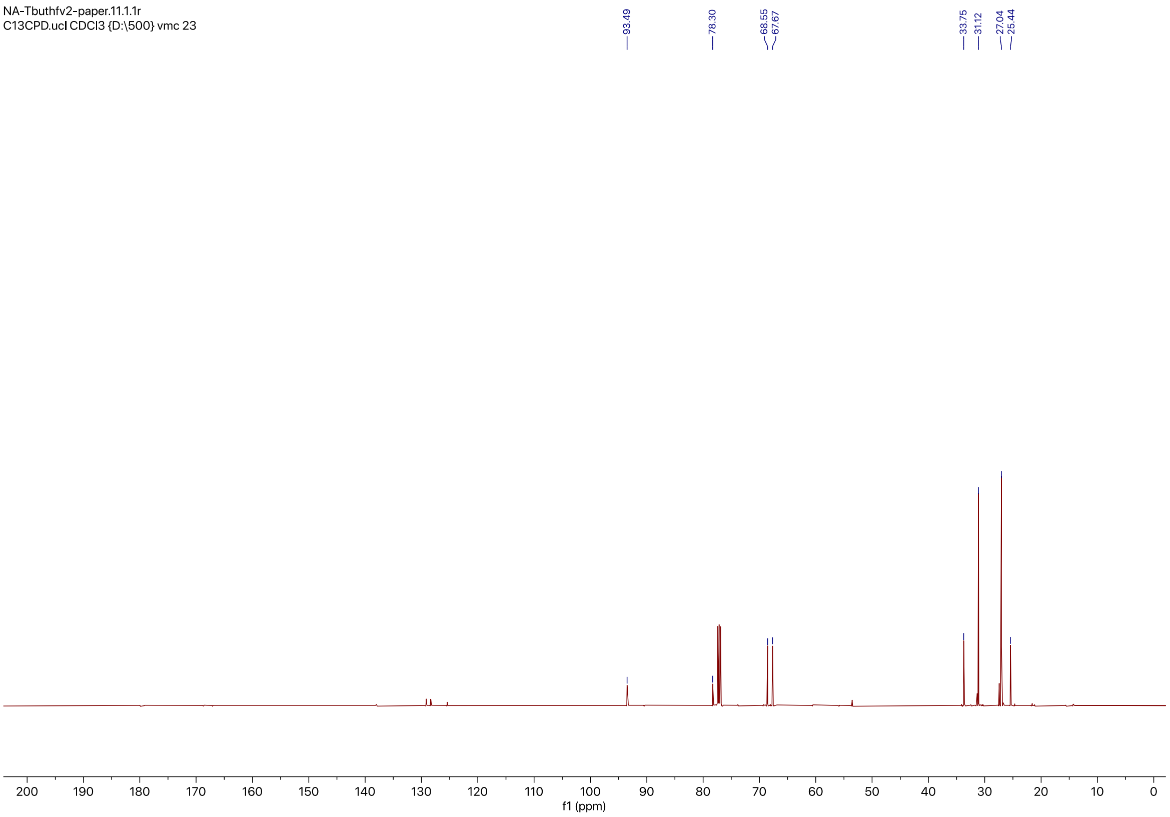
NA-Tbuthfv2-paper.11.1.1r
C13CPD.ucl CDCl3 {D:\500} vmc 23
93.49
78.30
68.55
67.67
33.75
31.12
27.04
25.44
200 190 180 170 160 150 140 130 120 110 100 90 80 70 60 50 40 30 20 10 0
f1 (ppm)

General Procedure for aerobic C-H functionalisation of heterocycles – Method C (vinyl triflone)

To a solution of heterocycle (1.25 mmol, 5.0 eq.) in HFIP (0.40 mmol) and glacial acetic acid (0.40 mmol) was added vinyl triflone (0.25 mmol, 1.0 eq.). The reaction mixture was stirred at 80 °C for 16 h and then poured over sat. aq. $NaHCO_3$ (5 mL). The organics were extracted with EtOAc (3 × 10 mL), dried over $MgSO_4$, filtered and the solvent removed under reduced pressure. The resultant crude residue was purified as described below. Note: the alkenyl triflones were made in accordance with literature procedures.[12,16]

(*Z*)-2-(2-Bromo-2-phenylvinyl)tetrahydrofuran – 21a

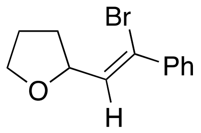

Following application of method B, purification by column chromatography (0-15% EtOAc/cyclohexane) gave **(***Z***)**-2-(2-bromo-2-phenylvinyl)tetrahydrofuran **21a** as a yellow oil (49 mg, 0.20 mmol, 78%). ^{1}H NMR ($CDCl_3$, 700 MHz): δ 7.54–7.50 (m, 2H), 7.37–7.31 (m, 3H), 6.35 (d, *J* = 7.0 Hz, 1H), 4.81 (q, *J* = 7.0 Hz, 1H), 3.96 (dt, *J* = 8.2, 6.9 Hz, 1H), 3.85 (td, *J* = 7.9, 6.2 Hz,1H), 2.32 (dtd, *J* = 12.5, 7.2, 5.4 Hz,1H), 2.03–1.93 (m, 2H), 1.70 (dq, *J* = 12.4, 7.9 Hz, 1H); ^{13}C NMR (176 MHz, $CDCl_3$) δ 139.4 (C), 133.0 (CH), 128.9 (CH), 128.4 (CH), 127.7 (C), 125.6 (C), 79.6 (CH), 68.5 (CH_2), 31.9 (CH_2), 26.2 (CH_2); IR (thin film): 2932, 2902, 1623, 1555, 1532 cm^{-1}. Known compound.[12]

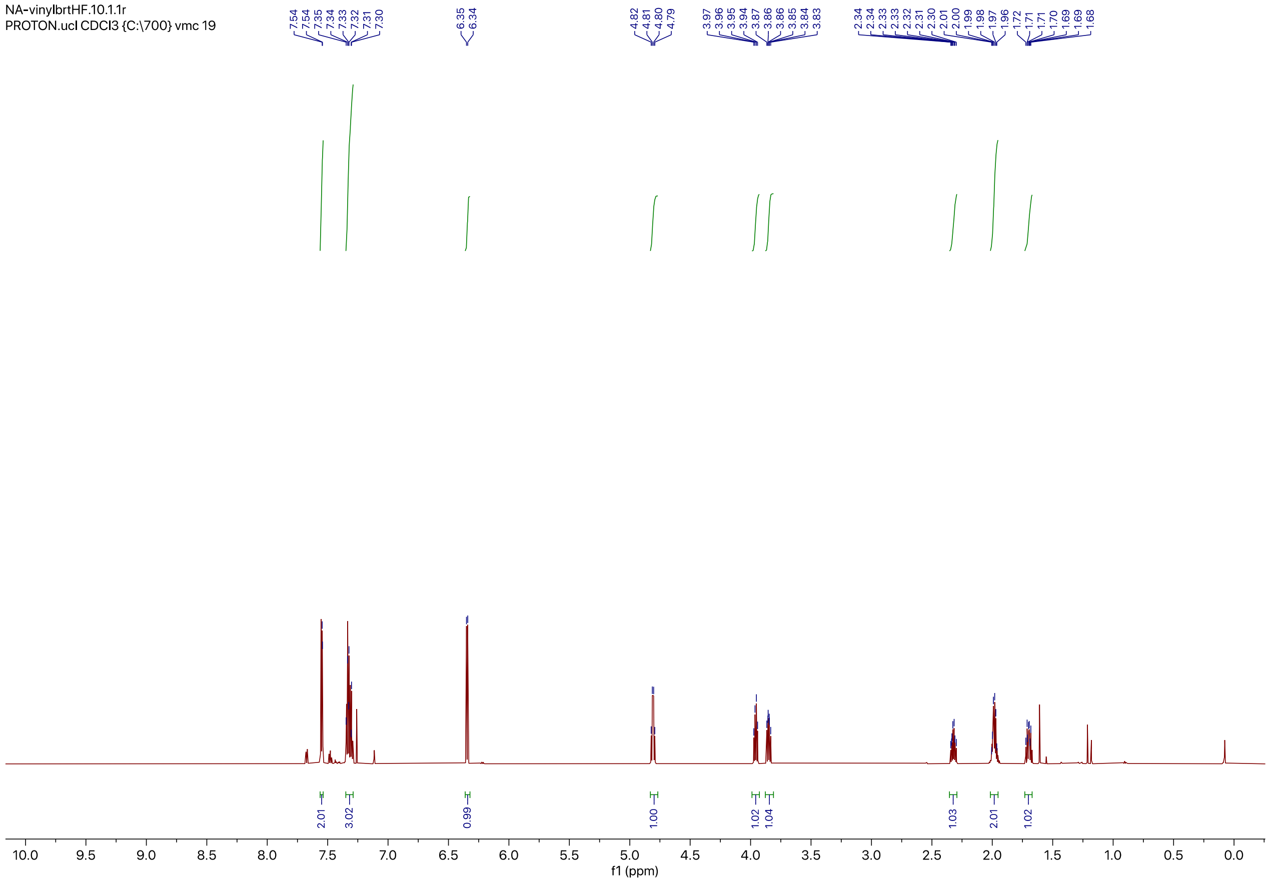

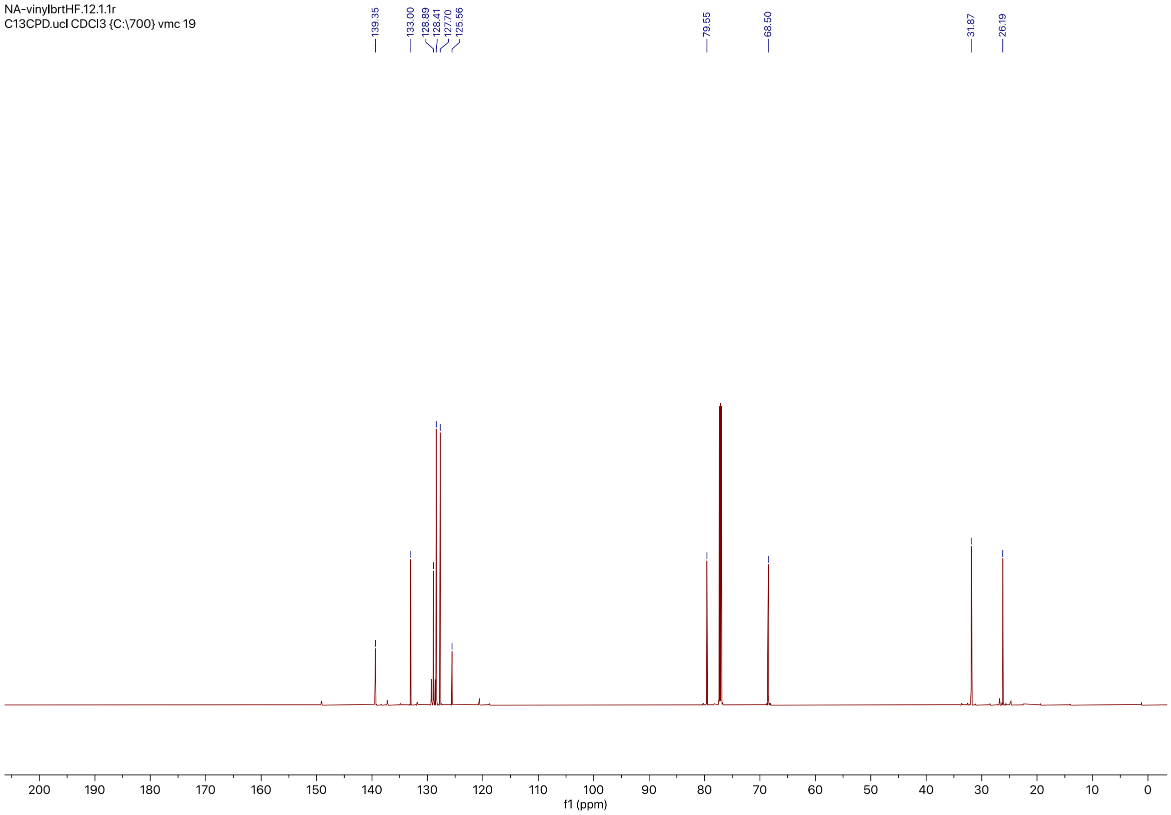
NA-vinylbrtHF.12.1.1r
C13CPD.ucl CDCl3 {C:\700} vmc 19
139.35
133.00
128.89
128.41
127.70
125.56
79.55
68.50
31.87
26.19
200 190 180 170 160 150 140 130 120 110 100 90 80 70 60 50 40 30 20 10 0
f1 (ppm)

(*Z*)-2-(2-Fluoro-2-phenylvinyl)tetrahydrofuran – 22a

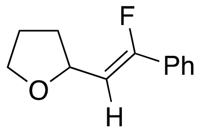

Following application of method B, purification by column chromatography (0-15% EtOAc/cyclohexane) gave (*Z*)-2-(2-fluoro-2-phenylvinyl)tetrahydrofuran **22a** as a colourless oil (31 mg, 0.16 mmol, 67%). ^{1}H NMR ($CDCl_3$, 700 MHz): δ 7.52 (dd, J = 8.4, 1.5 Hz, 2H), 7.39–7.30 (m, 3H), 5.50 (dd, J = 36.9, 8.2 Hz, 1H), 4.94–4.88 (m, 1H), 3.98–3.92 (m, 1H), 3.82 (td, J = 8.1, 6.0 Hz 1H), 2.24-2.19 (m, 1H), 2.05-1.92 (m, 2H), 1.72-1.64 (m, 1H); ^{13}C NMR (176 MHz, $CDCl_3$) δ 157.7 (CF, d, $J_{F,C}$ = 250.7 Hz), 132.1 (C, d, $J_{F,C}$ = 28.2 Hz), 129.2 (CH), 128.6 (CH, d, $J_{F,C}$ = 1.9Hz), 124.5 (CH, d, $J_{F,C}$ = 7.2 Hz), 107.6 (C, d, $J_{F,C}$ = 11.7 Hz), 73.0 (CH), 68.1 (CH_2), 32.8 (CH_2), 26.2 (CH_2); IR (thin film): 3011, 2983, 1625, 1575, 1532 cm^{-1}. Known compound.[12]

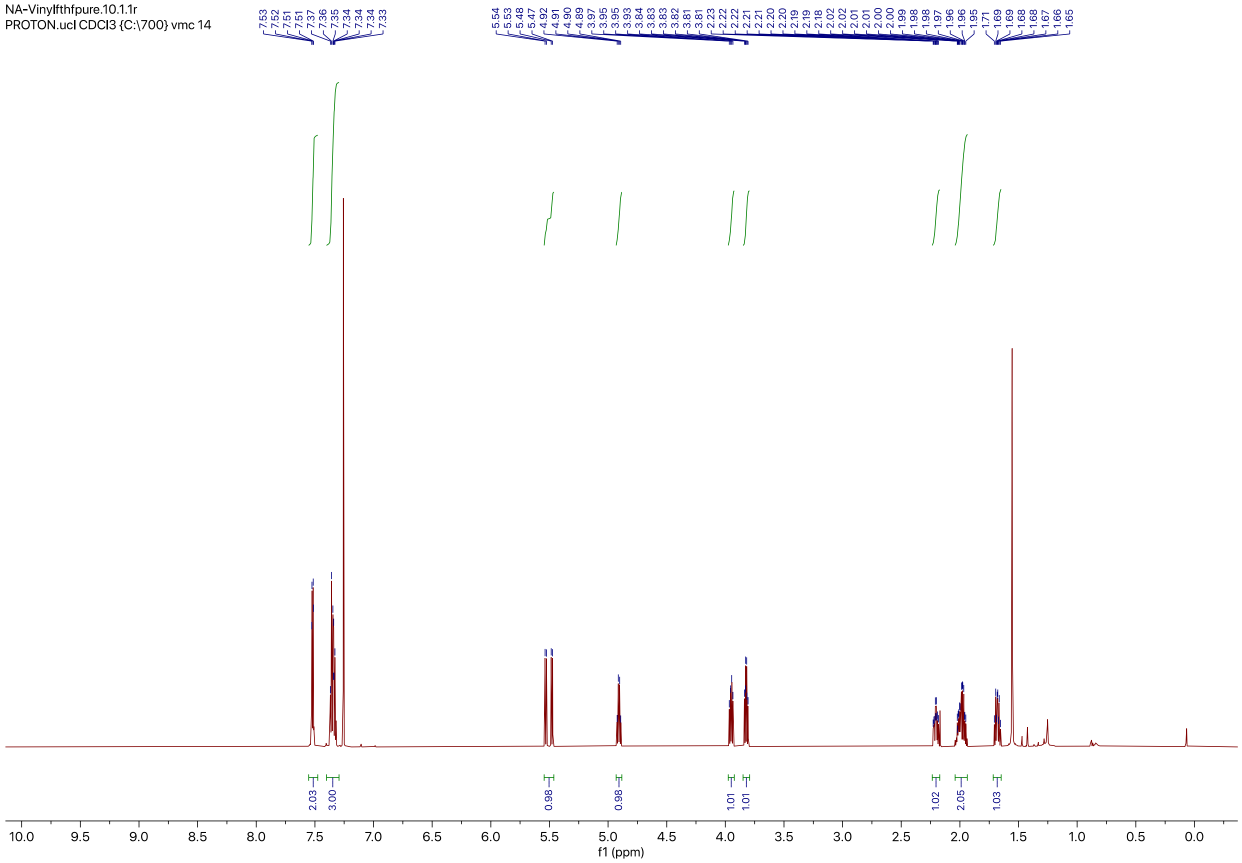

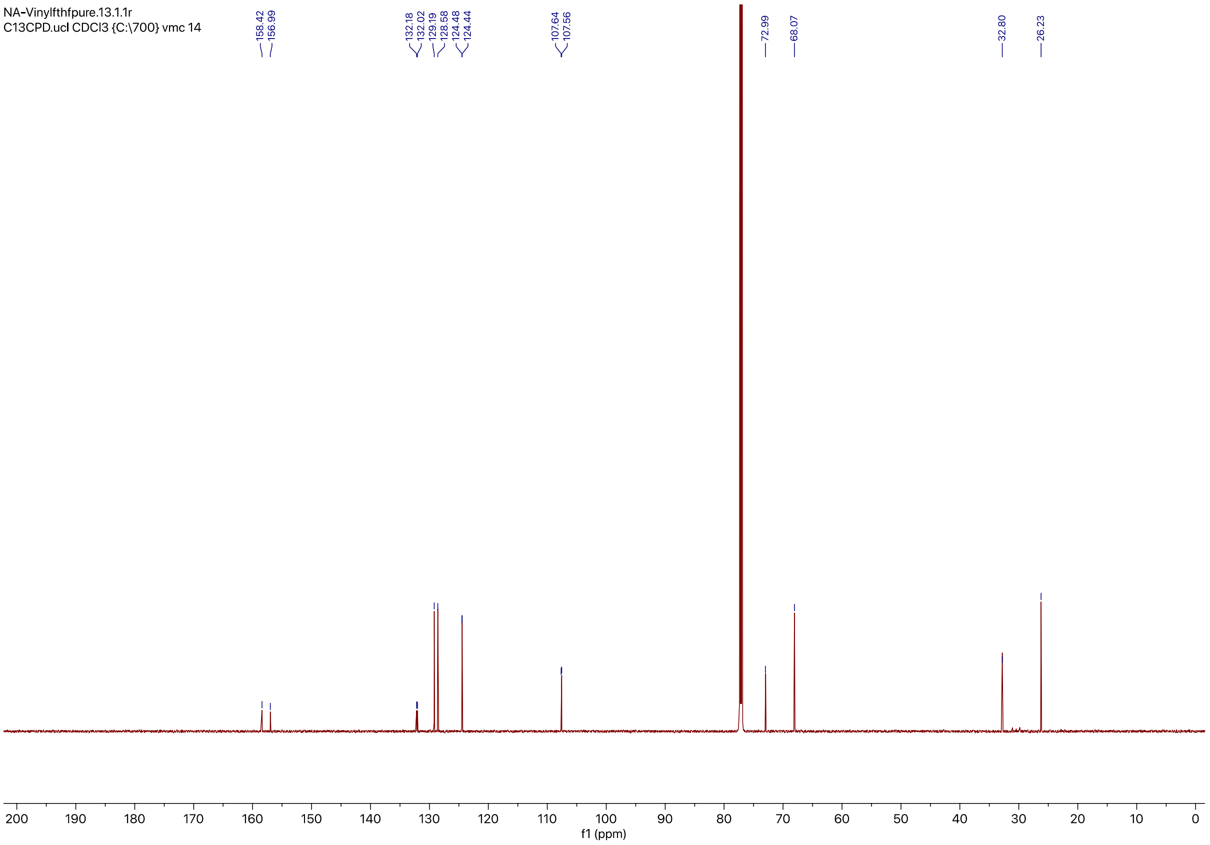
NA-Vinylfthfpure.13.1.1r
C13CPD.ucl CDCl3 {C:\700} vmc 14
158.42
156.99
132.18
132.02
129.19
128.58
124.48
124.44
107.64
107.56
72.99
68.07
32.80
26.23
200 190 180 170 160 150 140 130 120 110 100 90 80 70 60 50 40 30 20 10 0
f1 (ppm)

(*Z*)-2-(2-iodo-2-phenylvinyl)tetrahydrofuran – 23a

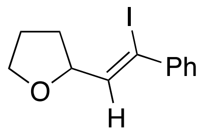

Following application of method B, purification by column chromatography (0-15% EtOAc/cyclohexane) gave (*Z*)-2-(2-iodo-2-phenylvinyl)tetrahydrofuran **23a** as a yellow oil (54 mg, 0.18 mmol, 72%). ^{1}H NMR ($CDCl_3$, 500 MHz): δ ^{1}H NMR (500 MHz, $CDCl_3$) δ 7.73–7.48 (m, 2H), 7.45–7.19 (m, 3H), 6.35 (d, *J* = 7.0 Hz, 1H), 4.81 (m, 1H), 3.96 (m, 1H), 3.85 (m, 1H) 2.38–2.18 (m, 1H), 2.07–1.84 (m, 2H), 1.83–1.53 (m, 1H). ^{13}C NMR (126 MHz, $CDCl_3$) δ 139.4 (CH), 133.0 (C), 128.9 (CH), 128.3 (CH), 127.7 (CH), 125.5 (CH), 79.6 (C), 68.5 (CH_2), 31.9 (CH_2), 26.2 (CH). 3023, 2981, 2904, 1635, 1550, 1532 cm^{-1}. Known Compound.[12]

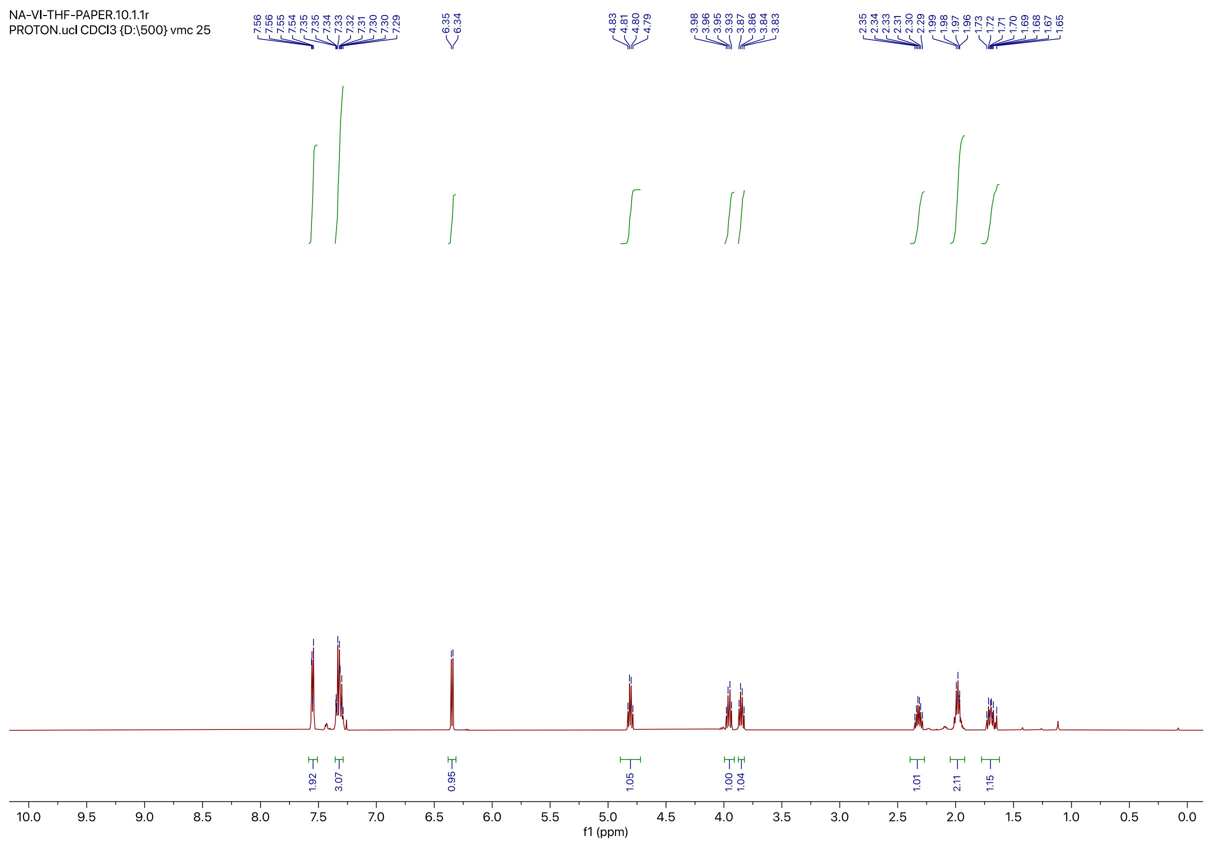

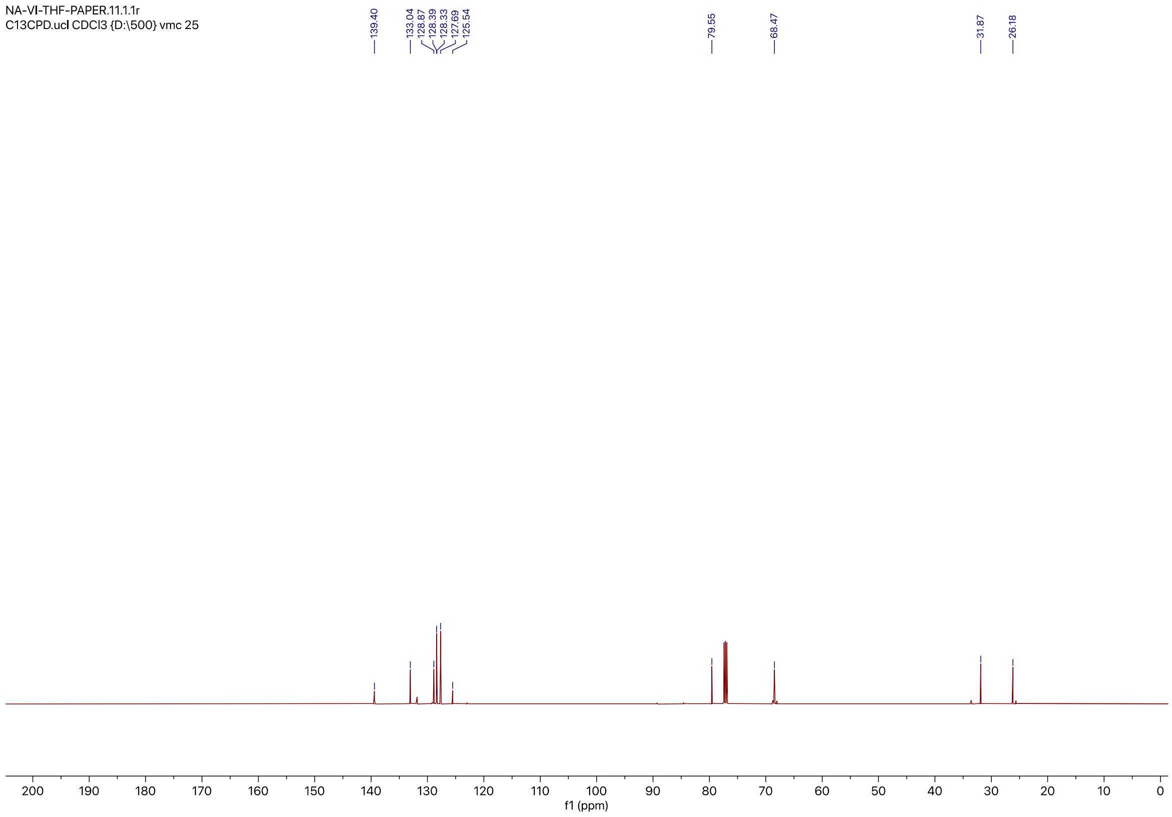
NA-VI-THF-PAPER.11.1.1r
C13CPD.ucl CDCl3 {D:\500} vmc 25
139.40
133.04
128.87
128.39
128.33
127.69
125.54
79.55
68.47
31.87
26.18
f1 (ppm)

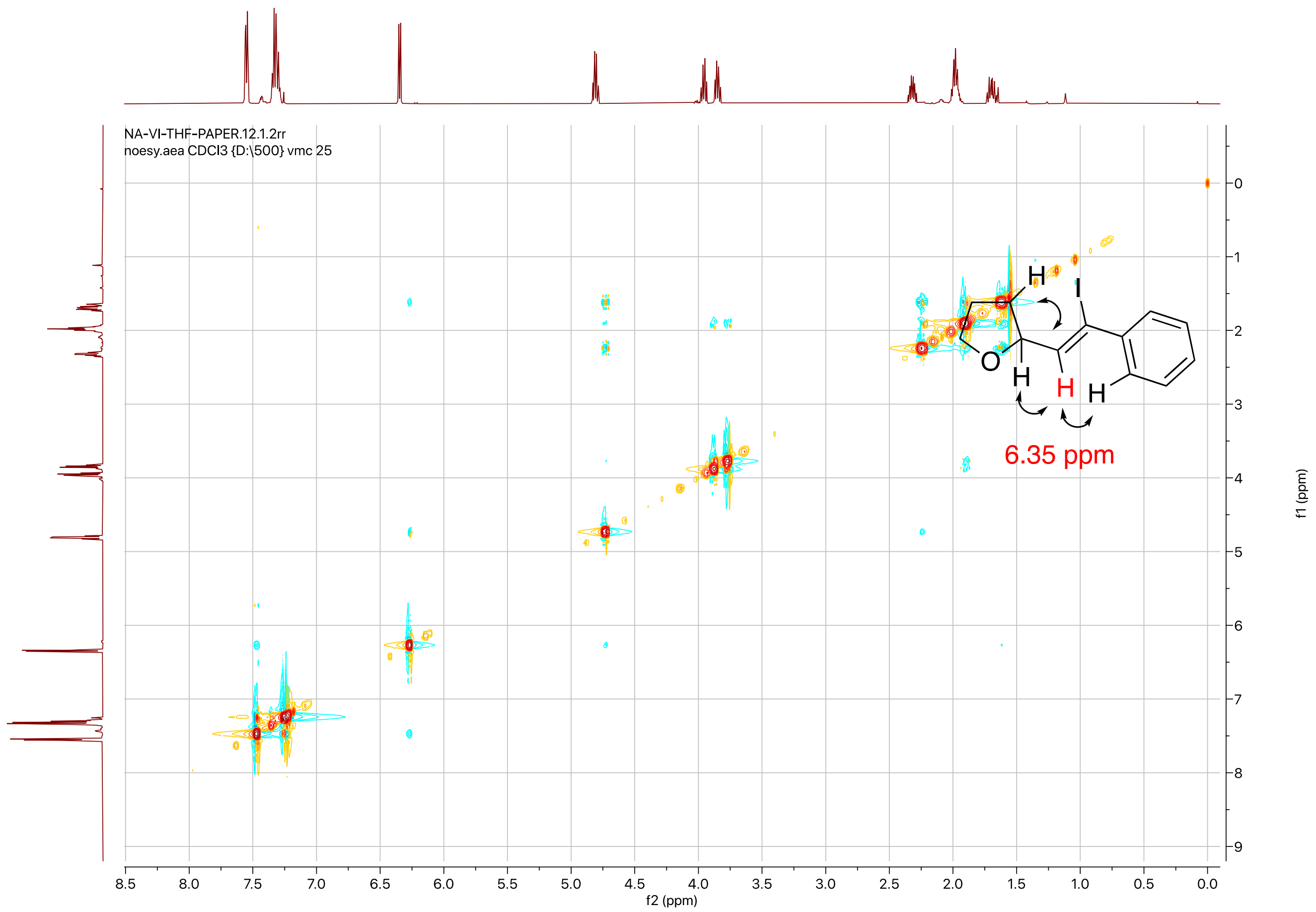
NA-VI-THF-PAPER.12.1.2rr
noesy.aea CDCl3 {D:\500} vmc 25
6.35 ppm
f2 (ppm)
f1 (ppm)

(*E*)-2-styryltetrahydrofuran – 24a

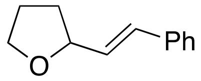

Following application of method B, purification by column chromatography (0-15% EtOAc/cyclohexane) gave (*E*)-2-styryltetrahydrofuran **24a** as a yellow oil (31 mg, 0.18 mmol, 71%). ^{1}H NMR (600 MHz, $CDCl_3$) δ 7.38 (d, *J* = 7.6 Hz, 2H), 7.30 (t, *J* = 7.6 Hz, 2H), 7.22 (t, *J* = 7.2 Hz, 1H), 6.58 (d, *J* = 15.8 Hz, 1H), 6.20 (dd, *J* = 15.8, 6.6 Hz, 1H), 4.47 (q, *J* = 6.9 Hz, 1H), 3.99–3.93 (m, 1H), 3.88–3.78 (m, 1H), 2.13 (td, *J* = 12.4, 7.2 Hz, 1H), 2.01–1.92 (m, 2H), 1.71 (dq, *J* = 12.4, 7.7 Hz, 1H). ^{13}C NMR (151 MHz, $CDCl_3$) δ 137.0 (C), 130.7 (CH), 130.6 (CH), 128.7 (CH), 127.6 (CH), 126.6 (CH), 79.8 (CH), 68.3 (CH_2), 32.5 (CH_2), 26.1 (CH_2). IR (thin film): 2926, 2900, 1629, 1554, 1515 cm^{-1}. Known compound.[16]

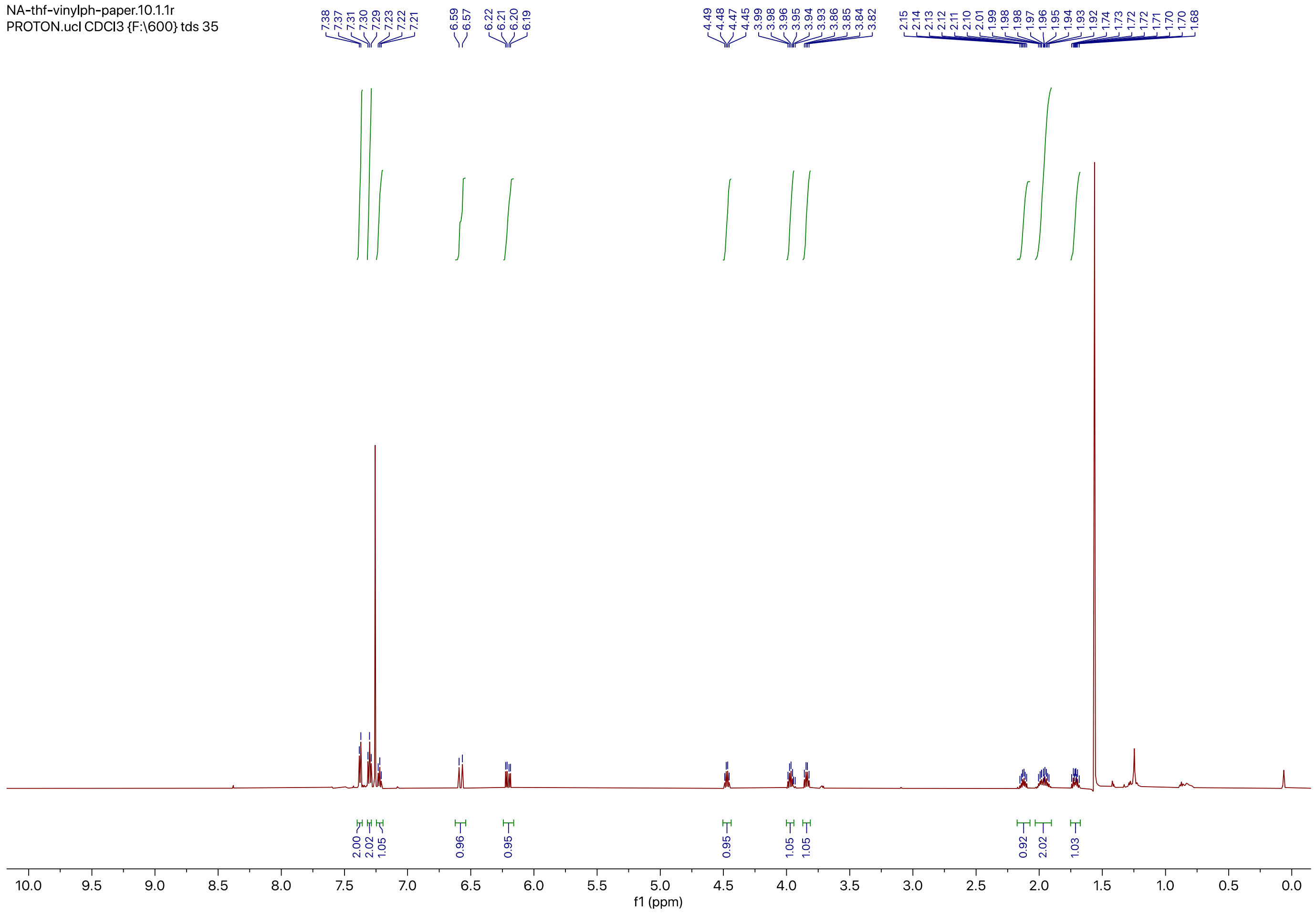

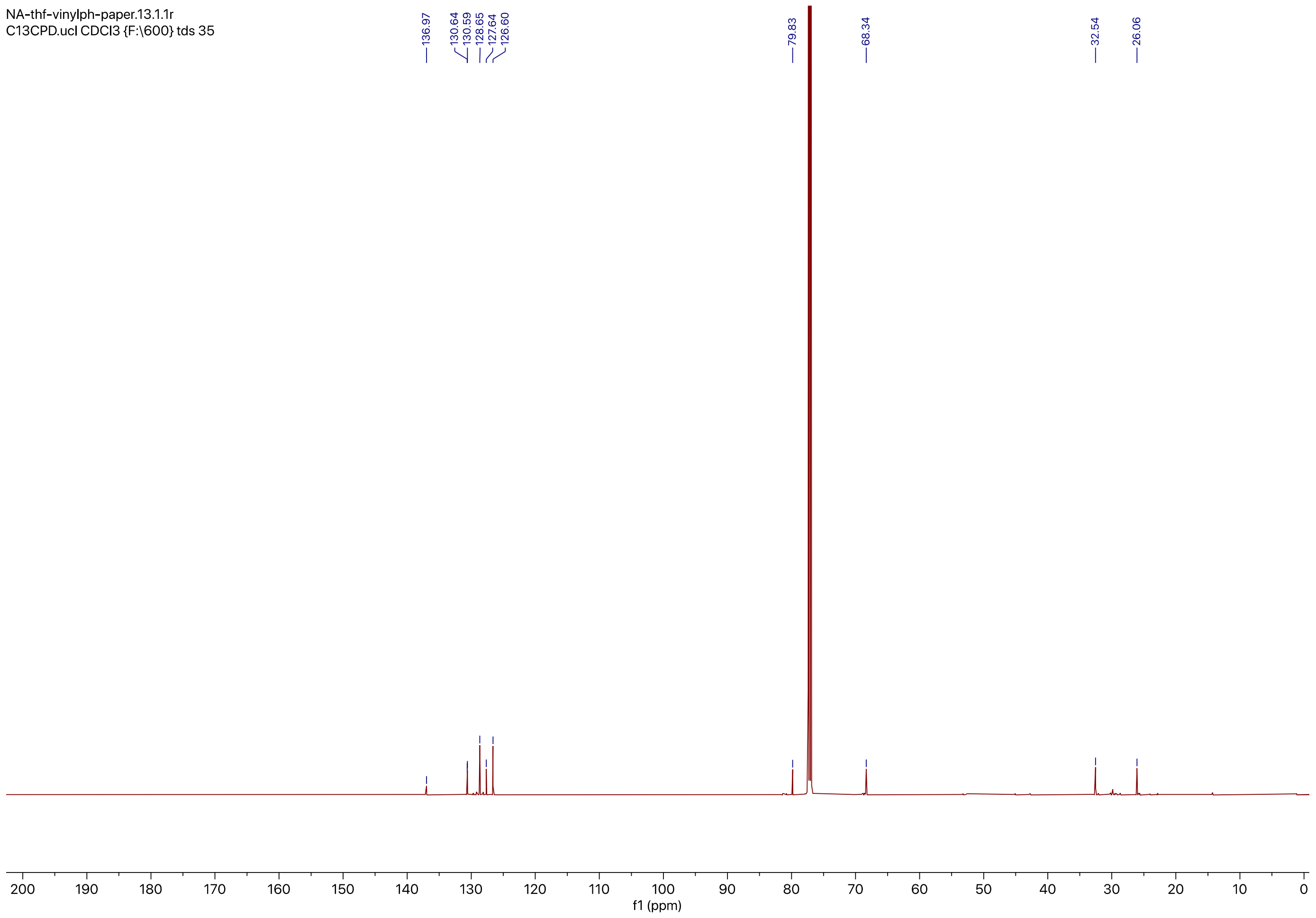
NA-thf-vinylph-paper.13.1.1r
C13CPD.ucl CDCl3 {F:\600} tds 35
136.97
130.64
130.59
128.65
127.64
126.60
79.83
68.34
32.54
26.06
200
190
180
170
160
150
140
130
120
110
100
90
80
70
60
50
40
30
20
10
0
f1 (ppm)

(*E*)-2-(4-pentylstyryl)tetrahydrofuran– 25a

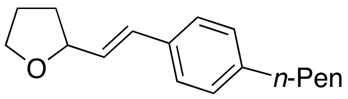

Following application of method B, purification by column chromatography (0-15% EtOAc/cyclohexane) gave (*E*)-2-(4-pentylstyryl)tetrahydrofuran **25a** as a yellow oil (35 mg, 0.15 mmol, 58%). δ ^{1}H NMR (500 MHz, $CDCl_3$): δ 7.29 (d, *J* = 8.1 Hz, 2H), 7.11 (d, *J* = 8.0 Hz, 2H), 6.55 (d, *J* = 15.8 Hz, 1H), 6.15 (dd, *J* = 15.8, 6.7 Hz, 1H), 4.46 (q, *J* = 6.9 Hz, 1H), 4.00–3.92 (m, 1H), 3.90–3.79 (m, 1H), 2.61–2.52 (m, 2H), 2.11 (dt, *J* = 12.0, 5.6 Hz, 1H), 1.99–1.87 (m, 2H), 1.70 (dq, *J* = 12.0, 7.6 Hz, 1H), 1.66–1.55 (m, 3H), 1.34–1.30 (m, 5H), 0.95–0.83 (m, 3H). ^{13}C NMR (126 MHz, $CDCl_3$) δ 142.6 (C), 134.4 (CH), 130.6 (CH), 129.7 (CH), 128.7 (CH), 126.5 (CH), 79.9 (CH), 68.3 (CH_2), 35.8 (CH_2), 32.6 (CH_2), 31.6 (CH_2), 31.2 (CH_2), 26.1 (CH_2), 22.7 (CH_2), 14.2 (CH_3). IR (thin film): 2926, 2900, 1629, 1554, 1515 cm^{-1}. IR (thin film): 2991, 2926, 2900, 1634, 1540, 1511 cm^{-1}. HRMS (ESI) calcd for $C_{17}H_{24}O$ $[M+H]^+$ 245.1822; observed 245.1813.

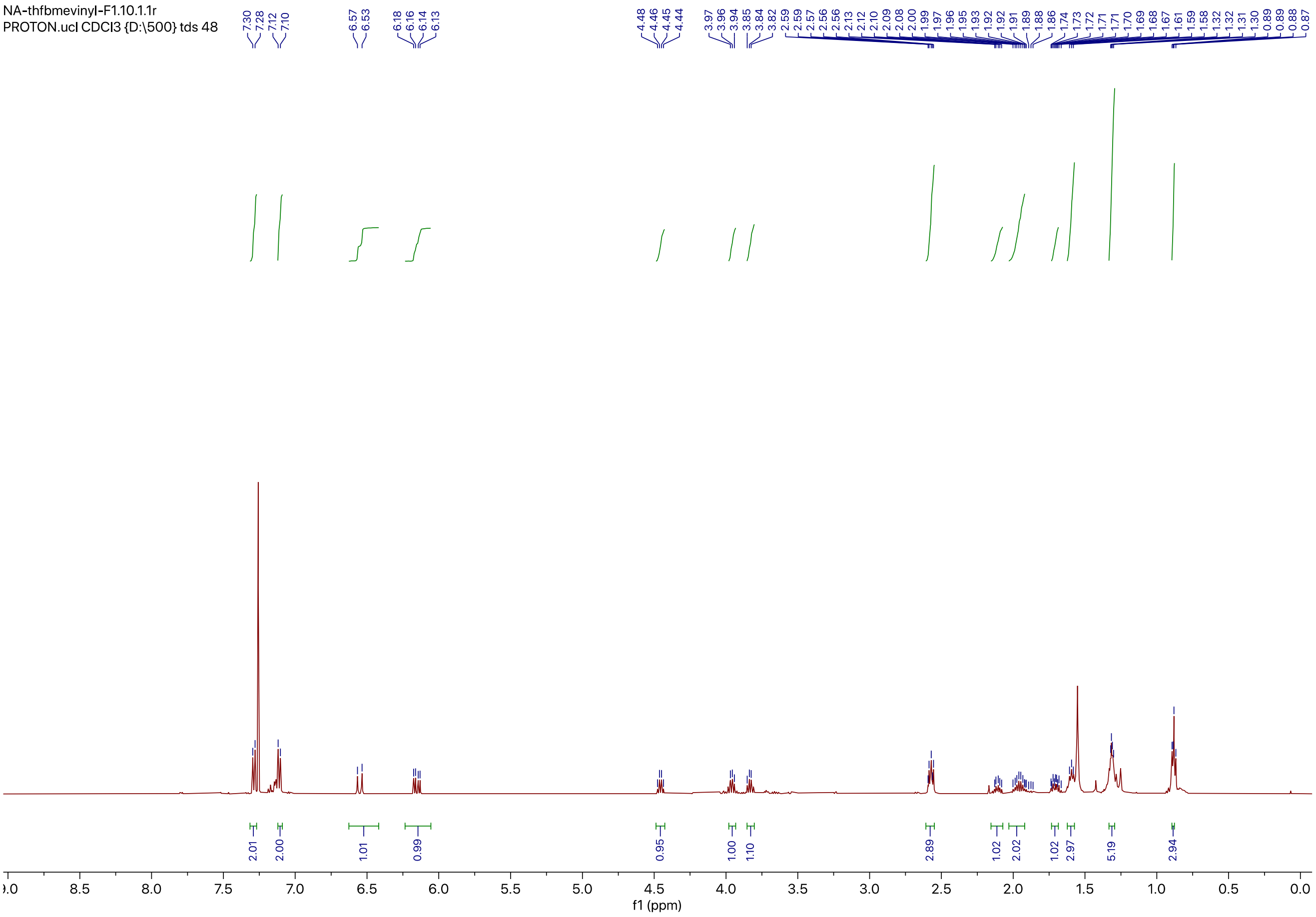

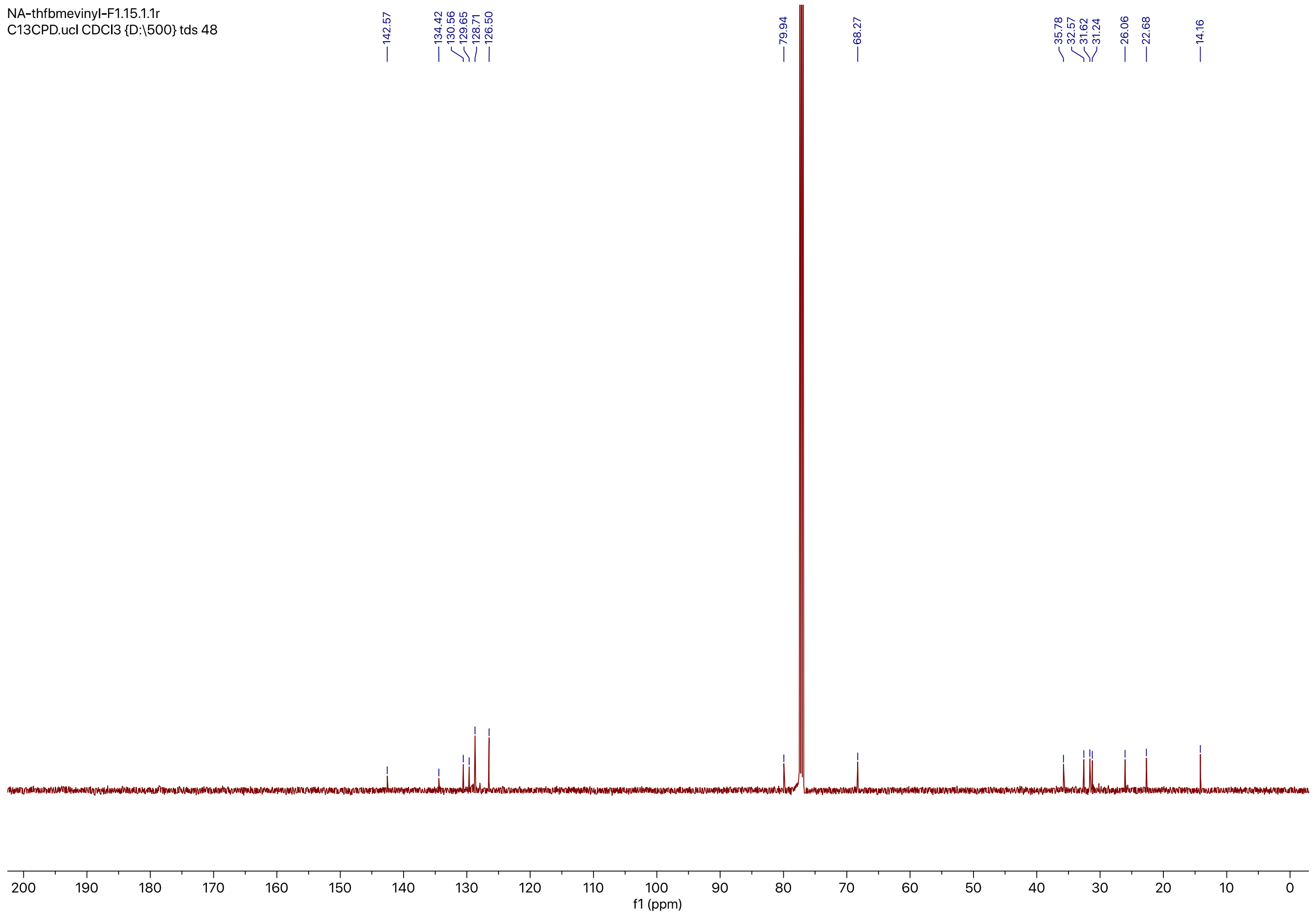

NA-thfbmevinyl-F1.15.1.1r
C13CPD.ucl CDCl3 {D:\500} tds 48
142.57
134.42
130.56
129.65
128.71
126.50
79.94
68.27
35.78
32.57
31.62
31.24
26.06
22.68
14.16
200 190 180 170 160 150 140 130 120 110 100 90 80 70 60 50 40 30 20 10 0
f1 (ppm)

2-phenethyltetrahydrofuran – 26

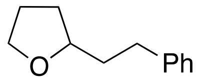

2-(phenylethynyl)tetrahydrofuran **8a** (0.50 mmol, 121mg) in THF (5 mL) was added to a flask containing 10% Pd on C (20 mg) under a N_2 atmosphere. The N_2 was evacuated and H_2 was purged through the flask and bubbled through the solution. The resultant solution was stirred for 16 h under a H_2 atmosphere at room temperature. The resultant solution was filtered through celite and the filter cake was washed with ethyl acetate. The resulting solution was concentrated and gave 2-phenethyltetrahydrofuran **26** as a yellow liquid (43 mg, 0.49 mmol, 97%). ^{1}H NMR (500 MHz, $CDCl_3$) δ 7.32–7.25 (m, 2H), 7.24–7.15 (m, 3H), 3.94–3.86 (m, 1H), 3.82 (qn, *J* = 7.4 Hz, 1H), 3.73 (td, *J* = 7.9, 6.4 Hz, 1H), 2.81–2.73 (m, 2H), 2.66 (ddd, *J* = 13.8, 10.0, 6.3 Hz, 1H), 2.02-1.95 (m, 1H), 1.93–1.83 (m, 3H), 1.82-1.73 (m, 1H), 1.48 (dq, *J* = 11.8, 7.7 Hz, 1H). ^{13}C NMR (126 MHz, $CDCl_3$) δ 142.4 (C), 128.5 (CH), 128.4 (CH), 125.8 (CH), 78.8 (CH), 67.8 (CH_2), 37.6 (CH_2), 32.8 (CH_2), 31.5 (CH_2), 25.9 (CH_2). Known compound.[13]

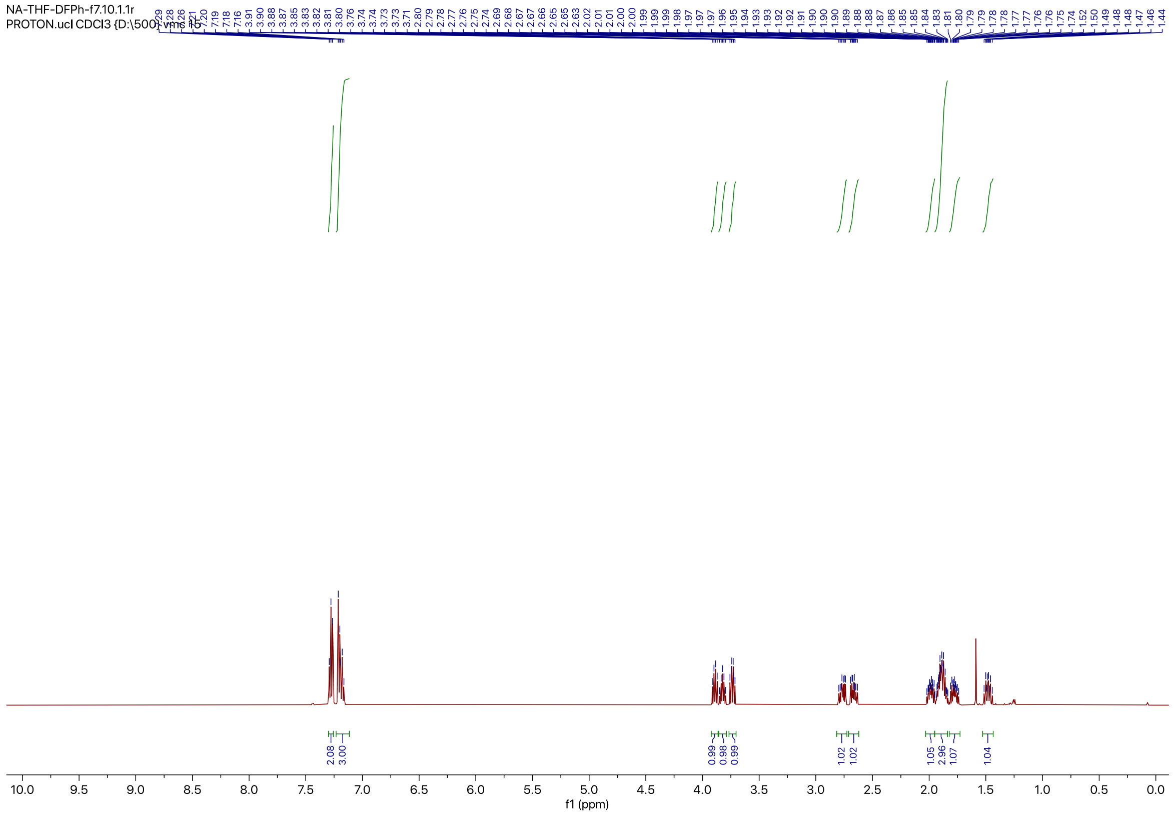

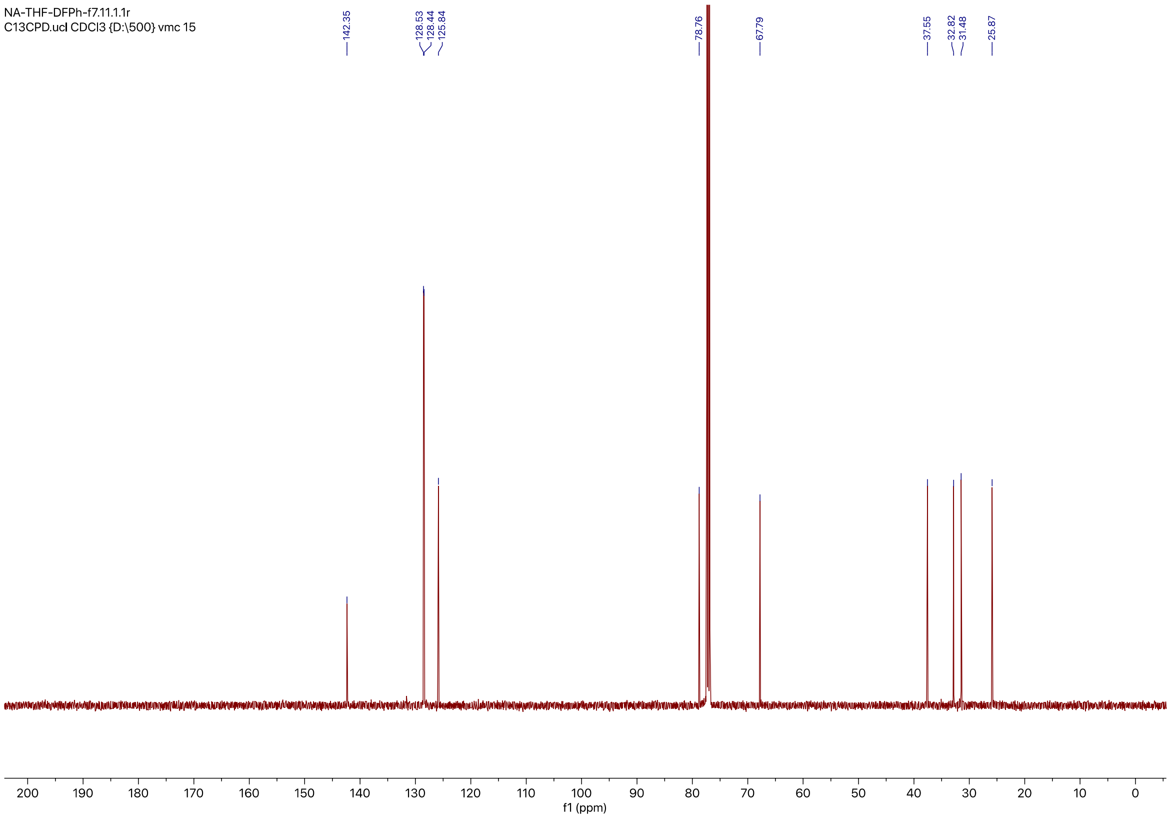
NA-THF-DFPh-f7.11.1.1r
C13CPD.ucl CDCl3 {D:\500} vmc 15
142.35
128.53
128.44
125.84
78.76
67.79
37.55
32.82
31.48
25.87
200
190
180
170
160
150
140
130
120
110
100
90
80
70
60
50
40
30
20
10
0
f1 (ppm)

2,2-dichloro-1-phenyl-2-(tetrahydrofuran-2-yl)ethan-1-one – 27

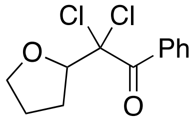

Trichloroisocyanuric acid (116 mg, 0.5 mmol) was added to a stirring solution of 2-(phenylethynyl)tetrahydrofuran **8a** (0.5 mmol) in MeCN/H2O (2 mL, 10:1). Once the reaction was complete (TLC), the solvent was removed under reduced pressure before the residue was purified by column chromatography (0-10% EtOAc/cyclohexane) to give the dihalohydroxyketone **27** as a clear liquid (116 mg, 92%). ^{1}H NMR (500 MHz, $CDCl_3$) δ 8.26 – 8.20 (m, 2H), 7.58 (t, *J* = 7.4 Hz, 1H), 7.46 (t, *J* = 7.8 Hz, 2H), 4.88 (t, *J* = 7.2 Hz, 1H), 4.06 – 3.93 (m, 2H), 2.33 (dtd, *J* = 12.1, 7.9, 4.3 Hz, 1H), 2.19 (dq, *J* = 13.0, 8.3 Hz, 1H), 2.13 – 2.03 (m, 1H), 1.97 (dqn, *J* = 12.1, 8.3 Hz, 1H). ^{13}C NMR (126 MHz, $CDCl_3$) δ 188.6 (C=O), 133.4 (C), 132.8 (CH), 130.8 (CH), 128.2 (CH), 89.0 (C), 83.4 (CH), 70.6 (CH_2), 28.9 (CH_2), 26.3 (CH_2); IR (thin film): 2965, 1677, 1600, 1250, 1199 cm^{-1}; HRMS (ESI) calcd for $C_{12}H_{12}Cl_2O_2$ $[M^{35}Cl+H]^+$ 259.0287; observed 259.0282.

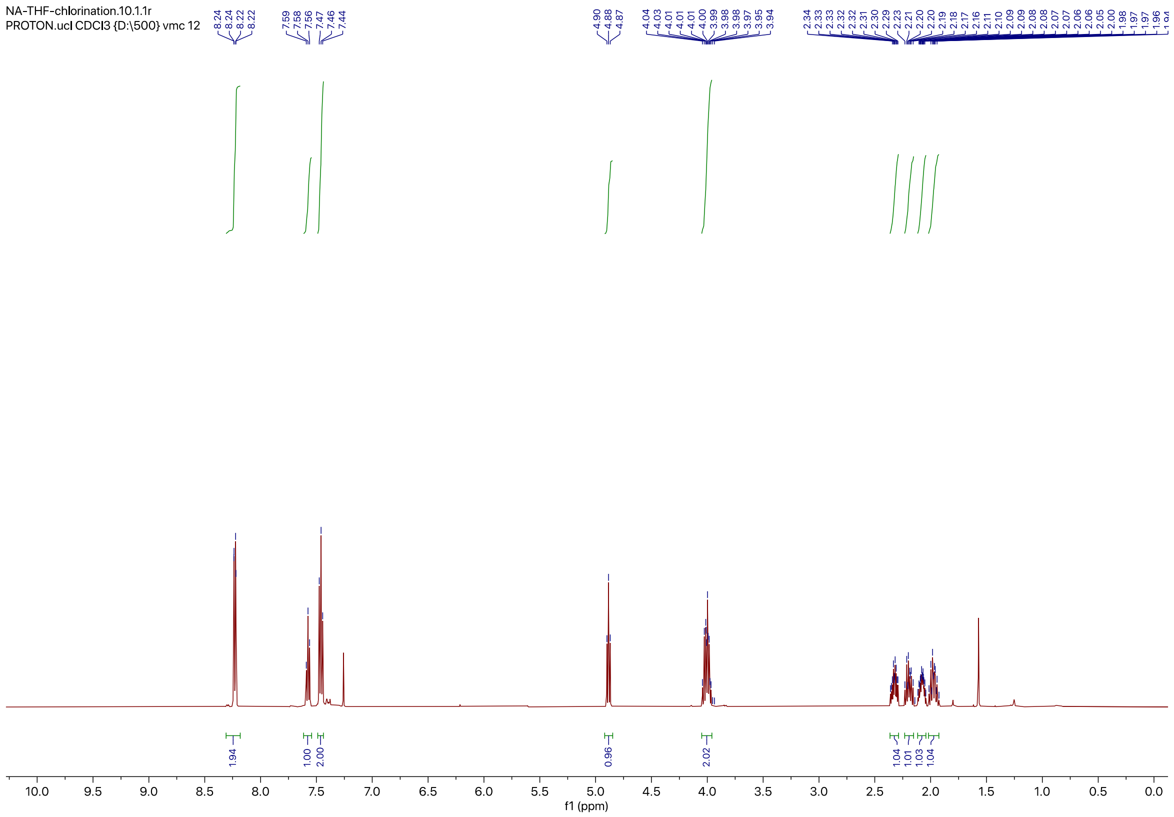

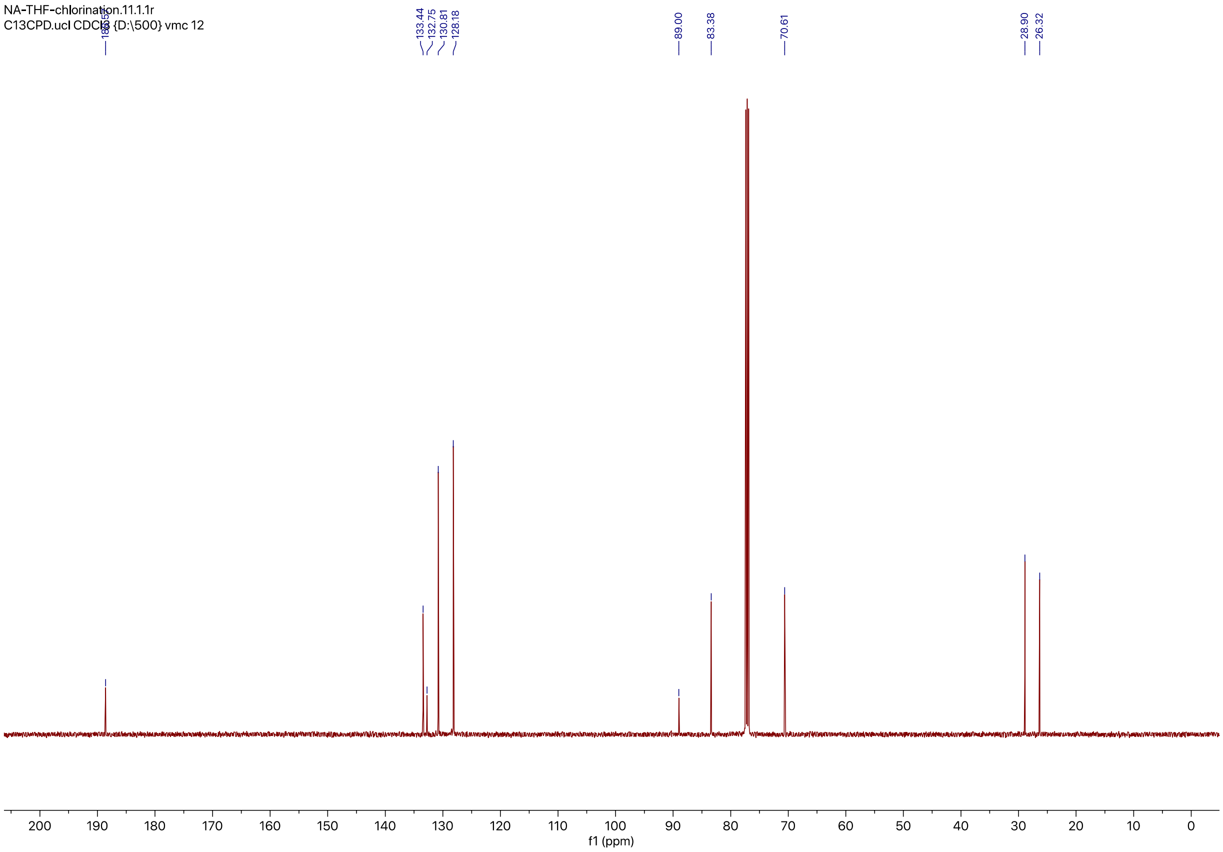

NA-THF-chlorination.11.1.1r
C13CPD.ucl CDCl3 {D:\500} vmc 12
188.57
133.44
132.75
130.81
128.18
89.00
83.38
70.61
28.90
26.32
200
190
180
170
160
150
140
130
120
110
100
90
80
70
60
50
40
30
20
10
0
f1 (ppm)

(*Z*)-2-(2-(3,5-bis(trifluoromethyl)phenyl)-2-phenylvinyl)tetrahydrofuran – 28

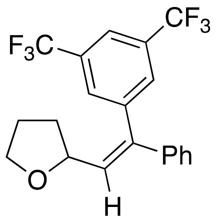

In a sealed tube, (*Z*)-2-(2-iodo-2-phenylvinyl)tetrahydrofuran **9ac** (75 mg, 0.25 mmol), $Pd(PPh_3)_4$ (10 mg), K_2CO_3 (100 mg, 0.75 mmol), 3,5-bis(trifluoromethyl)phenylboronic acid (71 mg, 0.275 mmol) were mixed together in dioxane:water (10:1, 1.5 mL) and heated at 70 °C for 16 h under an argon atmosphere. The resultant mixture was cooled to rt and water (10 mL) was added, the resulting mixture was extracted with diethyl ether (3 x 30 mL). The combined organics were dried over $MgSO_4$, filtered and the solvent removed under reduced pressure. The resultant crude residue was purified by column chromatography (0-10% EtOAc/cyclohexane) to afford (*Z*)-2-(2-(3,5-bis(trifluoromethyl)phenyl)-2-phenylvinyl)tetrahydrofuran **28** as a clear oil (89 mg, 0.23 mmol). ^{1}H NMR (500 MHz, $CDCl_3$) δ 7.86 (s, 1H), 7.69 (s, 2H), 7.31 (m, 3H), 7.22–7.15 (m, 2H), 6.19 (d, *J* = 9.0 Hz, 1H), 4.15–4.07 (m, 1H), 4.01–3.91 (m, 1H), 3.75 (td, *J* = 8.0, 5.9 Hz, 1H), 2.09-1.99 (m, 2H), 1.94-1.85 (m, 1H), 1.80-1.71 (m, 1H). ^{13}C NMR (151 MHz, $CDCl_3$) δ 141.7 (C), 141.3 (C), 140.6 (C), 132.4 (CH), 131.6 (C, q, $J_{F,C}$ = 33.3 Hz), 130.2 (br s, CH), 128.7 (CH), 128.4 (C), 127.7 (C), 123.5 (CF_3, q, $J_{F,C}$ = 272.7 Hz), 121.7–121.5 (m, CH), 76.3 (CH), 68.4 (CH_2), 33.2 (CH_2), 26.5 (CH_2); HRMS (ESI) calcd for $C_{20}H_{16}F_6O$ $[M+H]^+$ 387.1178; observed 387.117.

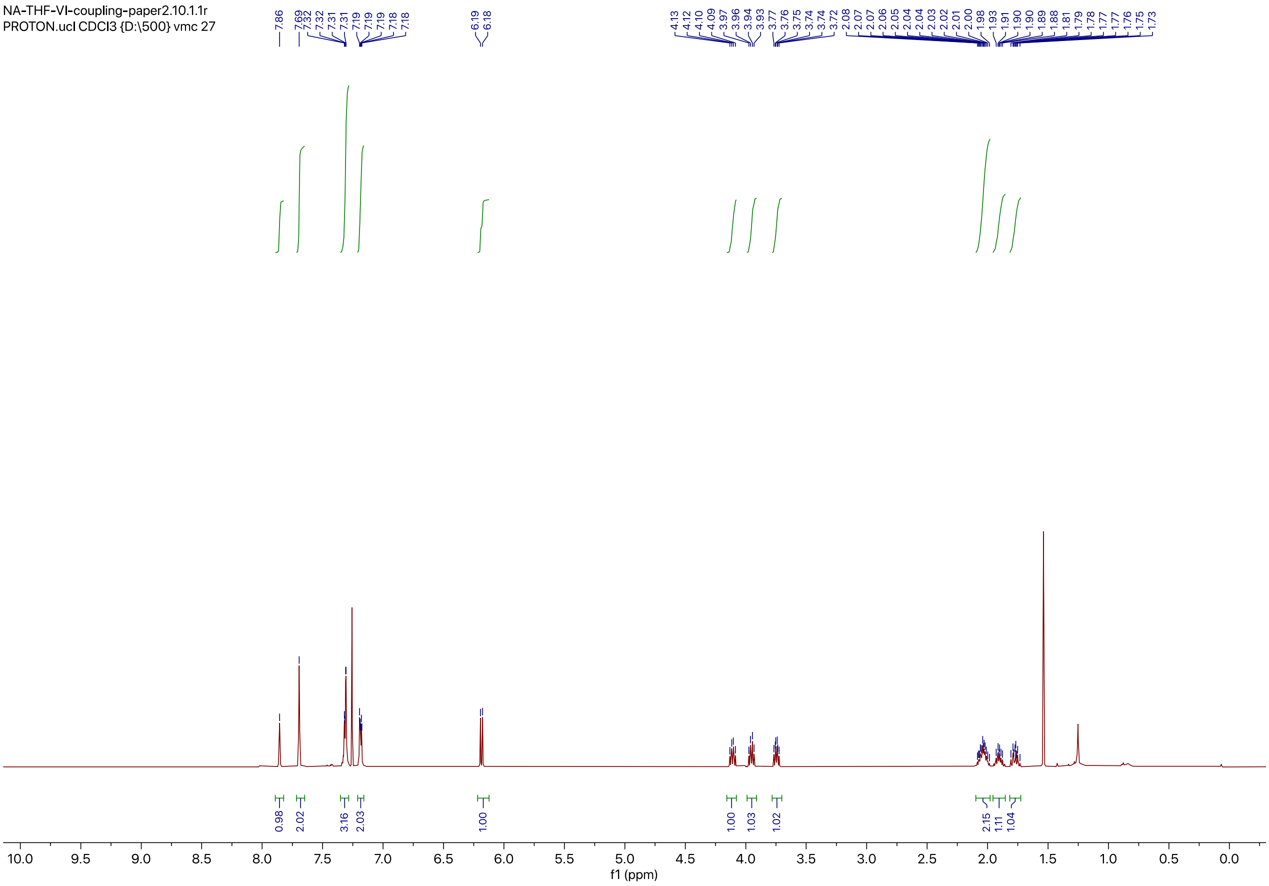
NA-THF-VI-coupling-paper2.10.1.1r
PROTON.ucl CDCl3 {D:\500} vmc 27
f1 (ppm)

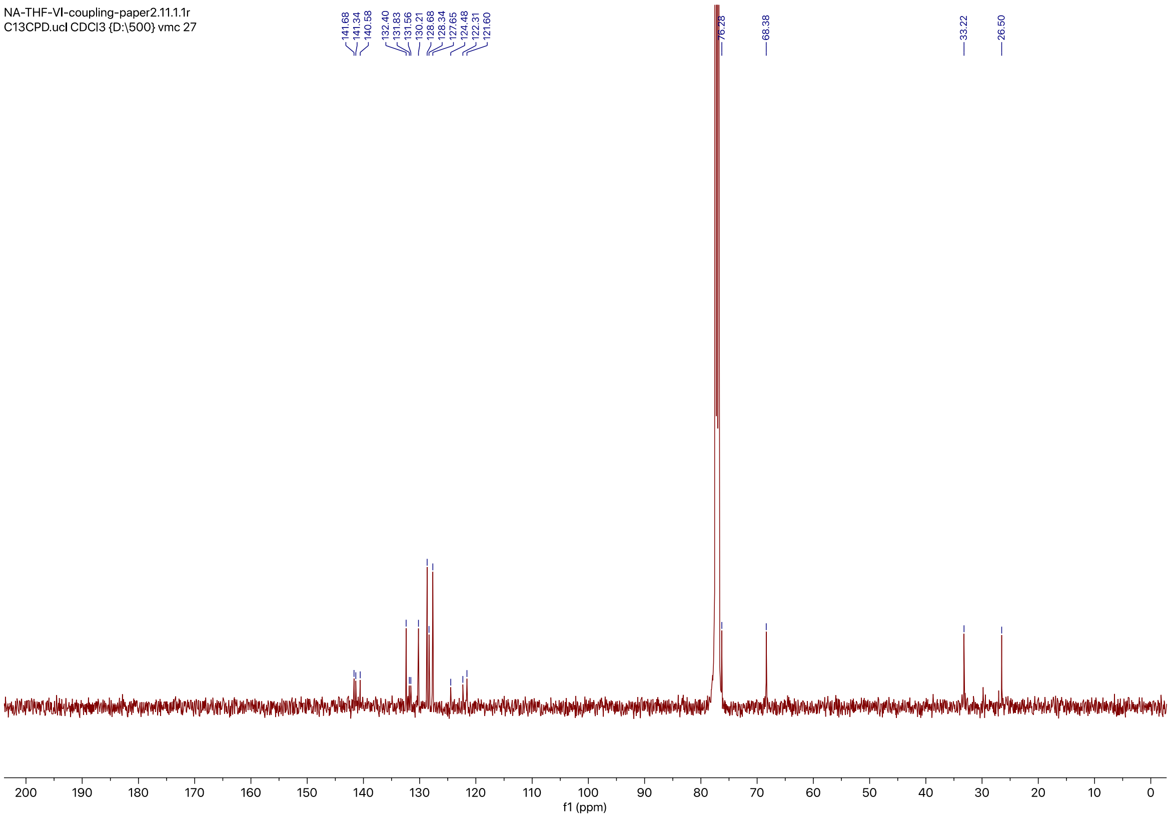
NA-THF-VI-coupling-paper2.11.1.1r
C13CPD.ucl CDCl3 {D:\500} vmc 27
f1 (ppm)

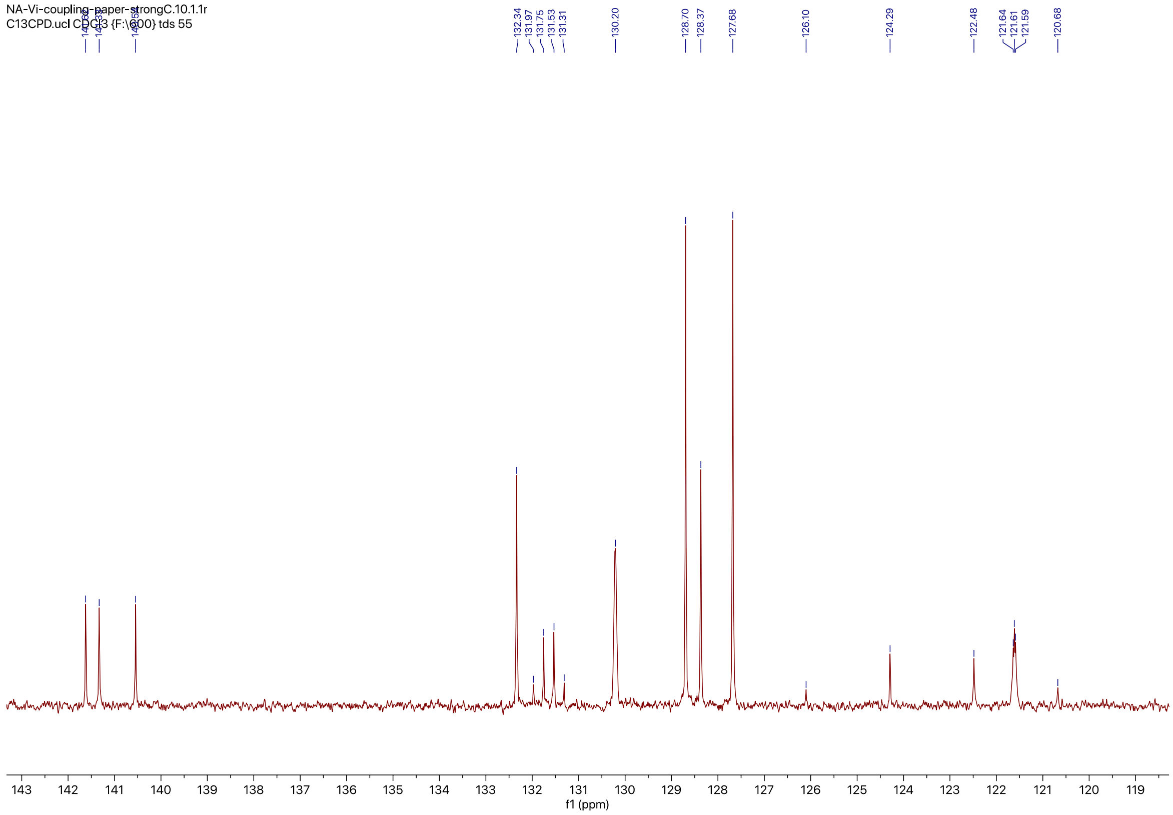
NA-Vi-coupling-paper-strongC.10.1.1r
C13CPD.ucl CDCl3 {F:\600} tds 55
141.60
141.33
140.54
132.34
131.97
131.75
131.53
131.31
130.20
128.70
128.37
127.68
126.10
124.29
122.48
121.64
121.61
121.59
120.68
143 142 141 140 139 138 137 136 135 134 133 132 131 130 129 128 127 126 125 124 123 122 121 120 119
f1 (ppm)

Mechanistic studies

^{13}C Labelled (((trifluoromethyl)sulfonyl)ethynyl)benzene – ^{13}C-6a

^{1}H NMR 700 MHz, $CDCl_3$) δ 7.71–7.68 (m, 2 H), 7.64–7.61 (m, 1 H), 7.50–7.47 (m, 2 H); ^{13}C NMR (126 MHz, $CDCl_3$) δ 133.9 (CH, d, *J* = 2.9 Hz), 133.5 (br s, CH), 129.2 (CH), 119.2 (C, qd, *J* = 323.1, 25.0 Hz), 115.92 (C, d, *J* = 10.6 Hz), 100.9 (m, C), **77.4** (^{13}C).

Synthesis as described above (Page 5)

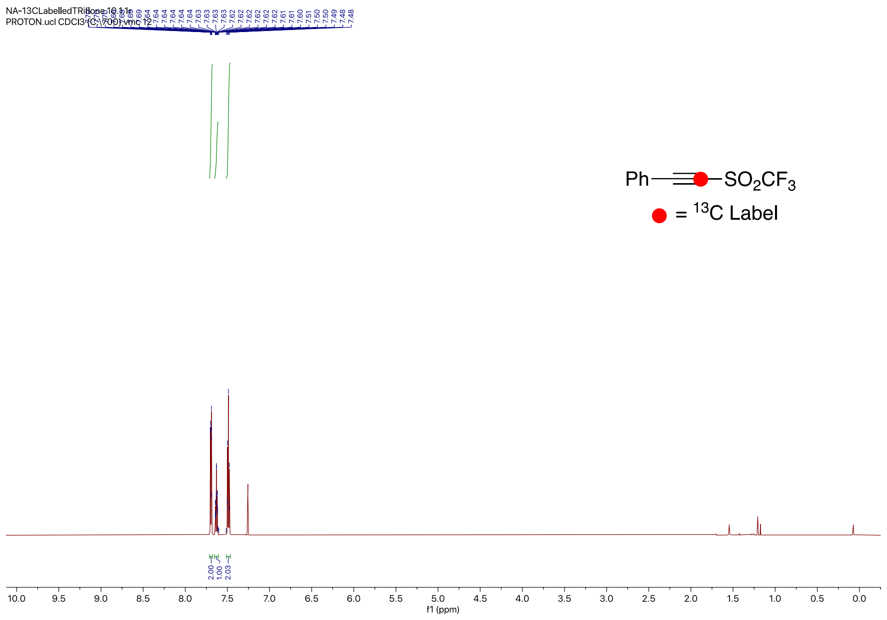

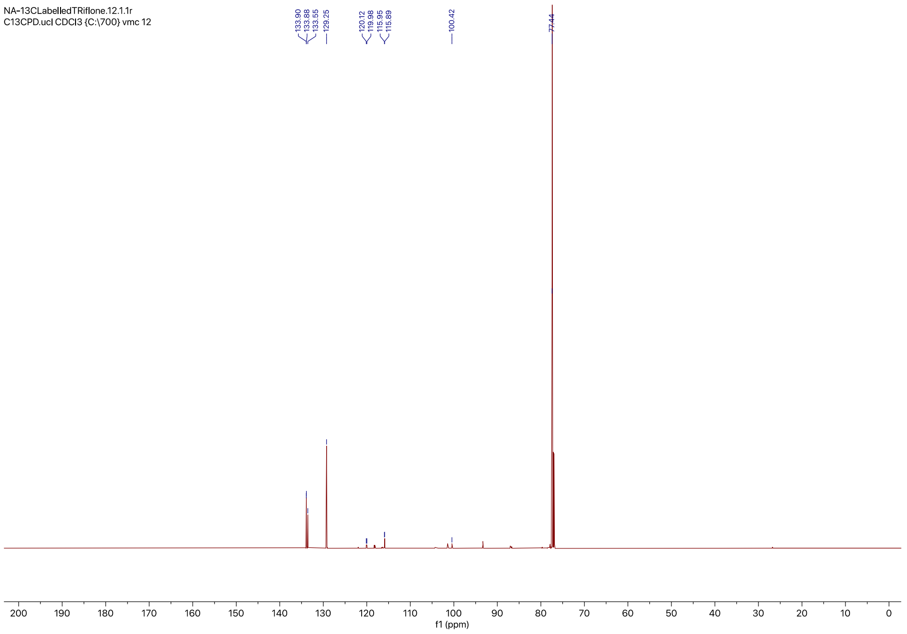

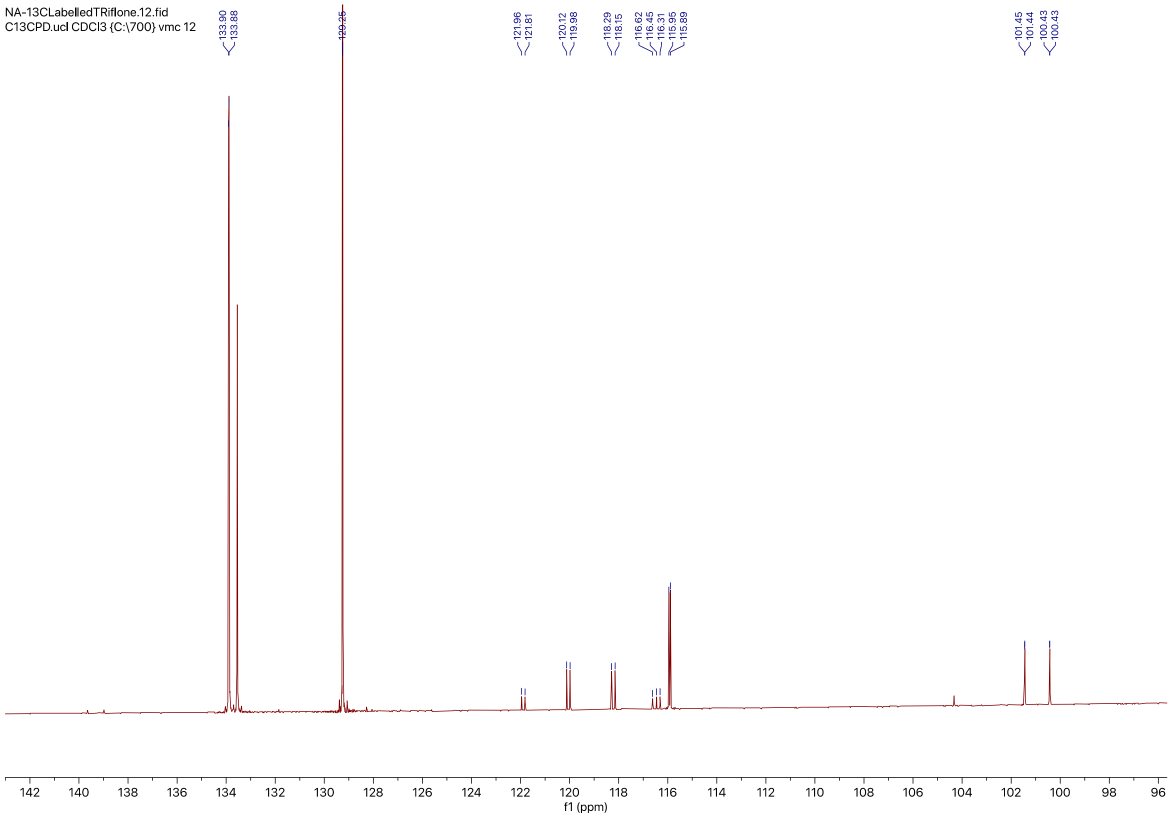
NA-13CLabelledTRiflone.12.fid
C13CPD.ucl CDCl3 {C:\700} vmc 12
133.90
133.88
129.25
121.96
121.81
120.12
119.98
118.29
118.15
116.62
116.45
116.31
115.95
115.89
101.45
101.44
100.43
100.43
142 140 138 136 134 132 130 128 126 124 122 120 118 116 114 112 110 108 106 104 102 100 98 96
f1 (ppm)

^{13}C Labelled 2-(phenylethynyl)tetrahydrofuran – ^{13}C-8a

^{1}H NMR ($CDCl_3$, 700 MHz): δ 7.45–7.41 (m, 2H), 7.34–7.29 (m, 3H), 4.81 (dt, J = 7.1, 4.9 Hz, 1H), 4.09–3.96 (m, 1H), 3.89–3.84 (m, 1H), 2.29–2.20 (m, 1H), 2.17–2.07 (m, 2H), 2.00–1.91 (m, 1H); ^{13}C NMR (176 MHz, $CDCl_3$) δ 131.9 (CH, d, J = 2.6 Hz), 128.4 (CH, br s), 128.3 (CH), 122.9 (C, J = 12.5 Hz), **89.2** (^{13}C), 86.02 (C, d, J = 355.7 Hz), 68.7 (CH, d, J = 77.5 Hz), 68.1 (CH_2, d, J = 2.1 Hz), 33.6 (CH_2), 25.6 (CH_2).

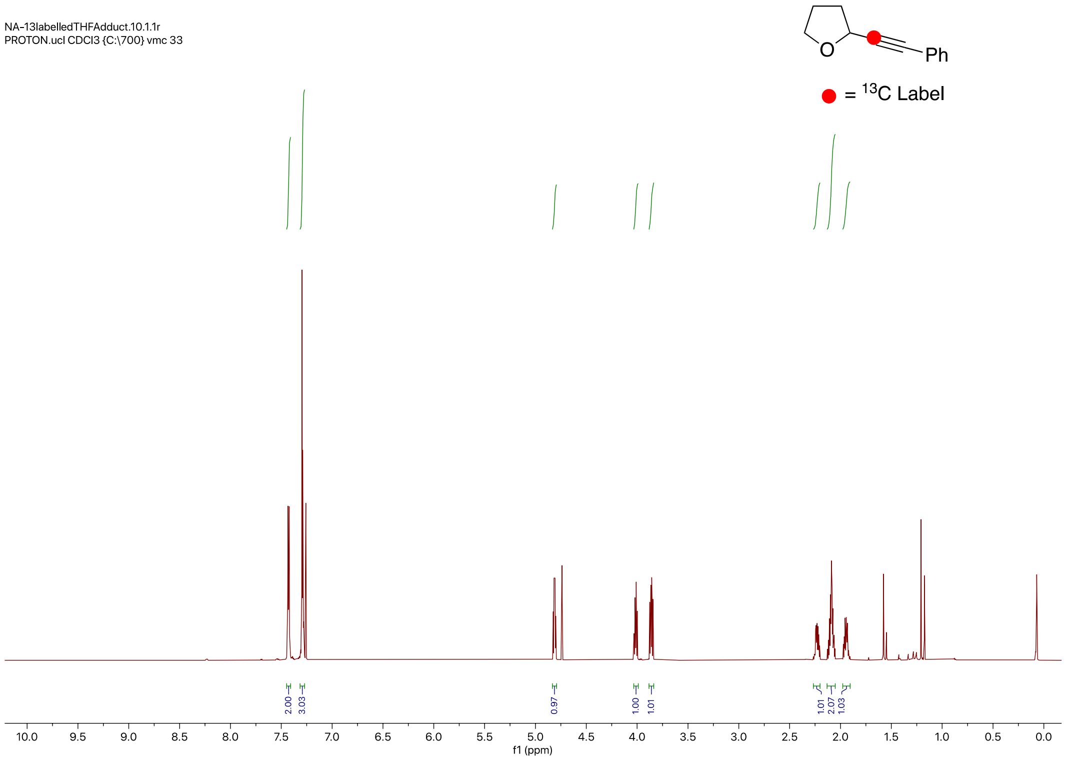

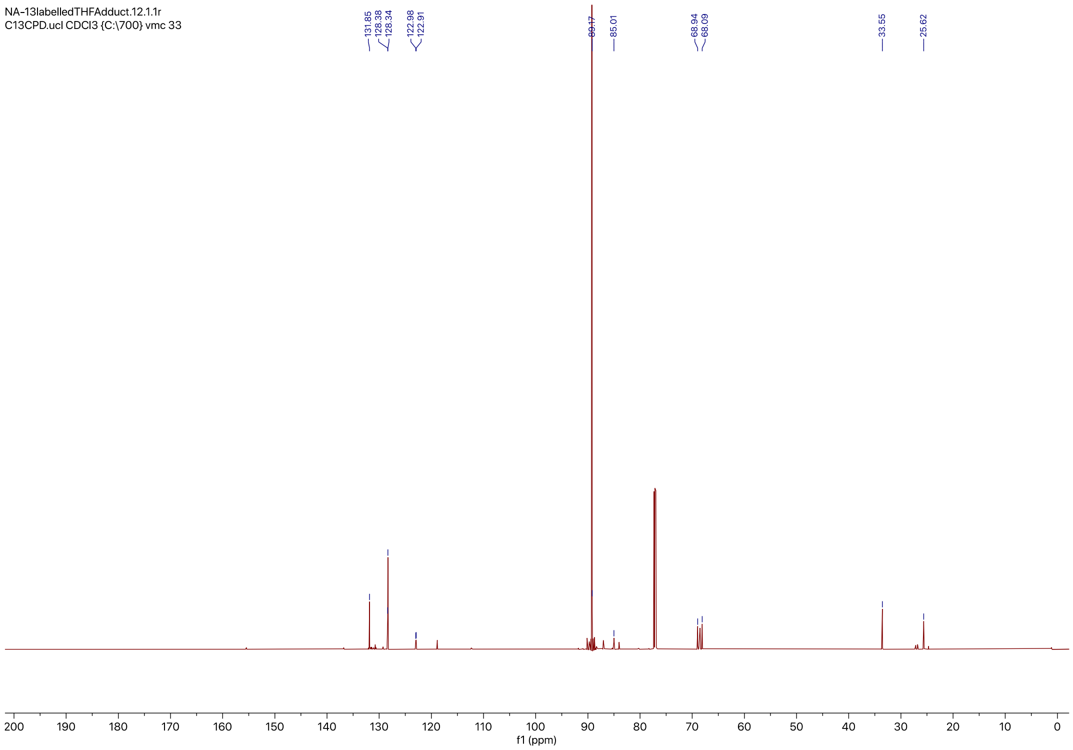

NA-13labelledTHFAdduct.12.1.1r
C13CPD.ucl CDCl3 {C:\700} vmc 33
131.85
128.38
128.34
122.98
122.91
89.17
85.01
68.94
68.09
33.55
25.62
200 190 180 170 160 150 140 130 120 110 100 90 80 70 60 50 40 30 20 10 0
f1 (ppm)

Kinetic Isotope Experiments

MS data for 2-(Phenylethynyl)tetrahydrofuran, non-deuterated

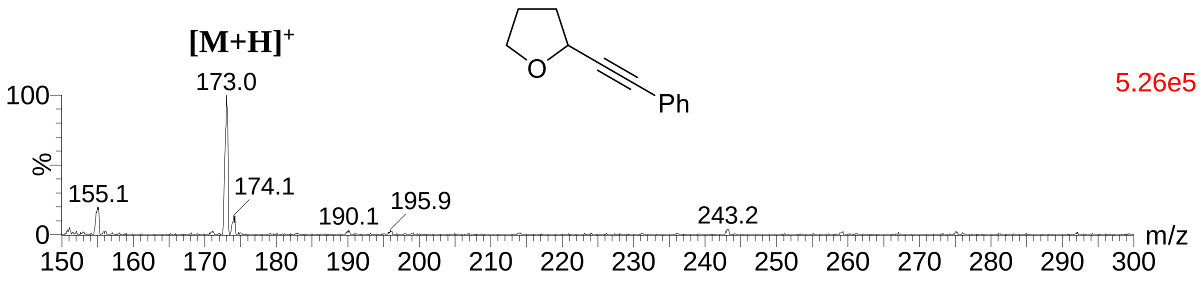

MS data for 2-(Phenylethynyl)tetrahydrofuran, deuterated

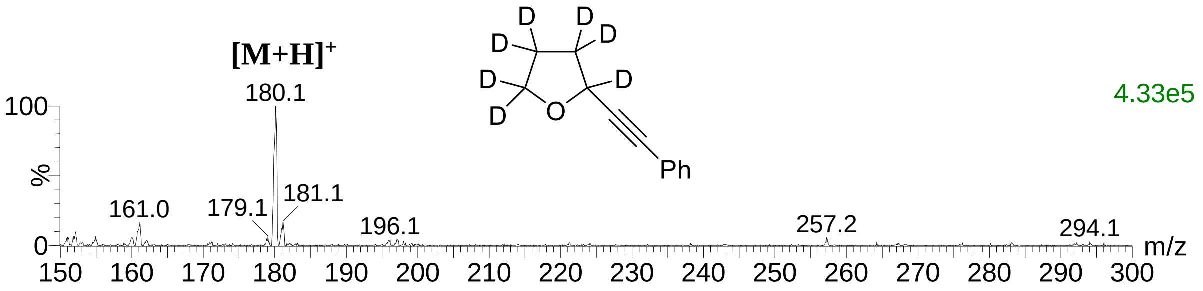

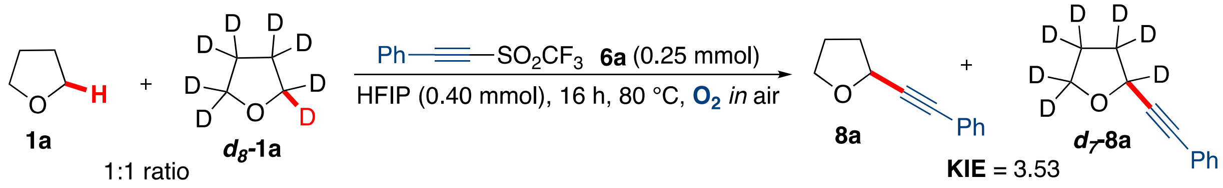

In a seal tubed containing (0.625 mmol) THF and (0.625 mmol) THF-*d8* was added HFIP (0.4 mmol) and (((trifluoromethyl)sulfonyl)ethynyl)benzene (60 mg, 0.25 mmol, 1.0 eq). The reaction was heated at 80 °C for 16 h. After this time, the product ratio was determined *via* LCMS analysis by integrating the peak areas of the extracted ion chromatograms (EICs) for the non-deuterated and deuterated species in a similar manner as described previously. [14]

LCMS Total Ion Count

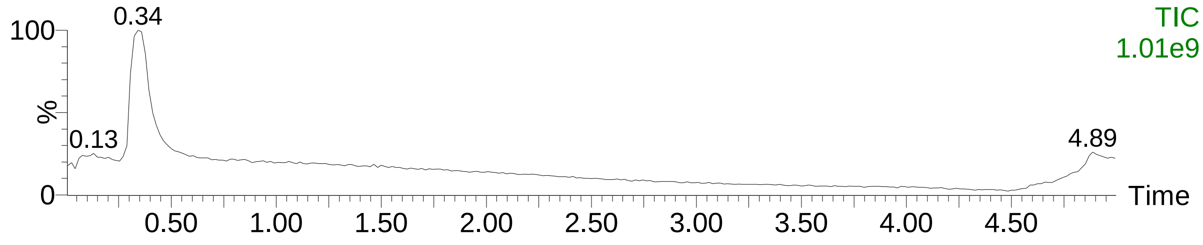

EIC at 173.23 Da (THF, non-deuterated H^+ adduct)

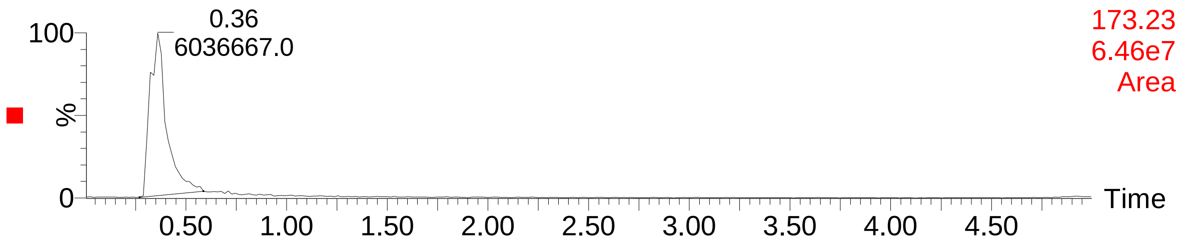

Height = 63715984

Area = 6036667.0

EIC at 180.27 Da (THF-d7 deuterated H^+ adduct)

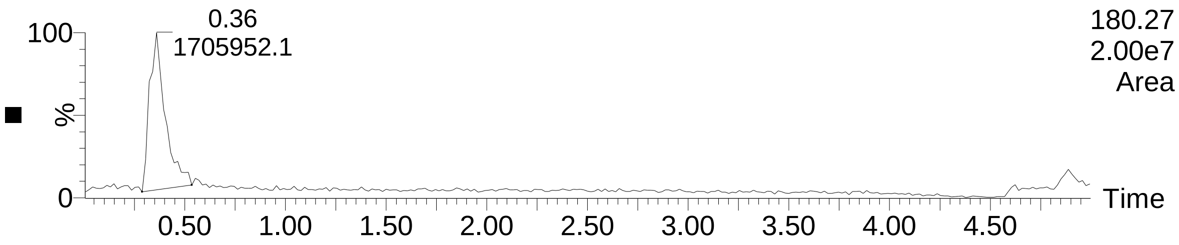

Height = 19001804

Area = 1705952.125

Adduct with H^+: d0/d7 = 6036667/1705952 = **3.53 = Kinetic Isotope effect**

Tempo Radical Trapping experiment:

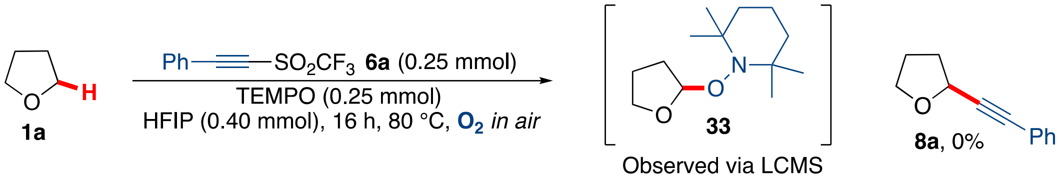

To a solution of THF (0.1 mL, 1.25 mmol, 5.0 eq.) in HFIP (0.4 mmol) was added (((trifluoromethyl)sulfonyl)ethynyl)benzene **6a** (60 mg, 0.25 mmol, 1.0 eq,) and TEMPO (0.25 mmol, 1.0 eq.). The reaction mixture was stirred at 80 °C for 16 h and then analysed *via* LCMS.

LCMS Total ion count

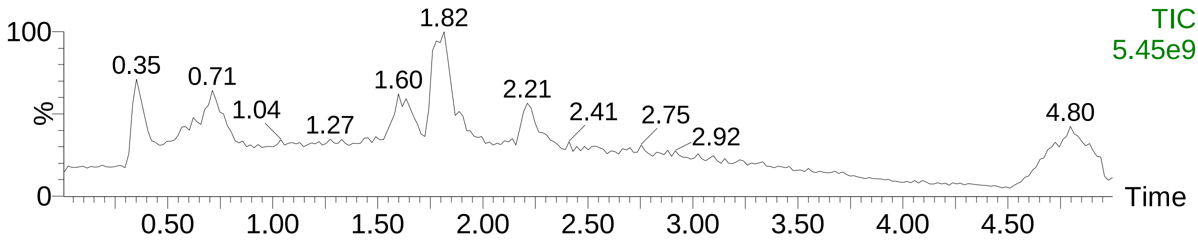

EIC at 228.0 Da

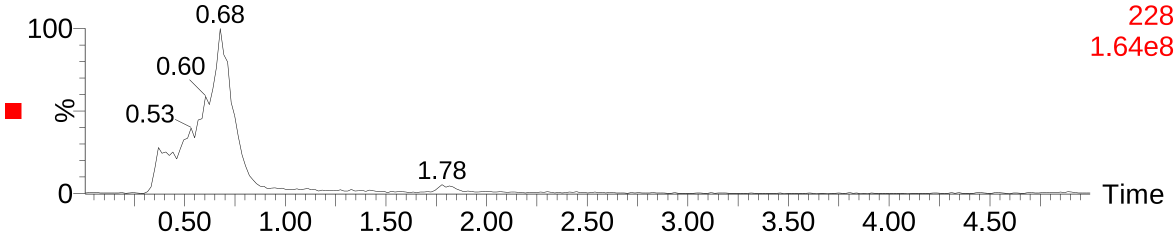

MS trace at 0.53-0.68

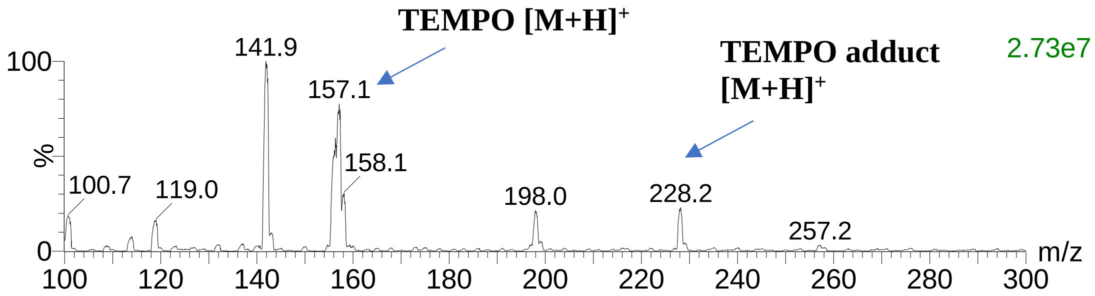

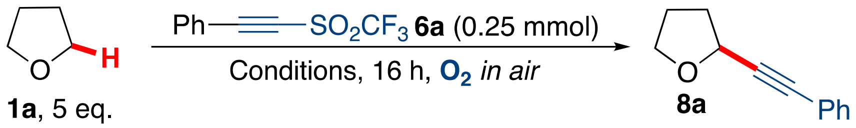

Entry	Temp	Solvent (in THF)	Other conditions	Yield (A) %	Triflone (%) Conversion
1	40	HFIP (0.40 mmol)	Control – No THF		0
2	60	HFIP (0.40 mmol)	Control – No THF		trace
3	80	HFIP (0.40 mmol)	Control – No THF		5
4	80	HFIP (2.0 mmol)		68	100
5	80	HFIP (2.0 mmol)	Control – No THF		18
6	80	HFIP (1.0 mmol)		78	100
7	80	HFIP (0.4 mmol)		93	100
8	66			41	80
9	80	CF_3CH_2OH (0.40 mmol)		49	90
10	80	*Tert*-C_4F_9OH (0.40 mmol)		32	100
11	80	AcOH (0.40 mmol)		42	
12	80	HFIP (0.40 mmol)	Argon	0	5
13	RT	HFIP (0.40 mmol)		0	0
14	40	HFIP (0.40 mmol)		38	44
15	60	HFIP (0.40 mmol)		53	60

Table S1 – Full optimisation table for radical C-H functionalisation of THF **1a** with phenyl acetylenic triflone **6a**.

References:

1) T. O. P. Hayes, B. Slater, R. A. J. Horan, M. Radigois and J. D. Wilden, *Org. Biomol. Chem.*, 2017, **15**, 9985-9902.
2) J. Zhang, P. Li and L. Wang, *Org. Biomol. Chem.*, 2014, **12**, 2969-2978.
3) Z. Ruo-Yi, X. L-Yi, Z. Lei, L. Shuai, C. Shan-Yong and Y. Xiao-Qi, *RSC Adv.*, 2014, **4**, 54349-54353.
4) L. Xu, R. Dai, D. Jie, C. Xu and L. Guigen, *Org. Lett.*, 2018, **20**, 6906-6909.
5) Q. Huang, D. Yifan, M. Kostiantyn, O. Doyle and P. Michael, *J. Org. Chem.*, 2017, **82**, 1584-1590.
6) W. Miao, M. Zhilin, L. Hongxiang and L. Lei, *Angew. Chem. Int. Ed.*, 2014, **53**, 14065-14069.
7) L. Qiren, D. Brabander and K. Jef, *Tetrahedron*, 2011, **67**, 27-28.
8) T. A. Mitchell and J. W. Bode, *J. Am. Chem. Soc.*, 2009, **131**, 18057-18059.
9) X. Xiaofei, L. Jie, W. Lei and W. Min, *Eur. J. Org. Chem.*, 2020, 1534-1538.
10) Y. Yuan, H. Hui, Z. Xiaoyun, Z. Weilan and L. Yun, *Synthesis*, 2013, 3137-3146.
11) R. S. Kim, L. V. Dinh-Nguyen, K. W. Shimkin and D. A. Watson, *Org. Lett.*, 2020, **22**, 8106-8110.
12) J. Xiang, W. Jiang, J. Gong and P. L. Fuchs, *J. Am. Chem. Soc.*, 1997, **119**, 4123-4129.
13) R. F. Robert-Andre, S. Matthew and V. Edwin, *J. Am. Chem. Soc.*, 2008, **130**, 9182-9183.
14) G. N. Papadopoulos, M. G. Kokotou, N. Spilipoulou, N. F. Nikitas, E. Voutyritsa, D. I. Tzaras, N. Kaplaneris and D. G. Kokotos, *ChemSusChem*, 2020, **13**, 5934-5944.
15) A. Roy, M. K. Das, S. Chaudhuri and A. Bisai, *J. Org. Chem.*, 2018, **83**, 403–421.
16) A. Mahadevan and P.L. Fuchs, *Tetrahedron Lett.*, 1994, **35**, 6025-6028.
